# Coordination mode engineering in stacked-nanosheet metal–organic frameworks to enhance catalytic reactivity and structural robustness

**DOI:** 10.1038/s41467-019-10547-9

**Published:** 2019-06-25

**Authors:** Chuanhui Huang, Juncai Dong, Weiming Sun, Zhenjie Xue, Jun Ma, Lirong Zheng, Cong Liu, Xiao Li, Kang Zhou, Xuezhi Qiao, Qian Song, Wende Ma, Lan Zhang, Zhenyu Lin, Tie Wang

**Affiliations:** 1 Beijing National Laboratory for Molecular Sciences, Key Laboratory of Analytical Chemistry for Living Biosystems and Colloid, Interface and Chemical Thermodynamics, Institute of Chemistry, Chinese Academy of Sciences, #2 Zhongguancun, North First Street, Beijing, 100190 China; 2 Ministry of Education Key Laboratory for Analytical Science of Food Safety and Biology, Fuzhou University, Fuzhou, Fujian 350116 China; 3 University of Chinese Academy of Sciences, Beijing, 100049 China; 4 Beijing Synchrotron Radiation Facility, Institute of High Energy Physics, Chinese Academy of Sciences, 19 B Yuquan Rd, Beijing, 100049 China; 5 The Department of Basic Chemistry, The School of Pharmacy, Fujian Medical University, Fuzhou, Fujian 350108 China; 6 State Key Laboratory of Structure Chemistry, Fujian Institute of Research on the Structure of Matter, Chinese Academy of Sciences, Fuzhou, Fujian 350002 China

**Keywords:** Heterogeneous catalysis, Two-dimensional materials, Organic-inorganic nanostructures

## Abstract

Optimising the supported modes of atom or ion dispersal onto substrates, to synchronously integrate high reactivity and robust stability in catalytic conversion, is an important yet challenging area of research. Here, theoretical calculations first show that three-coordinated copper (Cu) sites have higher activity than four-, two- and one-coordinated sites. A site-selective etching method is then introduced to prepare a stacked-nanosheet metal–organic framework (MOF, CASFZU-1)-based catalyst with precisely controlled coordination number sites on its surface. The turnover frequency value of CASFZU-1 with three-coordinated Cu sites, for cycloaddition reaction of CO_2_ with epoxides, greatly exceed those of other catalysts reported to date. Five successive catalytic cycles reveal the superior stability of CASFZU-1 in the stacked-nanosheet structure. This study could form a basis for the rational design and construction of highly efficient and robust catalysts in the field of single-atom or ion catalysis.

## Abstract (web summary)

Engineering the coordination mode of atom or ion onto the substrate remains challenging. Here, guided by theoretical calculation, the authors prepare stacked-nanosheet MOF based catalyst with precisely controlled coordination sites on the surface and enhanced catalytic reactivity and structural robustness.

## Introduction

Monodispersed single atoms and ions on solid substrates, as unsaturated coordinated metal sites, have recently emerged as an exciting class of catalysts that combine the merits of both homogeneous and heterogeneous catalysts^1–3^. However, most studies to date used specific materials as supporting substrates to anchor the active metal site, which prohibited the further optimisation of catalytic activity^4,5^. Metal–organic frameworks (MOFs), which are formed by coordination bonds between metal nodes and organic linkers, have tailored structures and great functional tunability^6,7^. Therefore, MOFs may be good candidates for exploration of the relationship between the catalytic performance and anchored mode of metal sites. Some studies have reported specific functionalities arising from open metal sites or certain defects, e.g., under-coordinated metal sites, such as improved adsorption affinity or catalytic activity^8–12^. However, major challenges for further improved design and construction of future generations of unsaturated coordinated metal sites are the production of a series of metal sites with systematically tunable coordination modes, and the establishment of a definitive correlation between the catalytic properties and coordination state. Furthermore, the robustness of a framework is often vulnerable to changes in the metal–ligand interactions. Frameworks with under-coordinated metal sites collapse easily upon thermal or catalytic treatment^13–15^. These limitations represent a major challenge in establishing the exact structure-to-property correlation in coordinatively unsaturated metal atoms for catalysis, which is essential for the rational design and synthesis of coordinatively unsaturated metal sites with tailored activities under the premise of ensuring stability of the structure.

In this study, we take inspiration from the hexagonal-shaped cells of a honeycomb, which provide relatively high out-of-plane compression and shear properties^16,17^ and develop a strategy for etching stacked-nanosheet MOFs (CASFZU-1) with three-coordinated copper (Cu) by selectively etching the classical HKUST-1 along the (111) facets. Measurements of the catalytic efficiency of stacked-nanosheet MOFs show a higher turnover frequency (TOF) for CO_2_ fixation with large epoxides (i.e., 2-octyloxirane and 1,2-epoxydodecane) compared to most other catalysts reported to date. In addition, they have a long catalytic lifetime of four recovery cycles and bulk production yields several kilograms at a time. Therefore, such MOFs are potentially suitable as practical catalysts and are promising candidate materials for industrial applications.

## Results

### Design and theoretical prediction

**Fig. 1:**
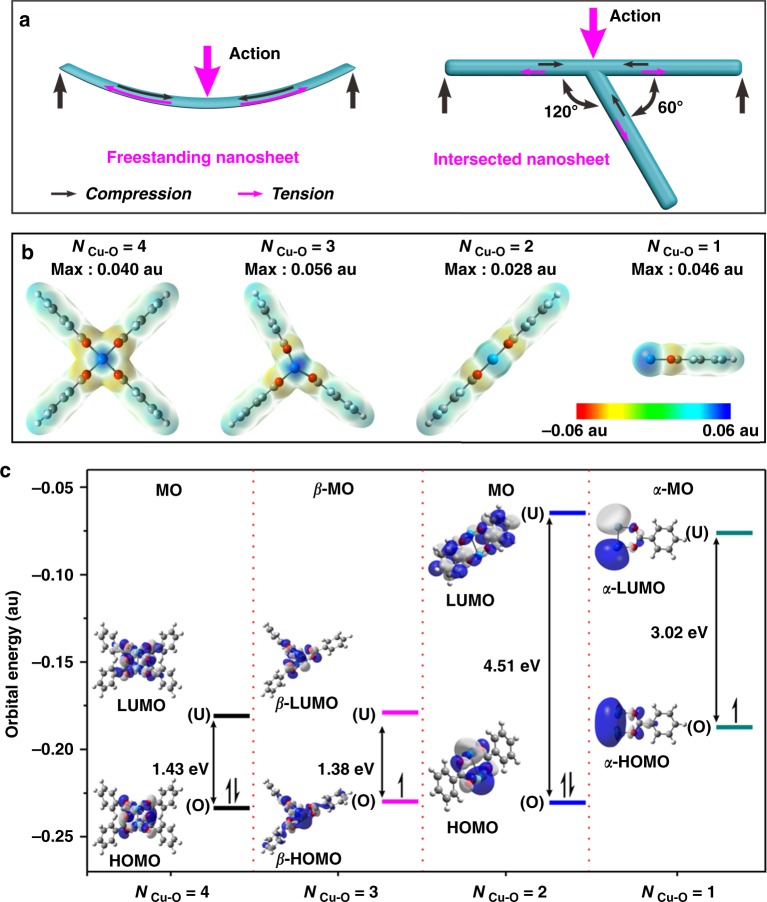
Structural stability and physical properties of paddlewheel Cu_2_ clusters. **a** A schematic overview of displacements for the freestanding metal–organic framework (MOF) nanosheet and intersected-nanosheet under the same acting force. **b** Electrostatic surface potential (ESP) maps of paddlewheel Cu_2_ clusters. *N*_Cu-O_ represents the paddlewheel Cu_2_ clusters with different coordination numbers (CNs) of Cu–O (4, 3, 2, 1) (isovalue = 0.001 e per bohr^3^). **c** The simulated frontier orbital energy levels and molecular orbital (MO) diagrams (isovalue = 0.02 au) of paddlewheel Cu_2_ clusters with different CNs of Cu–O (4, 3, 2, 1). Only the *β*-MO and *α*-MO with smaller energy gaps are shown for the open-shell three- and one-coordinated copper (Cu) catalysts. Colour scheme for chemical representation: cyan, Cu; red, O; grey, C; white, H

While the nanosheets intersect at lines converging at angles of 60° and 120°, these triangular or hexagonal structures prevent the material from collapsing by evenly distributing stress loads^18^. Therefore, in comparison to freestanding MOF nanosheets, we obtained an MOF that is more stable under the thermodynamic and kinetic conditions that arise during the catalytic reaction (Fig. 1a). In addition to enhancing the activities of Cu sites, the three-coordinated Cu sites on the surface of the stacked MOF nanosheets also increased the affinity between the active sites and the reactant. As shown in Fig. 1b and Supplementary Figure 1, showing the electrostatic surface potential (ESP) diagrams obtained from density functional theory (DFT)-based calculations, the activities of the three-coordinated Cu (0.056 au) with more *σ*-holes were higher than those of pristine four-coordinated Cu (0.040 au) and of both two-coordinated and one-coordinated Cu (0.028 and 0.046 au, respectively)^19^. We analysed the frontier molecular orbitals (MOs)^20^, according to which the three-coordinated Cu sites have the smallest gap between their highest occupied molecular orbital (HOMO) and lowest unoccupied molecular orbital (LUMO) of 1.38 eV, whereas the four-, two- and one-coordinated sites have HOMO-LUMO gaps of 1.43 eV, 4.51 eV, and 3.02 eV, respectively (as shown in Fig. 1c). This suggests that three-coordinated Cu is superior for applications involving covalent bonding with a soft base^21^.

### Synthesis and characterisation

**Fig. 2:**
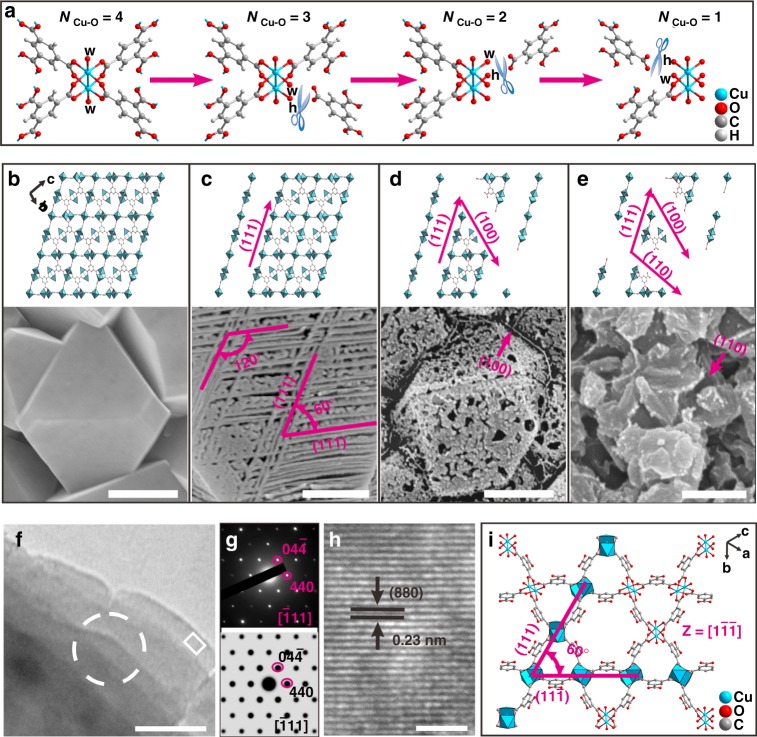
Synthetic strategy and structural characterisation. **a** Schematic overview of the etching transformation of four-coordinated Cu atoms to three-, two- and one-coordinated Cu atoms with organic ligands (w represents oxygen atom from the water, and h represents oxygen atom from the hydroxide anion, OH^−^). **b**–**e** Model structures and real morphologies of the four different MOF crystal states after etching. **b** Pristine HKUST-1 MOFs, **c** stacked-nanosheet MOFs, **d** random-etching MOFs, **e** collapsed MOFs. **f** Transmission electron microscopy (TEM) top-view image of a five-layered CASFZU-1 thin nanosheet. **g** Selected area electron diffraction (SAED) pattern (taken from the white-circled region in **f**) at the top shows diffraction within a few-layered nanosheet. A simulated SAED pattern of the HKUST-1 crystal down the $[\bar 111]$ axis is also shown. **h** High-resolution TEM (HR-TEM) image of the white-framed region at the edge of the nanosheet shown in **f**. **i** The crystal structure of HKUST-1 along the $[1\bar 1\bar 1]$ direction. Scale bars represent 500 nm for **b**, **d**, and **e**, 200 nm for **d**, 100 nm for **f** and 2 nm for **h**. Colour scheme for chemical representation: cyan, Cu; red, O; grey, C; white, H

We used a two-step stacked-nanosheet MOF (CASFZU-1) preparation method (Fig. 2a). First, pristine HKUST-1 film was synthesised on a nylon 66 membrane as described previously with some modifications^22^ (Supplementary Figures 1–5), and second the as-prepared HKUST-1 film was etched controllably by water vapour and stored in a container at 25% relative humidity (RH) at room temperature for a few days (Supplementary Figures 5 and 6), accompanied by mesopores ranging from a few to several hundreds of nanometres in size.

The formation of the CASFZU-1 crystal followed a surface energy-driven mechanism. The interaction energy between metal ions and carboxyl O atoms was similar to that between metal ions and water O atoms. The coordinated ligands in the MOF crystal were readily replaced by water molecules^23^. Calculations indicated that the surface energies of the (111), (110) and (100) planes in the HKUST-1 face-centred-cubic crystal followed the order ***γ***_**(111)**_ **<** ***γ***_**(100)**_ **<** ***γ***_**(110)**_ (Supplementary Figures 7–9 and Supplementary Note 1). A series of etched products were obtained by altering the etching conditions and by adjusting the RH and solvent polarity (SP) (Fig. 2b–e, Supplementary Figures 10–14, Supplementary Table 1). HKUST-1 is stable in the absence of liquid water or at low RH (i.e., 15% RH) and the Cu sites were all four-coordinated by ligands (Fig. 2b). At 25% RH, when the SP ranged from 4.30 to 7.25 (Supplementary Figures 11 and 14)^24^, the MOF film composed of MOF nanosheets 9.25 ± 0.28 nm thick (ca. 2-unit cell thickness) (Supplementary Figure 15) retained its structural integrity (Fig. 2c, Supplementary Figures 16 and 17). We exposed the three-coordinated Cu sites by strictly limiting etching along the (111) facets of the crystal structure (Supplementary Figure 18). The angles between two lines along the intersection between nanosheets that intersect at three (111) facets measure 60°, and the dihedral angle formed by the facets of each of the adjacent sheets is 70.529° (Supplementary Figure 18). We identified the perfect (111) crystalline facets by measuring the corresponding selected area electron diffraction (SAED) pattern (Fig. 2g) and analysing high-resolution transmission electron microscopy (HR-TEM) images. The lattice fringes at interplanar distances of 0.23 nm corresponded to the *d*_*880*_ planes of the MOF crystal (Fig. 2h, i, Supplementary Figure 19). As SP was increased to 10.2, the (100) facets of the octahedral crystals became unstable, and the (111) and (100) facets were both etched to form anisotropically shaped pores from randomly etched MOFs (Fig. 2d), predominated by three-, two- and one-coordinated Cu sites. Large surface relaxations may occur when any Cu atom is coordinated with one or two benzene-1,3,5-tricarboxylate (TMA) linkers, which would decrease the stability of the MOF network. Further, when the RH exceeded 50%, all facets of the crystal were l destroyed (Fig. 2e), resulting in large cleavages and structural collapse (denoted as collapsed MOFs) (Supplementary Figure 20).

The replacement of organic ligands by water molecules was verified for pristine and etched MOF films using energy-dispersive X-ray spectroscopy (EDX) mapping and X-ray photoelectron spectroscopy (XPS). These techniques established that CASFZU-1 had 59.25%, 46.7%, and 94.9% more Cu on the surface than pristine HKUST-1, random-etching and collapsed MOFs, respectively. (Supplementary Figures 21 and 22 and Supplementary Table 2). The shift in the peak corresponding to Cu (II) species undergoing transformation to higher energy (from 940.3 to 940.8 eV) indicated that the Cu ions remained divalent but become more active in CASFZU-1 (Supplementary Figure 22). These Cu species in CASFZU-1 were visibly and rapidly reduced to the Cu crystal by electron beam irradiation during TEM (Supplementary Figure 23). On in situ Fourier transform-infrared (FT-IR) spectra (Supplementary Figure 24), the gradual increases in intensity of the bands at 1704 and 1277 cm^−1^ corresponding to the C = O and C–OH combination bands of a carboxylic acid, respectively, indicated transformation of the Cu–TMA carboxylate groups to their protonated acid analogues^25^. Compared to the pristine HKUST-1 (1550 m^2^ g^−1^), the BET surface area for CASFZU-1 decreased to 1043 m^2^ g^−1^, suggesting that part of the inherent porous structure was blocked after some ligands fell off the MOF. In contrast to site-selective etching, faster degradation kinetics resulting in porosity of the MOFs was *ca*. 60%, and completely lost for random-etching MOFs (638 m^2^ g^−1^) and collapsed MOFs (7.0 m^2^ g^−1^), respectively (Supplementary Figure 25).

### Atomic structure analysis of copper site

**Fig. 3:**
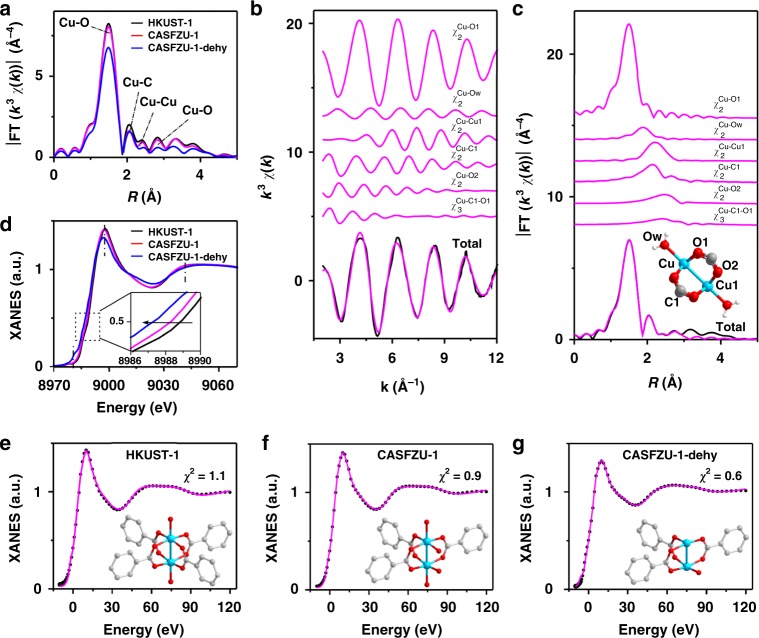
Structural characterisation by EXAFS and XANES spectroscopy. **a** Fourier transformed magnitudes of the experimental K-edge EXAFS signals for HKUST-1 and CASFZU-1 samples before and after dehydration (denoted as CASFZU-1-dehy). The Fourier transforms are not corrected for phase shift. The origin assignment for the EXAFS peaks is given in Supplementary Figure 26. **b**, **c** Cu K-edge EXAFS analysis of CASFZU-1 after dehydration in *k* and *R* spaces, respectively. Curves from top to bottom are the single backscattering signal *χ*_2_ and double scattering signal *χ*_3_ included in the fit, and the total signal (red line) superimposed on the experimental signal (black line). The inset shows the structure of the hydrated [Cu_2_C_4_O_8_](H_2_O)_2_ moiety used for simulation of the EXAFS signals. **d** The experimental Cu K-edge XANES spectra. **e**–**g** Comparison between the experimental XANES spectra (black dotted lines) and the best-fit theoretical spectra with the depicted structural models (solid red lines). **e** The hydrated [Cu_2_C_4_O_8_](H_2_O)_2_ cage. **f** The defective three-coordinated [Cu_2_C_3_O_6_](H_2_O)_3_OH cage. **g** The dehydrated three-coordinated [Cu_2_C_3_O_6_]OH paddlewheel model, where one of the two coordinatively unsaturated Cu atoms is directly bound by one hydroxide anion, OH^−^. Colour scheme for chemical representation: cyan, Cu; red, O; grey, C and white, H

To confirm the etching transformation process from HKUST-1 to CASFZU-1 proposed in Fig. 2a, the evolution of the local atomic arrangements and chemical state of Cu species were investigated by X-ray absorption spectroscopy (XAS), including extended X-ray absorption fine structure (EXAFS) and X-ray absorption near-edge structure (XANES). The etching and dehydration effects were first identified by EXAFS analysis. Figure 3a shows the EXAFS Fourier transform (FT) magnitude of CASFZU-1 before and after dehydration (denoted as CASFZU-1-dehy), along with the pristine HKUST-1. Their EXAFS spectra exhibited similar envelopes featuring a main peak at 1.50 Å arising from Cu–O bonding as well as several minor peaks located at 2.05, 2.41, and 2.83 Å dominated by higher-shell Cu–C, Cu–Cu and Cu–O scattering, respectively (Supplementary Figure 26). When etching HKUST-1 to CASFZU-1, the Cu–O and Cu–Cu peaks showed subtle changes, whereas the Cu–C peak exhibited an obvious decrease in intensity (Fig. 3a), which could be attributed to the partial removal of carboxylate ligand. In contrast, further dehydration treatment of CASFZU-1 resulted in remarkable decreases in intensity for the main Cu–O peak, as well as the higher-shell Cu–Cu and Cu–O peaks, with a slight change for the Cu–C peak (Fig. 3a). The underlying structural modifications were then extracted by quantitative EXAFS curve fitting analysis (see Methods). Our EXAFS analysis was first validated by the distortion of HKUST-1 upon dehydration (Supplementary Figures 27–31 and Supplementary Table 3); while all the scattering path lengths concerning the [Cu_2_C_4_O_8_] framework cage showed obvious shrinking, the Cu–Ow bond from water molecules showed a slight decrease in the coordination number (CN) and a considerable increase in the Debye–Waller factor, in good agreement with previous results^26^. However, it is interesting that the etching of HKUST-1 to CASFZU-1 produced visible elongation for the higher coordination shells (i.e., Cu–C1, Cu–Cu1 and Cu–O2) (Supplementary Figure 32 and Supplementary Table 3), suggesting expansion for the [Cu_2_C_4_O_8_] framework cage. Moreover, the decreased CNs for the ligand-related paths confirmed etching of the carboxylate ligand on the surface of nanosheet CASFZU-1. The following dehydration treatment of CASFZU-1 caused contraction for all scattering path lengths (Fig. 3b, c and Supplementary Table 3), suggesting further shrinkage of the [Cu_2_C_4_O_8_] framework cage, consistent with the distortion behaviour of HKUST-1 upon dehydration.

To better identify the defective Cu site structures, we then used XANES spectroscopy, which is more sensitive to the 3D arrangement of atoms around the photoabsorber. Figure 3d shows XANES profiles for HKUST-1, CASFZU-1 and CASFZU-1-dehy, which are characterised by a pre-edge peak at 8978 due to 1 s → 3d transition, a shoulder peak at 8986 eV ascribed to 1 s → 4p dipolar shakedown transition, a white-line peak at 8,998 eV and a resonance peak at 9042 eV. In contrast to the high degree of resemblance of CASFZU-1 to HKUST-1, CASFZU-1-dehy showed an apparent intensity increase for the pre-edge and shoulder peaks, as well as a considerable intensity decrease for the while-line peak, indicating thorough removal of water molecules^26^, consistent with the marked colour changes in the samples (Supplementary Figure 33). Meanwhile, the progressive shift of the edge position toward the lower energy direction, along with etching and dehydration (see inset in Fig. 3d), indicated a gradually reduced oxidation state for the Cu species. The XANES spectra were then quantitatively analysed with the MXAN procedure to statistically discriminate the various plausible coordination models for Cu species (Supplementary Figure 34) based on fitting quality^27–29^ (i.e., the residue function *R*_sq_) (see Methods). The experimental XANES spectra for all the samples could be well reproduced by the hydrated [Cu_2_C_4_O_8_](H_2_O)_2_ cage model (Fig. 3e, Supplementary Figures 35–37 and Supplementary Table 4), which not only indicated their dominance in the MOF framework but also validated our analysis. Upon dehydration, the dehydrated [Cu_2_C_4_O_8_]_2_ cage model showed better agreement with the experimental spectrum of CASFZU-1-dehy (*R*_sq_ = 0.6) than that of HKUST-1-dehy (*R*_sq_ = 2.0) (Fig. 3g and Supplementary Figure 35), consistent with more thorough removal of water molecules in the former. Importantly, we found that excellent agreement between the experimental and theoretical spectra for both CASFZU-1 and CASFZU-1-dehy could be obtained by the defective three-coordinated [Cu_2_C_3_O_6_](H_2_O)_3_OH paddlewheel model (Fig. 3f, g and Supplementary Figures 36–37), indicating their presence on the exposed surfaces of CASFZU-1 nanosheets. Furthermore, the experimental spectrum for CASFZU-1-dehy could be satisfactorily reproduced by the dehydrated three-coordinated [Cu_2_C_3_O_6_]OH paddlewheel model where one of the two coordinatively unsaturated Cu atoms was directly bound by one hydroxide anion (OH^−^), suggesting that the three-coordinated structure can readily lose partial coordinated water molecules (Fig. 3g). Finally, it was noted that the bond metrics derived based on the hydrated [Cu_2_C_4_O_8_](H_2_O)_2_ cage model clearly exhibited expansion for the Cu paddlewheel when etching HKUST-1 to CASFZU-1 and shrinkage by the successive dehydration of CASFZU-1, in good agreement with the structural variations determined by EXAFS analysis and DFT prediction (Supplementary Figures 38 and 39 and Supplementary Table 4). Therefore, our comprehensive structural analyses not only provided solid XAS spectroscopic fingerprints for the defective three-coordinated [Cu_2_C_3_O_6_](H_2_O)_4_ paddlewheel model on the exposed surfaces of CASFZU-1 nanosheets, but also unambiguously revealed distortion during the etching transformation process from HKUST-1 to CASFZU-1.

### Catalysed CO_2_ cycloaddition

**Fig. 4:**
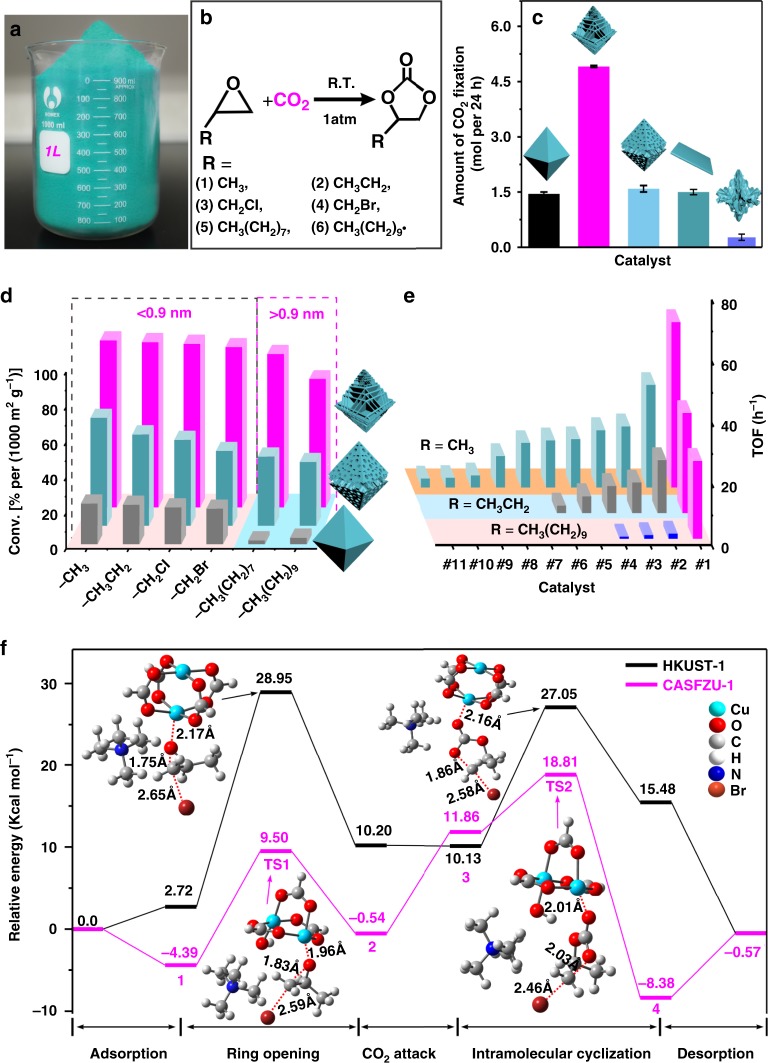
Catalytic activity and density functional theory (DFT) calculations. **a** Photographs of the *ca*.1.0 kg of CASFZU-1 prepared in the laboratory. **b** Scheme for cycloaddition of CO_2_ with epoxides to produce cyclic carbonates catalysed by CASFZU-1. **c** The amount of CO_2_ fixation with 2-methyloxirane by chemical conversion reactions catalysed by 1 g of different MOFs in 24 h. From left to right, the columns represent MOF crystals of pristine HKUST-1, CASFZU-1 and random-etching MOFs, HKUST-1 nanosheet and collapsed MOF forms. Error bars represent the standard deviation of three replicate samples. **d** Conversion for cycloaddition of CO_2_ with the six epoxides catalysed by HKUST-1 (grey), random-etching MOFs (light blue) and CASFZU-1 (red) after normalising BET. Columns from left to right represent 2-methyloxirane; 2-ethyloxirane; 2-(chloromethyl)oxirane; 2-(bromomethyl)oxirane; 2-octyloxirane; and 1, 2-epoxydodecane. **e** Turnover frequency (TOF) comparison of CASFZU-1 with the other catalysts in representative publications on catalysis of the cycloaddition reaction of CO_2_ with 2-methyloxirane, 2-ethyloxirane and 1,2-epoxydodecane (see Supplementary Tables 7–9 for details). The rightmost red columns represent CASFZU-1. **f** The computed reaction energy profiles of the CO_2_ fixation reaction on HKUST-1 and CASFZU-1. TS, transition state. The pathways are divided into five steps: (i) adsorption of epoxy; (ii) epoxy ring opening step; (iii) CO_2_ electrophilic attack step; (iv) intramolecular cyclisation step; (v) product desorption (see Supplementary Figures 49, 51 and 52 for more details and references). Colour scheme for chemical representation: cyan, Cu; red, O; grey, C; tangerine, Br; blue, N; white, H

We prepared quantities of CASFZU-1 on the order of kilograms using our method, which is reliable and simple to perform (Fig. 4a). We significantly improved the rate of cyclic carbonate production with strong Lewis acid Cu sites in CASFZU-1 (Supplementary Figure 40) and high affinity for CO_2_ (Fig. 4b, Supplementary Figures 41 and Supplementary Note 2 and Supplementary Table 5). CASFZU-1 exhibited the best catalytic reaction performance (Fig. 4c and Supplementary Figures 42–44) of the five different MOFs examined. In carbon fixation with 2-methyloxirane, CASFZU-1 converted a comparable amount of CO_2_ per gram to that accumulated by 210 m^2^ of undisturbed tropical rainforest^30^ (Supplementary Note 3 and Supplementary Figure 45). Notably, the experiments showed that the co-catalyst tetrabutylammonium bromide played a vital role in the ring-opening step for cyclic carbonate synthesis, and no significant catalytic activity was observed in the absence of tetrabutylammonium bromide. After normalising the BET, the cycloaddition yields of small molecules catalysed by CASFZU-1 were about four and two times higher than those of HKUST-1 and random-etching MOFs, respectively (Fig. 4d and Supplementary Figure 25). The advantages of stacked-nanosheet MOFs are greater for large-sized molecules. CASFZU-1 has large mesopores (4.0 nm), resulting in conversion efficiencies of 87.3% ± 1.2% and 72.9% ± 3.0% for 2-octyloxirane and 1,2-epoxydodecane, respectively (Fig. 4d, Supplementary Figure 46 and Supplementary Table 6), whereas these were 2.2% ± 0.5% and 3.7% ± 1.3% for HKUST-1 (with the largest entrance pore of 0.9 nm)^31^; these conversion efficiencies were only 39.2% ± 1.9% and 36.1% ± 2.4%, respectively, in random-etching MOFs (mesopore size, 3.6 nm). As the random-etching MOFs have similar mesopore size to CASFZU-1, these marked differences in catalytic efficiency between them may have been because the three-coordinated Cu sites have the highest activity among the four coordination states. The catalytic cycloaddition reaction of CO_2_ with 2-methyloxirane and 2-ethyloxirane had TOF of 54.0 h^−1^ and 32.7 h^−1^ in CASFZU-1, respectively, and these catalytic efficiencies were more than twofold higher than the most efficient Cu-based MOFs previously reported (Fig. 4e, Supplementary Tables 7 and 8). Furthermore, CASFZU-1 exceeded the maximum TOF for large substrate (1,2-epoxydodecane, TOF = 25.34 h^−1^) by 20-fold, because the large mesopores promote mass-transformation (Fig. 4e and Supplementary Table 9).

The high catalytic efficiency of CASFZU-1 was attributable to three aspects. First, the stabilisation of the structure by three TMA linkers may provide a lower steric hindrance effect, thereby enhancing the transformation of the reactants and accelerate product diffusion. The three-coordinated Cu sites with more *σ*-holes exhibit higher activity and higher binding affinities for epoxide molecules because of the smaller band gap compared with four-, two- and one-coordinated Cu sites. Second, three-coordinated Cu sites with high activity accelerate electron transfer and bonding. The relevant partial density of states (PDOS) of HKUST-1 has a very low tail at the conduction band minimum, whereas CASFZU-1 has an obviously left-shifted peak. The unsaturated state of the Cu increased the state density of CASFZU-1 at the edge of the conduction band (Supplementary Figure 47). These results indicated that the 3*d e*_g_ states of the coordinatively unsaturated Cu atoms were filled with more electrons in CASFZU-1 than in bulk HKUST-1, unambiguously indicating that under-coordinated Cu atoms are more reactive^11^. Third, a reliable Lewis acid-based catalytic mechanism can explain the enhanced catalytic performance of CASFZU-1 from the free energy viewpoint. Taking propylene oxide as an example, the DFT was applied to calculate the relative free energy for the intermediates, transition states and products in the cycloaddition promoted by catalyst and co-catalyst, respectively (Fig. 4f and Supplementary Figures 48–52). Without the co-catalyst, the energy barrier for the rate-limiting step was as high as 62.52 kcal mol^−1^ and almost no reaction occurred (Supplementary Figure 50). After adding co-catalyst to the catalytic system, cycloaddition was carried out in multiple reaction steps. The transition state and corresponding energy barrier of the two main steps, i.e., ring opening and intramolecular cyclisation, are shown in Fig. 4f. The epoxide ring-opening step (TS1) with an energy barrier of 26.23 kcal mol^−1^ became the rate-limiting step with the highest energy barrier for cycloaddition promoted by HKUST-1 (Fig. 4f). The energy barrier of this step for CASFZU-1 with three-coordinated Cu sites was only 13.89 kcal mol^−1^, which was smaller than that for HKUST-1, suggesting a key role of the three-coordinated Cu sites in this reaction. Furthermore, the overall energy barrier for CASFZU-1 (23.20 kcal mol^−1^) was markedly reduced compared to that for HKUST-1 (28.95 kcal mol^−1^), thus providing CASFZU-1 with enormous catalytic acceleration.

### Mechanical stability and reconstruction

**Fig. 5:**
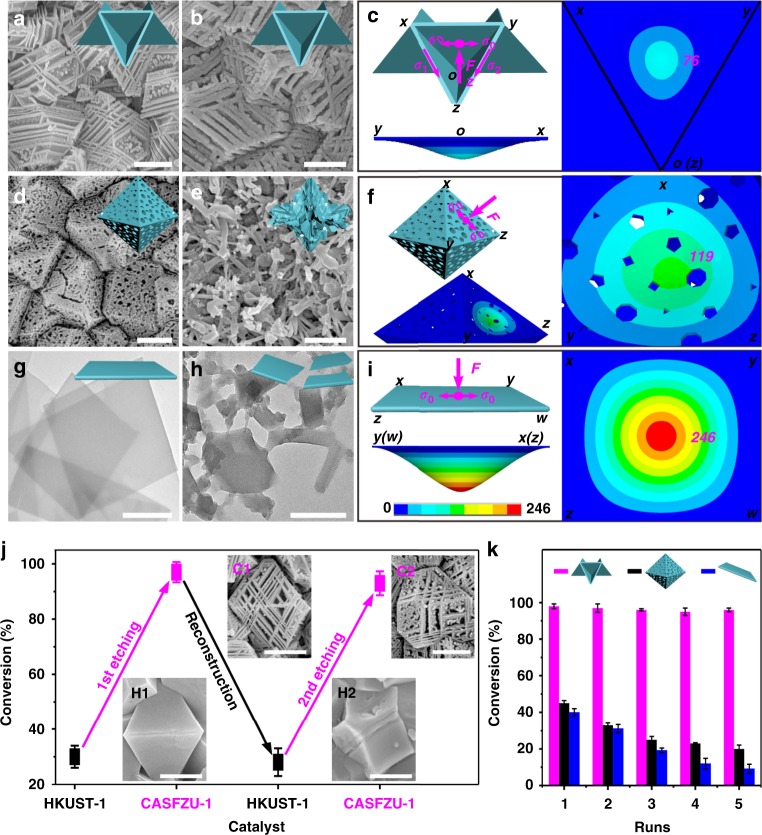
Mechanical stability and reconstruction of CASFZU-1. **a**, **b** Scanning electron microscopy (SEM) images of CASFZU-1 before and after five runs of catalysed reaction. **c** The model of CASFZU-1 and its simulated cloud pictures of the displacement distribution profiles under an external stress, *F*. **d**, **e** SEM images of random-etching MOFs before and after five runs of catalysed reaction. **f** The model of random-etching MOFs and its simulated cloud picture of the displacement distribution profiles under an external stress, *F*. **g**, **h** Transmission electron microscopy (TEM) images of a monodispersed HKUST-1 nanosheet before and after five runs of catalysed reaction. **i** The model of the HKUST-1 nanosheets and its simulated cloud picture of the displacement distribution profiles under external stress, *F*. **j** Recoverability of CASFZU-1 and its catalytic activity after reconstruction. The transformation of CASFZU-1 into HKUST-1 via reaction with 5 mM H_3_BTC and all of the CASFZU-1 (C1, C2) had higher activities than HKUST-1 (H1, H2). **k** Recyclability characterisation of CASFZU-1 (red), random-etching MOFs (black) and HKUST-1 nanosheets (blue) for five runs of catalysed CO_2_ for cycloaddition reaction with 2-ethyloxirane. Scale bars represent 500 nm for **a**, **b**, **d**, **e**, **g**, **h** and **j**. Error bars represent the standard deviation of three replicate samples

In addition, because the stacked-sheet structure enables distribution of the stress or strain developed during the chemical reaction, the CASFZU-1 catalyst showed better catalytic stability than HKUST-1 nanosheets and random-etching MOFs. The morphology and framework of CASFZU-1 were well retained after five successive catalytic cycles, indicating the long-term stability of the catalyst (Fig. 5a–c and Supplementary Figures 53 and 54). However, as significant breakage and agglomeration of fragments occurred, the catalytic activities of random-etching MOFs and monodispersed HKUST-1 nanosheets were only 44.4% and 23.0%, respectively (Fig. 5d–I, k, and Supplementary Figures 55 and 56). The greatly enhanced structural stability of CASFZU-1 was attributed to the regular triangular shape formed by the intersection of the nanosheets at a perfect 60° angle. The stress generated by adsorption of molecules during a catalytic process can be mitigated by this unique mechanical structure. When one side of the triangle suffers external stress, the other two sides offer counteracting stresses guaranteeing minimal deformation of the nanosheets under external stress, consistent with our simulated results. Under the same external stress, the displacements of CASFZU-1, random-etching MOFs and HKUST-1 nanosheets were 76, 119 and 246, respectively (Fig. 5c, f, i).

Unlike other MOF nanosheets, we can alter the catalytic performance of these MOFs by varying their structures between those of HKUST-1 and CASFZU-1. We filled in and smoothed most of the visible interlayer of the CASFZU-1 by treatment with a solution containing a certain concentration of organic ligands at room temperature. This increased the intensity of the powder X-ray diffraction (XRD) peaks attributed to HKUST-1 (Supplementary Figures 57 and 58). In comparison with HKUST-1 (yields of 30.0% ± 1.6% for H1, 28.0% ± 2.7% for H2, 25.0% ± 4.0% for H3 and 21.0% ± 2.5% for H4), the regenerated CASFZU-1 (yields of 98.0% ± 1.3% for C1, 93.0% ± 2.3% for C2, 85.0% ± 2.8% for C3 and 70.0% ± 3.8% for C4) clearly showed better performance with regard to converting CO_2_ into cyclic carbonates (Fig. 5j and Supplementary Figures 59 and 60). A thin CASFZU-1 film attached to a flexible polymer membrane provided good reusability and convenient transferability, which enabled the framework to maintain its shape and catalytic activity even after five successive catalytic cycles (Fig. 5k and Supplementary Figure 61). The performance and stability were far superior to those of the random-etching MOFs and HKUST-1 nanosheets.

## Discussion

In summary, controllable and precise etching of the organic ligands on the surface of MOF catalysts enabled the construction of a stacked-nanosheet superstructure, the planes of which intersected at a perfect 60° angle. The highly active three-coordinated Cu sites were effective for important cycloaddition reaction of CO_2_, inducing activity markedly exceeding the maximum values of previous reported catalysts, especially under industrially favoured conditions of non-energy consumption. The CASFZU-1 heterogeneous catalyst could maintain undiminished activity in marked contrast to the nanosheet catalyst that typically suffers from fragmentation and aggregation to form large unreactive species that prevent continuity of the reaction. Our systematic studies revealed that three-coordinated Cu sites played a significant role in achieving high efficiency, and the special geometric superstructure determined the robust characteristics of the CASFZU-1 catalyst. In addition, the recoverability of the catalyst demonstrated a strategy for the design and development of heterogeneous catalysts that are highly efficient toward chemical transformations and have long life cycles. It is important to further explore how this synthesis route and advanced structure can be used to tune the reactivity of diverse heterogeneous systems.

## Methods

### Preparation of bulk HKUST-1 and CASFZU-1

Equal volumes of 4 mM copper nitrate solution and 1.4 mM aminoethanol solution were rapidly mixed and aged at 60 °C for 1 h. Filtering 60 mL of the mixture solution through a nylon 66 microporous membrane left a thin light blue film on the membrane. The obtained film was reacted with 15 mL of 5 mM trimesic acid ethanol/water (1:1 v/v) solution at room temperature. A pure HKUST-1 thin film was formed typically after 1 h. To obtain the CASFZU-1 thin film, the membrane holding the pure HKUST-1 thin film was dried at 60 °C for 10 min to remove residual solvent and then stored at RH 25% for 1 month.

### Reconstruction of CASFZU-1

A used CASFZU-1 thin film was reacted with 10 mL of 5 mM H_3_BTC ethanol/water (1:1 v/v) solution at room temperature. A pure HKUST-1 thin film was formed typically after 30 min. The membrane holding the pure HKUST-1 thin film was then dried at 60 °C for 10 min to remove residual solvent and stored at RH 25% for 1 month to produce CASFZU-1.

### Two-step preparation of HKUST-1 nanosheets

*Preparation of Cu*_*2*_*O nanocubes*^32^: Typically, cupric chloride (CuCl_2_; 0.10 mmol) and polyvinylpyrrolidone (PVP; 0.10 g) were dissolved in 40.0 mL of deionised water, followed by dropwise addition (1 drop per s) of 2.5 mL of 0.2 M NaOH solution; the mixture was stirred magnetically for 5 min. Then, 2.5 mL of 0.10 M ascorbic acid solution was added dropwise (1 drop per 3 s) and the solution was further stirred for 5 min. Finally, the cubic Cu_2_O nanoparticles were suspended in 10 mL of ethanol (Cu concentration: *ca*. 10 mM) for future investigation.

*Preparation of HKUST-1 nanosheets*^33^: Typically, 0.4 g of PVP was dissolved in 60 mL of water and then 0.2 g of TMA dissolved in 4 mL of ethanol was added. The white mixture was stirred for 5 min before the addition of 10 mL of Cu_2_O ethanolic suspension. The mixture became transparent within 1 min. After stirring for 2 h, the solution became a pale blue turbid suspension, which indicated the formation of HKUST-1 nanosheets. The mixture was stirred for 16 h at room temperature. The solid product was then rinsed twice with ethanol/water and vacuum-dried at room temperature before further use.

### Carbon fixation catalysed by HKUST-1 and CASFZU-1

The catalyst was vacuum-dried at 150 °C overnight before use. The catalytic reaction was typically conducted in a three-necked flask containing epoxide (25 mmol) and purged with a flow of CO_2_ at a pressure of 1 atm in a solvent-free environment at room temperature. The added catalyst was 0.125 mol% per Cu paddlewheel unit of MOF (5.5 mg for HKUST-1, random-etching MOFs, collapsed MOFs and CASFZU-1) with a co-catalyst of tetra-*n*-tetrabutylammonium bromide (TBAB, 0.65 g, 10 mol%). The reaction was allowed to proceed for 36 h (22 h for 2-methyloxirane). The products were analysed by gas chromatography (GC; model 6820; Agilent Technologies, Santa Clara, CA, USA) equipped with a flame-ionisation detector and further identified by comparing GC retention times and mass spectra with those of authentic samples. The recovered catalyst was collected by centrifugation, then vacuum-dried at 120 °C overnight.

### Characterisation

The products were characterised by XRD (model D/MAX2500; Rigaku, Tokyo, Japan) with Cu–Kα radiation at a scan rate of 3°min^−1^. The in situ FT-IR spectra were measured using a Nicolet iS 50 instrument (Thermo Fisher Scientific, Waltham, MA, USA) from 550 to 4000 cm^−1^ with resolution of 8 cm^−1^, using 64 scans by the KBr tableting method. Before measurements, samples were degassed *in vacuo* (10^−5^ Torr) at 250 °C for 1 h. The surface area and pore diameter were determined with a physisorption analyser (model ASAP 2020 M; Micromeritics, Norcross, GA, USA) at −196 °C. Before measurements, samples were degassed *in vacuo* at 180 °C for at least 8 h. The Brunner–Emmet–Teller (BET) method was used to calculate the specific surface areas (SBET) using adsorption data at *P*/*P*_*0*_ of 0.05–0.30. The pore size distributions (PSDs) were derived from the adsorption branches of the isotherms using the Barrett–Joyner–Halenda (BJH) model. The total pore volume (*V*_t_) was estimated from the adsorbed amount at *P*/*P*_*0*_ of 0.995. We conducted gas sorption analyses using a Micromeritics ASAP 2020 with extra-high purity gases at 298 K. We degassed the samples *in vacuo* at 180 °C for at least 8 h prior to the measurements. Thermogravimetric analysis (TGA) was carried out using a Pyris 1 TGA (PerkinElmer, Waltham, MA, USA) with a nitrogen flow rate of 10 mL min^−1^. The morphologies were characterised by scanning electron microscopy (SEM; S-4800; Hitachi, Tokyo, Japan) equipped for EDX analysis. The TEM images were obtained using Hitachi H-800 and JEOL JEM-2010 (JEOL, Tokyo, Japan) instruments at an acceleration voltage of 200 kV.

### X-ray absorption data collection, analysis and modelling

Cu K-edge X-ray absorption spectra were acquired under ambient conditions in transition mode at beamline 1W1B of Beijing Synchrotron Radiation Facility (BSRF), using a Si (111) double-crystal monochromator. The storage ring of BSRF was operated at 2.5 GeV with a maximum current of 250 mA in decay mode. While the energy was calibrated using Cu foil, both the incident and transmitted X-ray intensities were monitored using standard ion chambers. The XAFS raw data were background-subtracted, normalised and Fourier-transformed by standard procedures with the ATHENA program^34^ Least-squares curve fitting analysis of the EXAFS *χ*(*k*) data was carried out using the ARTEMIS program^34,35^, based on the standard EXAFS equation consisting of single and multiple-scattering expansions. The scattering amplitudes and phase shifts for all paths, as well as the photoelectron mean free path, were theoretically calculated by ab initio code FEFF9.0^36^. The Hedin–Lundqvist self-energy and simple final-state rule core hole were used, via which an account of charge transfers between the absorber Cu and surrounding atoms and accurate determination of Fermi level enabled us to perform the fit with a single energy shift, *ΔE*_0_. The passive electron reduction factor *S*_0_^2^ was determined in the fit of CuO standard and fixed in the rest of the EXAFS models. All the EXAFS data for HKUST-1 and CASFZU-1 samples before and after dehydration were modelled with the hydrated [Cu_2_C_4_O_8_](H_2_O)_2_ cage (see Supplementary Figure 34a) to allow optimisation of the occupancy of absorbed water molecules during the fitting procedure, consistent with the strategy reported in ref. ^26^. Specifically, six significant paths were used to interpret the FT-transformed EXAFS signal in the 1.0–3.0 Å range; they included the Cu-O1, Cu-C, Cu-Cu and Cu-O2 single-scattering (SS) paths, as well as the Cu-O1-C1 double-scattering path from the carboxylate ligand and the Cu-Ow SS path from water molecules, as shown schematically in Supplementary Figure 29. All fits were performed in the *R* space with *k*-weight of 3. The EXAFS *R*-factor (*R*_f_) that measures the percentage misfit of the theory to the data was used to evaluate the goodness of fit. The best-fit results are shown in Fig. 3b, c and Supplementary Figures 30–32, with the fitting parameter values listed in Supplementary Table 3.

Quantitative XANES fitting was carried out with the MXAN code in the framework of the full multiple-scattering scheme using Muffin-tin approximation for the potential^27,28^. The energy-dependent exchange-correlation potential was calculated in the real Hedin–Lundqvist scheme. Inelastic processes were taken into account by convolution with a broadening Lorentzian function having an energy-dependent width of the form Γ(E) = Γ_c_ + Γ_mfp(_E), in which the constant part Γ_c_ takes care of both the core-hole lifetime and the experimental resolution, while the energy-dependent term represents intrinsic and extrinsic inelastic processes. The minimisation of the XANES spectra for HKUST-1 and CASFZU-1 samples before and after dehydration was carried out starting from all plausible coordination structures for Cu cations, as shown schematically in Supplementary Figure 34. The fitting quality was evaluated using the square residue function (*R*_sq_), where a statistical weight of 1 and a constant experimental error of 1.2% were used, with the best-fit results shown in Fig. 3e–g and Supplementary Figures 34–37; the structural parameters are shown in Supplementary Table 4.

### Theoretical models and DFT calculations

All of the DFT calculations in this work were carried out using the B3PW91 density function^37^ in the GAUSSIAN 09 software package^38^. The Los Alamos double-zeta-type LANL2DZ and effective core potential (ECP) basis sets were used for the Cu and Br atoms, while the 6–311 + G(d, p) split valence basis set was used for the other atoms for geometric optimisation and frequency calculations. Intrinsic reaction coordinate (IRC) calculations were performed at the B3PW91/6-31 + G(d)/LANL2DZ level to confirm that a given transition state connects a particular pair of consecutive minima. Based on the optimised structures, the zero-point-corrected Gibbs free energy at 298 K was calculated at the same computational level to obtain the potential energy surface profiles of the cycloaddition reaction. The HOMO, LUMO and electronic static potential (ESP) were generated by the GaussView program^39^, while the total density of states (TDOS) and PDOS were obtained by Multiwfn 3.3.7 software^40^.

### Simulation of strain profiles

The cloud picture of the displacement distribution profiles after processing was simulated using ANSYS engineering software. The model used to simulate the cloud picture of the displacement distribution consisted of three plates made of two-dimensional HKUST-1 nanosheets of dimensions 1000 × 1000 × 10 nm^3^; the intersecting lines of the three plates converged at perfect 60° angles and the facets of all adjacent plates formed a dihedral angle of 70.529°. We used a model of (random-etching MOFs) octahedral HKUST-1 of dimensions 1000 nm × 1000 nm (bottom square) × 750 nm (height for the rectangular pyramid) to simulate the cloud picture of the displacement distribution. The material properties were as follows: density, 1.22 × 10^−6^ ng nm^−3^; elastic modulus, 5 GPa^41^; and Poisson’s ratio, 0.3^42^. The octahedron and plates were constrained as follows. The displacements of the most external nodes of the constrained edges were zero in three directions. Simulated loads were applied to the plates as 20-nN forces at the centres of 10 × 10 nm^2^ regions^15^.

## Supplementary information

Supplementary Information

## Data Availability

The data that support the findings of this study are available from the corresponding author upon request.
