# Supplementary material for: Coordination mode engineering in stacked-nanosheet metal–organic frameworks to enhance catalytic reactivity and structural robustness
Source: Nat Commun. 2019 Jun 25;10:2779. doi: 10.1038/s41467-019-10547-9 (PMC6592929; doi:10.1038/s41467-019-10547-9)
Supplement: Supplementary file 1 — Supplementary Information [file 41467_2019_10547_MOESM1_ESM.pdf]

Coordination mode engineering in stacked-nanosheet metal–organic frameworks to enhance catalytic reactivity and structural robustness

Supplementary Information

Huang et al.

Supplementary Methods.

Experimental materials. All chemicals used were at least of analytical grade. Copper nitrate trihydrate ($Cu(NO_3)_2$•$3H_2O$), 2-aminoethanol (NH_2-CH_2CH_2OH), 1,3,5-benzenetricarboxylic acid (trimesic acid, H_3BTC), terephthalic acid (H_2BDC) were purchased from Aladdin (Shanghai, China). 2-methyloxirane, 2-ethyloxirane, 2-(chloromethyl)oxirane, 2-(bromomethyl)oxirane, 2-octyloxirane and 1,2-epoxydodecane were purchased from Sigma Aldrich. The supports were Nylon 66 microporous membranes (Jin teng) with an average pore size of *ca* 220 nm and porosity of 50%. Ultrapure water (18.2 MΩ) produced by a Millipore direct-Q system (Millipore) was used throughout the experiments. CO_2 (>99.95%) was provided by Beijing Analysis Instrument Factory.

Preparation of MOF-5(Zn) cubic crystal: MOF-5 crystals were synthesized in *N*,*N*-Dimethylformamide (DMF) at room temperature as previous report.[1] Typically, terephthalic acid (0.507 g) and triethylamine (0.85 mL) were dissolved in 40 mL DMF. Following zinc acetate dihydrate (1.699 g) was dissolved in 50 mL DMF and was dropwise added into the above solution under magnetic stirring at room temperature for 2.5 h. The precipitate was collected by centrifugation and then washed several times with DMF and $CHCl_3$. Finally, the material was dried in vacuum at 100 ℃ for a night.

Preparation of bulk-type CuBDC crystal: CuBDC crystals were synthesized following the hydrothermal synthesis described by Carson et al.[2] Typically, 1.053 g of copper nitrate trihydrate and 724 mg of 1,4-benzenedicarboxylic acid and 87 mL *N*,*N*-dimethylformamide (DMF) were mixed in a 250 mL roundbottom flask and refluxed at 100 ℃ during 24 h. The resulting powder was collected by centrifugation and the solid was consecutively washed 3 times with DMF (20 mL each step) followed by 3 times washing with $CHCl_3$ (20 mL each step). Finally, the material was dried in vacuum at 100 ℃ for a night.

Preparation of ZiF-8 crystal: ZiF-8 crystals were synthesized in methanol at room temperature as previous report.[3] $Zn(CH_3COO)_2$ (175 mg) was dissolved into methanol (20 mL) to form solution A. 2-methylimidazole (263 mg) was dissolved in methanol (20 mL) to generate clear solution B. Then, both of A and B were mixed together, and stirred for 5 min. The solution was aged at room temperature for 24 hours. After that, white powders were precipitated. The powders were washed very carefully with methanol for 5 times and dried in vacuum at 100 ℃ for a night.

Preparation of MIL-110(Al) crystal: MIL-110 crystals were hydrothermally synthesized in deionized water as previous report.[4] Typically, aluminium nitrate nonahydrate (0.6659 g, 1.8 mmol), trimethyl 1,3,5-benzenetricarboxylate (0.3025 g mg, 1.2 mmol), concentrated nitric acid (1 ml, 4.0 mmol) and deionized water (5 ml, 277.8 mmol). The MIL-110 phase is obtained in very acidic conditions (pH $\approx$ 0) by adding concentrated nitric acid. The starting mixture was placed in a Teflon-lined stainless steel autoclave and heated to 210 ℃ for 72 h. The resulting powdered pale yellow product was filtered off, washed with deionized water and dried in vacuum at 100 ℃ for a night.

Preparation of UiO-66(Zr) crystal: UiO-66 octahedrons were prepared through a solvothermal route as previous report.[5] In a typical synthetic process, $ZrCl_4$ (40.0 mg, 0.172 $\times 10^{-3}$ M) and 1,4-benzenedicarboxylic acid (28.5 mg, 0.172 $\times 10^{-3}$ M) were dissolved in 20.0 mL *N*,*N*-dimethylformamide (DMF) by using ultrasound for 10 min. Then 3.66 mL of acetic acid was added into the solution to tune the morphology of the product. After further 10 min ultrasonic treatment, the obtained homogeneous solution was then transferred into a 50 mL Teflon-lined stainless steel autoclave and heated to 120 ℃ for 24 h. After cooling down to room temperature, the precipitates were isolated by centrifugation and washed with DMF to remove the unreacted precursors and then soaked in methanol for three days with replacing the methanol every 24 h to exchanging the DMF solvent. Finally, the products were collected by centrifugation and dried in vacuum at 100 ℃ for overnight.

Preparation of MIL-53(Al) crystal: MIL-53 (Al) crystals were hydrothermally synthesized in deionized water as previous report.[6] Typically, the synthesis was carried out under mild hydrothermal conditions using aluminum nitrate nonahydrate, 1,4-benzenedicarboxylic acid, and deionized water. The reaction was performed in a 23 mL Teflon-lined stainless steel autoclave under autogenous pressure for three days at 220 ℃. The molar composition of the starting gels was 1 Al (1.30 g):0.5 BDC (0.288 g):80 H_2O. The resulting powdered product were isolated by centrifugation and washing with deionized water for 5 times. Finally, the products were collected by centrifugation and dried in vacuum at 100 ℃ for overnight.

Preparation of MIL-53(Cr) crystal: MIL-53 (Cr) crystals were hydrothermally synthesized in deionized water as previous report.[7] Typically, the synthesis was carried out under mild hydrothermal conditions using aluminum nitrate nonahydrate, 1,4-benzenedicarboxylic acid, and deionized water. The reaction was performed in a 100 mL Teflon-lined stainless steel autoclave under autogenous pressure for three days at 220 ℃. The molar composition of the starting gels was 1 Cr (4.00 g):1.0 BDC (1.66 g):1.0 HF:280 H_2O. The resulting pea green powdered product were isolated by centrifugation and washing with deionized water and acetone for 5 times. Finally, the products were collected by centrifugation and dried in vacuum at 100 ℃ for overnight.

Preparation of ZIF-67 particles: ZIF-67 crystals were synthesized as previous report.[8] In a typical synthesis, 2-methylimidazole (1.97 g) is dissolved in a mixed solution of 20 mL methanol and 20 mL ethanol. $Co(NO_3)_2 \cdot 6H_2O$ (1.746 g) is dissolved in another mixed solution of 20 mL methanol and 20 mL ethanol. The above two solutions are then mixed under continuous stirring for 10 s, and the final solution is kept for 20 h at room temperature. The purple precipitate is collected by centrifugation, washed in ethanol three times and dried at 100 ℃ for overnight.

Preparation of MIL-88-Fe particles: MIL-88-Fe crystals were hydrothermally synthesized as previous report.[9] Typically, $FeCl_3 \cdot 6H_2O$ (0.748 g) and 1,4-benzenedicarboxylate (0.460 g) were dissolved in 120 mL DMF. After thorough mixing, the solution was transferred into a Teflon-lined stainless steel autoclave and placed in an oven at 150 ℃ for 7 days. After cooling down to room temperature, the product was collected by centrifugation, washed with DMF and ethanol for three

times, respectively. At last, the orange powder of MIL-88-Fe was obtained after drying in a vacuum oven at 100 ℃ for 12 h.

Cyanosilylation over pristine HKUST-1 and CASFZU-1: The as-prepared samples were activated in vacuum at 120 ℃ for 12 h. Then, the degassed 6 mg pristine HKUST-1 and CASFZU-1 were transferred into a flask, respectively, and 80 uL benzaldehyde (0.79 mmol), 220 uL trimethylsilylcyanide (TMSCN, 1.65 mmol), 4 mL heptane were added. The mixture was allowed to react at 60 ℃ for 48 h with stirring and the products were analyzed by GC-MS. [Benzaldehyde (Aladdin Industrial Inc., 99.5%, GC), trimethylsilylcyanide (Aladdin Industrial Inc., 97%, AR), heptane (Aladdin Industrial Inc., 99.5%, GC).

Theoretical models and computational details. As the catalytic active site is copper, the HKUST-1 and CASFZU-1 surfaces were modelled by simple building blocks (a paddlewheel Cu_2 dimer surrounded by four coordinated formic acid molecules for HKUST-1, a paddlewheel Cu_2 dimer surrounded by three coordinated formic acid molecules for CASFZU-1, see Supplementary Figure 48) to achieve a best compromise between computational cost and accuracy of computational outcomes. Besides, the large tetrabutylammonium bromide (TBAB) was also simplified to tetramethylammonium bromide. Then, the intermediates are constructed by combining the reactants with Cu-site via adopting different orientations, and the lowest-energy one was adopted for each intermediate after initial geometric optimisation. All the geometries of the isolated reactants, products, intermediates, and transition states involved in the cycloaddition reaction have been fully optimised without any constraints via DFT calculations by using the B3PW91 density functional.[10,11] The Los Alamos double-zeta-type LANL2DZ and effective core potential (ECP) basis sets were used for the Cu and Br atoms, while the 6-311+G(d,p) split valence basis set was used for the other atoms for the geometric optimisation. Vibrational frequency calculations, from which the thermal corrections to Gibbs free energy were derived, have been performed for each optimised structure at the same level to identify the nature of all the stationary points (local minimum or first-order saddle point). The intrinsic reaction coordinate (IRC)[12] pathways have been traced at the 6-31+G(d)/LANL2DZ level in order to verify that each saddle point links two desired minima. Finally, the zero-point-corrected Gibbs free energies for the isolated reactants, products, intermediates, and transition states were calculated at 298 K on the basis of the optimised structures to obtain the potential energy surface profiles of the cycloaddition reaction. All calculations were carried out using the GAUSSIAN 09 software package.[13]

To compare the electronic structures of HKUST-1 and CASFZU-1, larger models with benzyl acid molecules as ligands are considered (see Supplementary Figure 1). Their HOMO, LUMO, and electronic static potential (ESP) were generated by the GaussView program.[14] The wave functions produced by GAUSSIAN 09 software at the B3PW91/6-311+G(d,p)/LANL2DZ level were used as inputs for Multiwfn 3.3.7 software[15] to plot the total density of states (TDOS) and partial density of states (PDOS).

To evaluate the distortion of framework for paddlewheel Cu_2 cluster with different

coordination number, four large models without water molecules (Supplementary Figure 38) and two large models with water molecules (Supplementary Figure 39) have been optimised at the B3PW91/6-31G/LANL2DZ level by using the GAUSSIAN 09 software. Herein, we mainly focus on the change of on the surfaces of HKUST-1 and CASFZU-1, and thus the interior parts of the materials, namely these atoms marked by green colour in the models were frozen in the optimization progress. All the other parts of the molecular models have been fully optimised without any constraints and the nature of local minima in the potential energy surface were characterized by means of harmonic vibrational frequencies analysis.

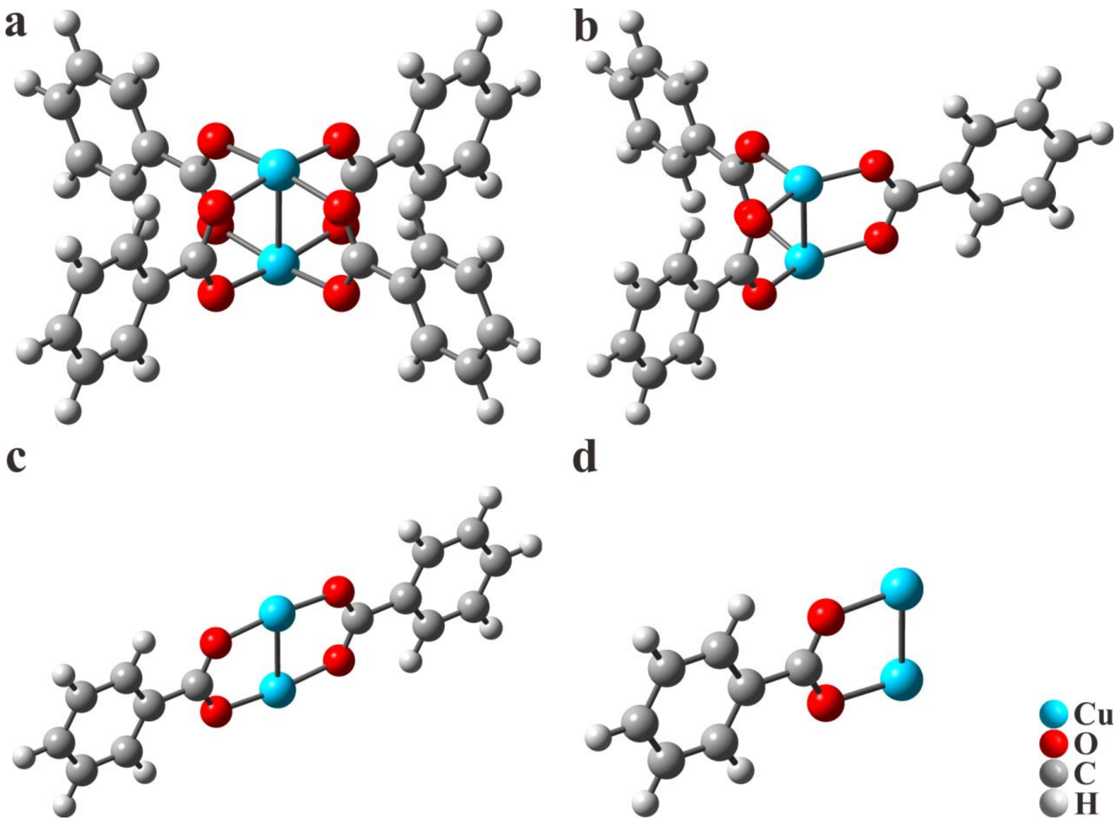

Supplementary Figure 1. Schematic models of the paddlewheel Cu_2 clusters with four different kinds of coordination numbers for the simulation of electrostatic surface potential (ESP) maps and frontier orbital energy levels and molecular orbital (MO) diagrams. Colour scheme for chemical representation: cyan for Cu, red for O, grey for C and white for H.

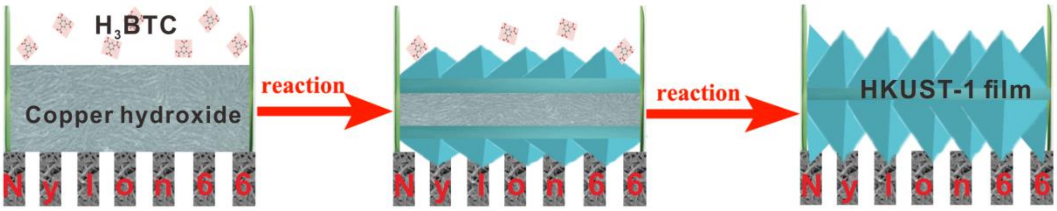

Supplementary Figure 2. Schematic diagram for construction process of MOF-HKUST-1 thin film prepared from copper–hydroxide-nanostrands on nylon 66 membrane.

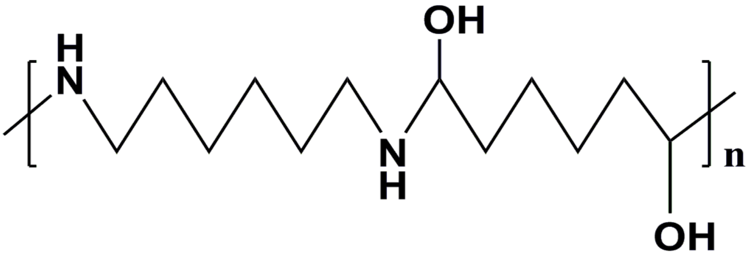

Supplementary Figure 3. Structure of Nylon 66.

It's expected that a polymer with the ability to bind copper metal would provide a favorable support for growing a HKUST-1 layer. Numerous nitrogen atoms and oxygen atoms in the molecule structure would play crucial role for their ability to form stable chelates with a wide variety of metals.

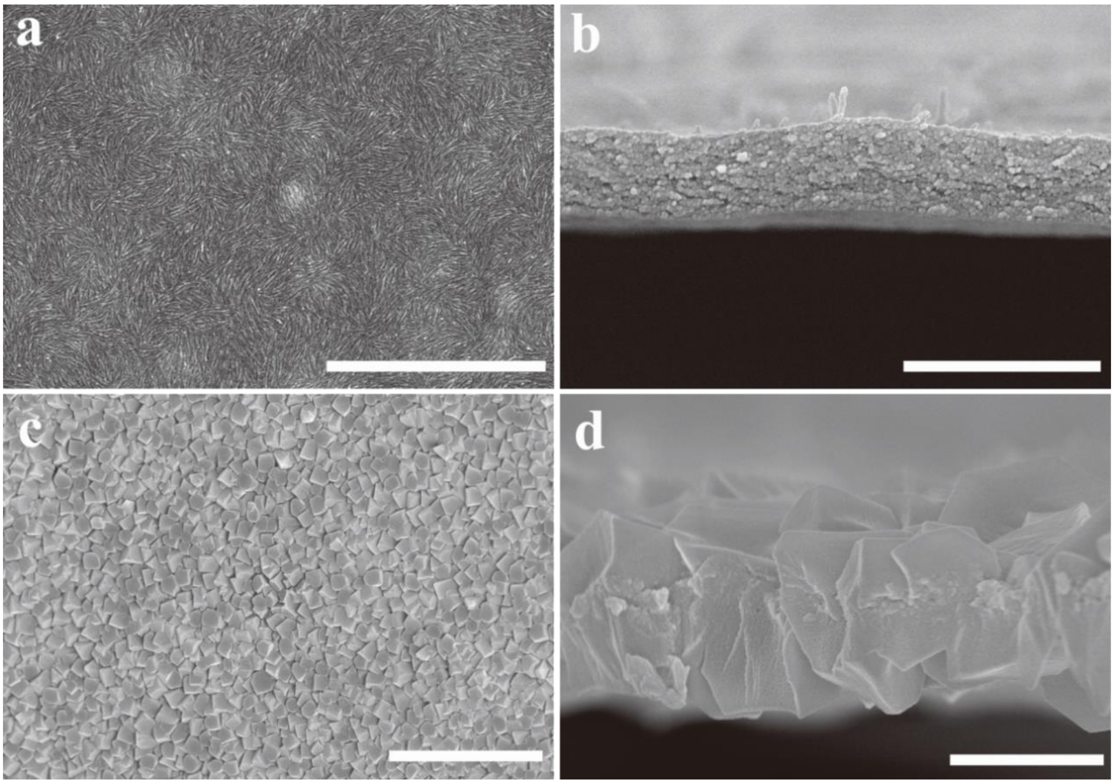

Supplementary Figure 4. (a) SEM image top view for copper-hydroxide-nanostrands and (b) SEM cross-section view for copper-hydroxide-nanostrands; (c) SEM top view for HKUST-1 thin film and (d) SEM cross-section view for HKUST-1 thin film. Scale bars, 2 µm for (a), 1 µm for (b), 20 µm for (c) and 1 µm for (d).

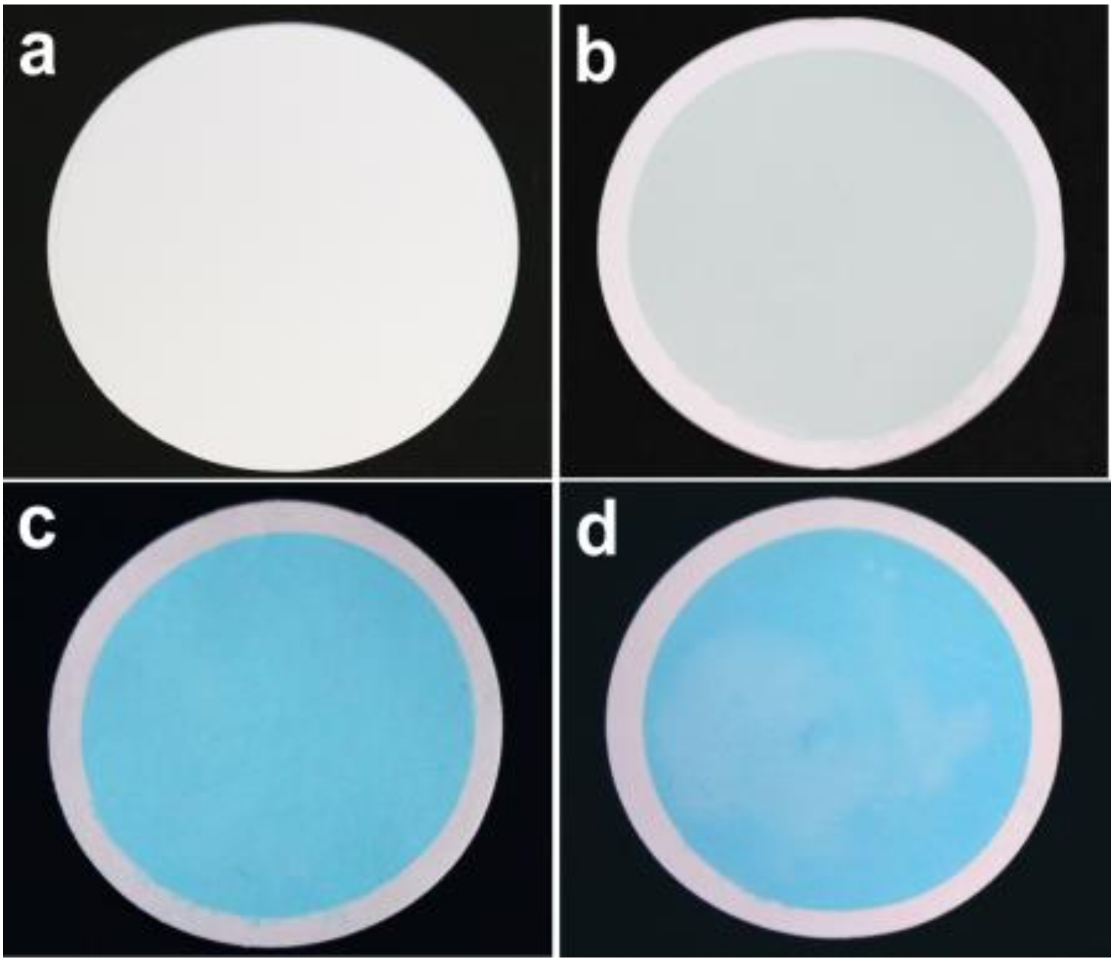

Supplementary Figure 5. (a) Photographs of bare nylon 66 membrane, (b) copper-hydroxide- nanostrands coated nylon 66 membrane, (c) pristine HKUST-1 thin film, and (d) CASFZU-1 thin film. The diameter and pore size of nylon 66 membrane are 47.0 mm and 0.22 μm, respectively.

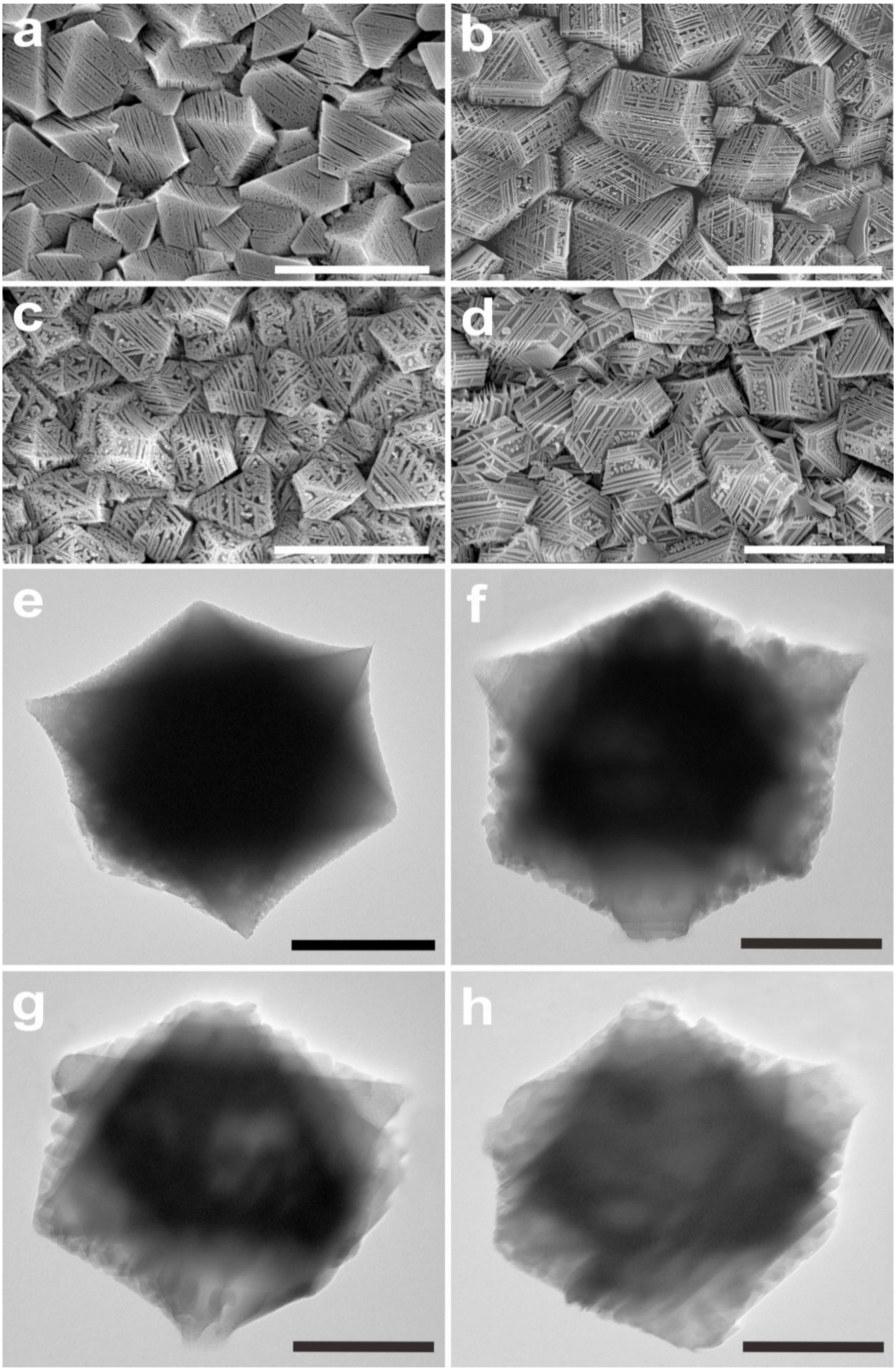

Supplementary Figure 6. (a-d) SEM and TEM images of MOF thin films prepared at (a, e) 1 week; (b, f) 2 weeks; (c, g) 3 weeks; and (d, h) 1 month. Scale bars, 2 μm for (a-d), 500 nm for (e-h).

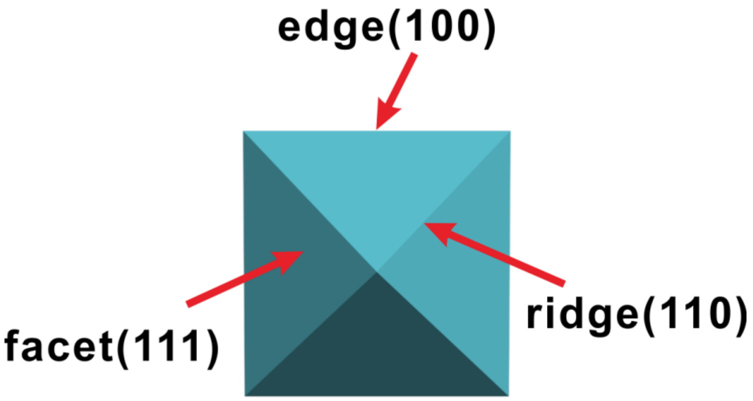

Supplementary Figure 7. Schematic illustration of a typical octahedral nanostructure for HKUST-1.

The pristine HKUST-1 consists of well-defined octahedral structures with facet (111) exposed.

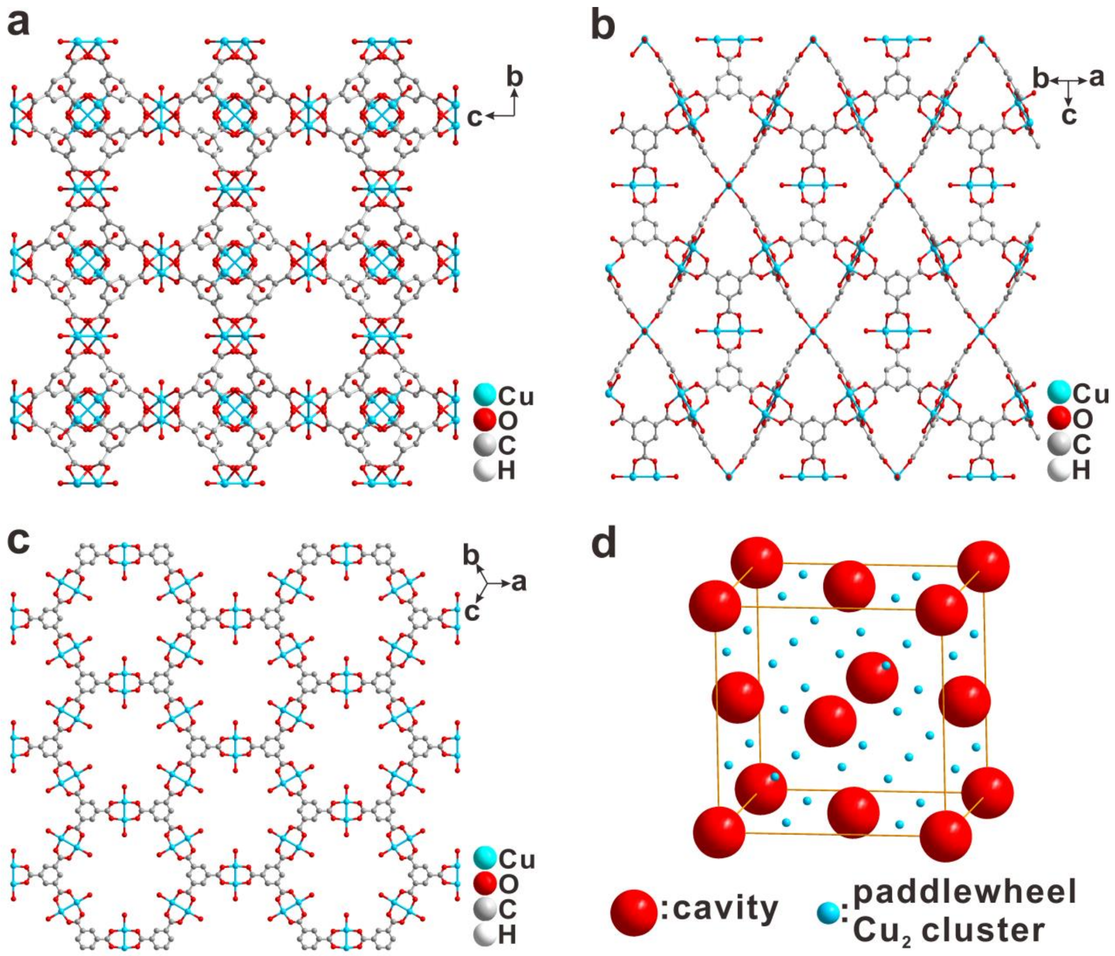

Supplementary Figure 8. (a-c) HKUST-1 viewed down the [100], [110], [111] direction, (d) Simplified model of the face-centered-cubic crystals unit cell of HKUST-1. Colour scheme for chemical representation: cyan for Cu, red for O, grey for C and white for H.

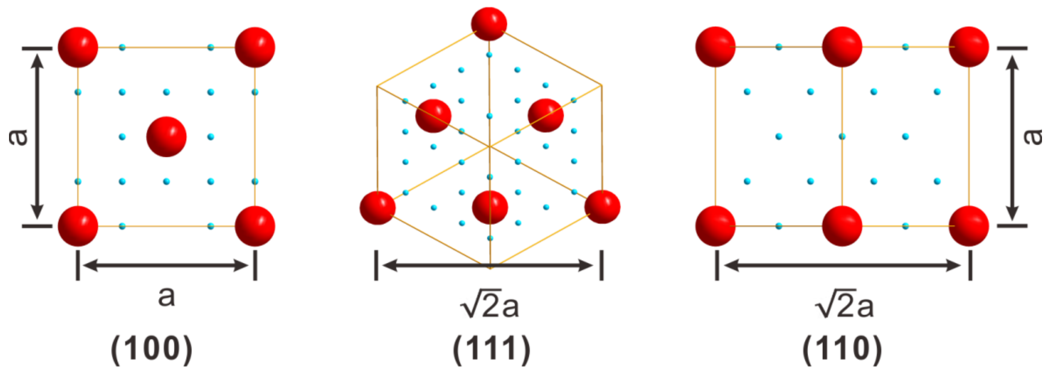

Supplementary Figure 9. Simplified model of HKUST-1 viewed down the [100], [110], [111] direction. Set the length of side of a cell is a. Colour scheme for chemical representation: red for cavity and cyan for paddlewheel Cu_2 cluster.

Supplementary Note 1. Surface energies calculation for different planes of HKUST-1 crystal.

For one-mole crystal, there are N_A atoms and at least $0.5N_A$ bonds will form among them. Take the coordination number into account, there will be $(0.5N_A*Z)$ bonds in one-mole crystal. The energy of one bond can thus be written as:

$$\varepsilon=\frac{\Delta H_S}{0.5\mathrm{N_A}*\mathrm{Z}}, \text{ where } \Delta H_S \text{ is the molar enthalpy of sublimation} \quad (1)$$

For the (100) plane in FCC crystal, atoms at the surface possess a coordination number (CN) of 8, which means that 4 bonds per atoms are broken at the surface of (100). As a result, the energy required to form one (100) surface in FCC can be given as:

$$E_{(100)} = \text{(energy of one bond)*(number of bonds broken / atom)} \quad (2)$$

$$= \varepsilon *4 = \frac{2\Delta H_S}{3\mathrm{N_A}*\mathrm{Z}} \text{ (Z=12 for FCC) … energy required per surface atom} \quad (3)$$

The surface energy γ is then defines as follows:

$$\gamma = \text{(Energy required per surface atom)*(number of surface atom/surface area)} \quad (4)$$

$$= \frac{2\Delta H_S}{3N_A*\mathrm{Z}}\left(\frac{N}{A}\right) \quad (5)$$

For (100) plane in FCC crystal:

$$\left(\frac{N}{A}\right)_{(100)} = \frac{2}{a^2} \quad (6)$$

$$\gamma_{(100)} = E_{(100)} * \left(\frac{N}{A}\right)_{(100)} = \frac{4\Delta H_S}{3\mathrm{N_A}a^2} \quad (7)$$

In a similar way, for the (111) plane in FCC crystal, 3 bonds per atoms are broken,

$$\gamma_{(111)} = \frac{2\Delta H_S}{\sqrt{3}\mathrm{N_A}a^2}. \quad (8)$$

for the (110) plane in FCC crystal, 6 bonds per atoms are broken,

$$\gamma_{(110)} = \frac{\sqrt{2}\Delta H_S}{\mathrm{N_A}a^2}. \quad (9)$$

For HKUST-1 FCC crystal: $\gamma_{(111)} < \gamma_{(100)} < \gamma_{(110)}$.

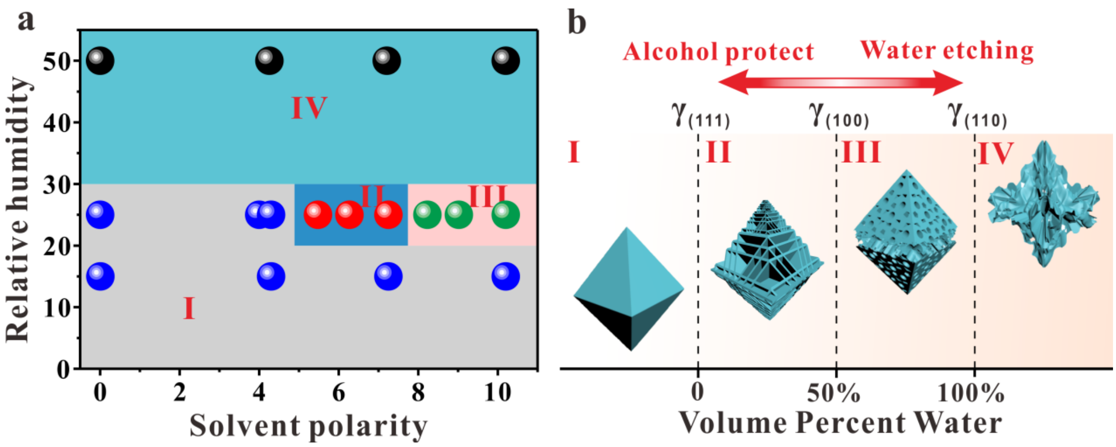

Supplementary Figure 10. (a) The relation map of etching results with relative humidity and mixed solvent polarity. The typical etching of HKUST-1 can be divided into four types (I, II, III and IV) based on the morphologies. (b) The overall etching ability of mixed solvent (H_2O and ethanol) versus surface energies for the facet (111), (100) and (110) in HKUST-1 FCC crystal under 25%RH. The volume percent of water means the volume percent of water in the pores of HKUST-1 crystals.

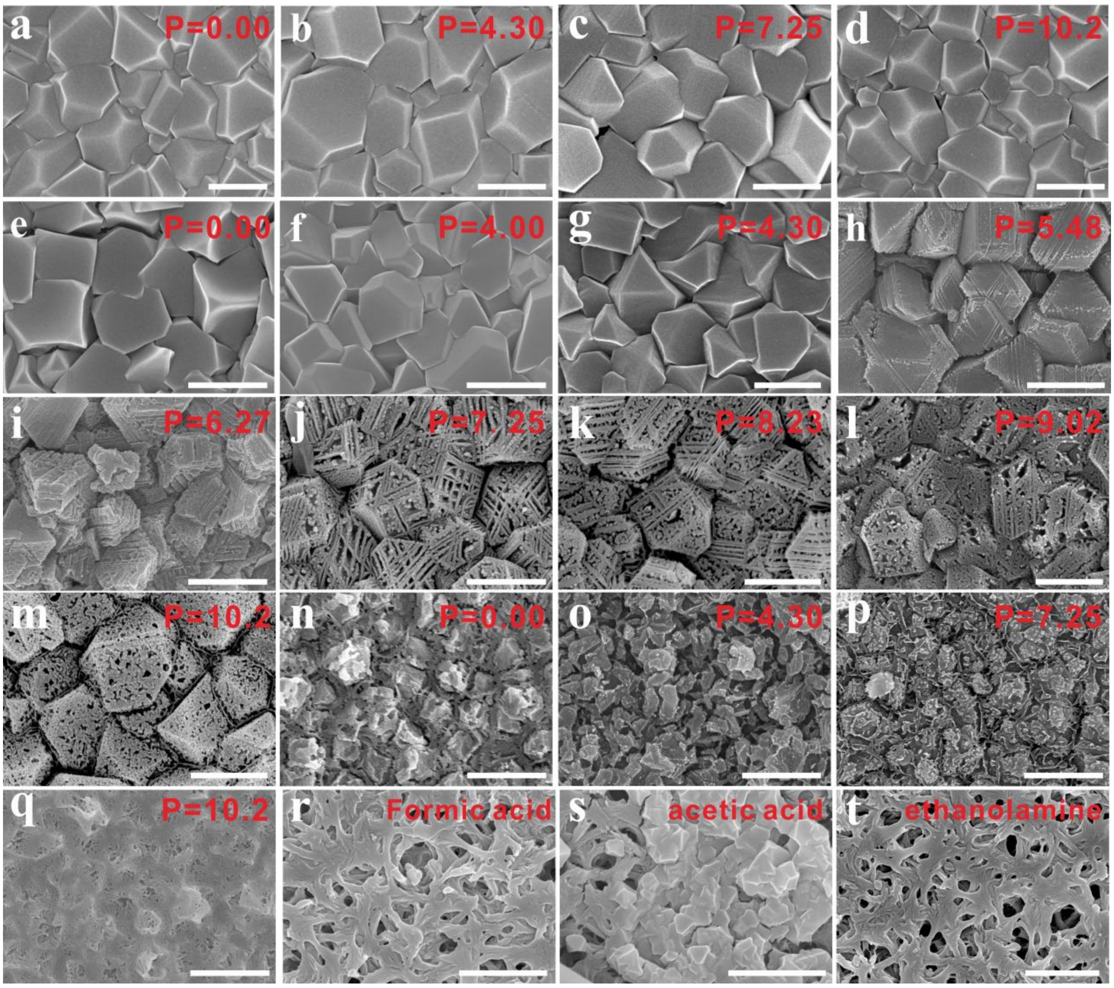

Supplementary Figure 11. SEM top view images of HKUST-1 were etching by different solvent at different RH. For a-d, RH = 15%, No solvent in a, mixed solvent (H_2O/ethanol, $V_{water}:V_{ethanol}$ = 0:1, 1:1, 1:0 for b, c, d, respectively.
For e-m, RH = 25%, No solvent in e, *n*-Propanol in f, $V_{water}:V_{ethanol}$ were 0:1, 1:4, 1:2, 1:1, 2:1, 4:1, 1:0 for g-m, respectively.
For n-q, RH = 50%, No solvent in n, $V_{water}:V_{ethanol}$ were 0:1, 1:1, 1:0 for o-q, respectively.
For r-t, RH=25%, (r) Formic acid, (s) acetic acid, (t) ethanolamine.
Scale bars, 1 μm for (a) - (q), 5 μm for (r) - (t), respectively (Inset figures were the polarity of mixed solvent).

Before etching, the membrane with pure HKUST-1 thin film was dried in vacuum at 100 ℃ for overnight, and then soaked in the mixed solvent for 5 min. Subsequently, the membrane was dried at 60 ℃ for 10 min to remove the mixed solvent on the surface of Nylon 66 membrane, and finally it was stored in relevant RH for a month. The pure organic acid (formic acid and acetic acid) and organic base (ethanolamine) can easily dissolve the HKUST-1, after etching, only small fractionlet of MOFs are left on the Nylon 66 membrane.

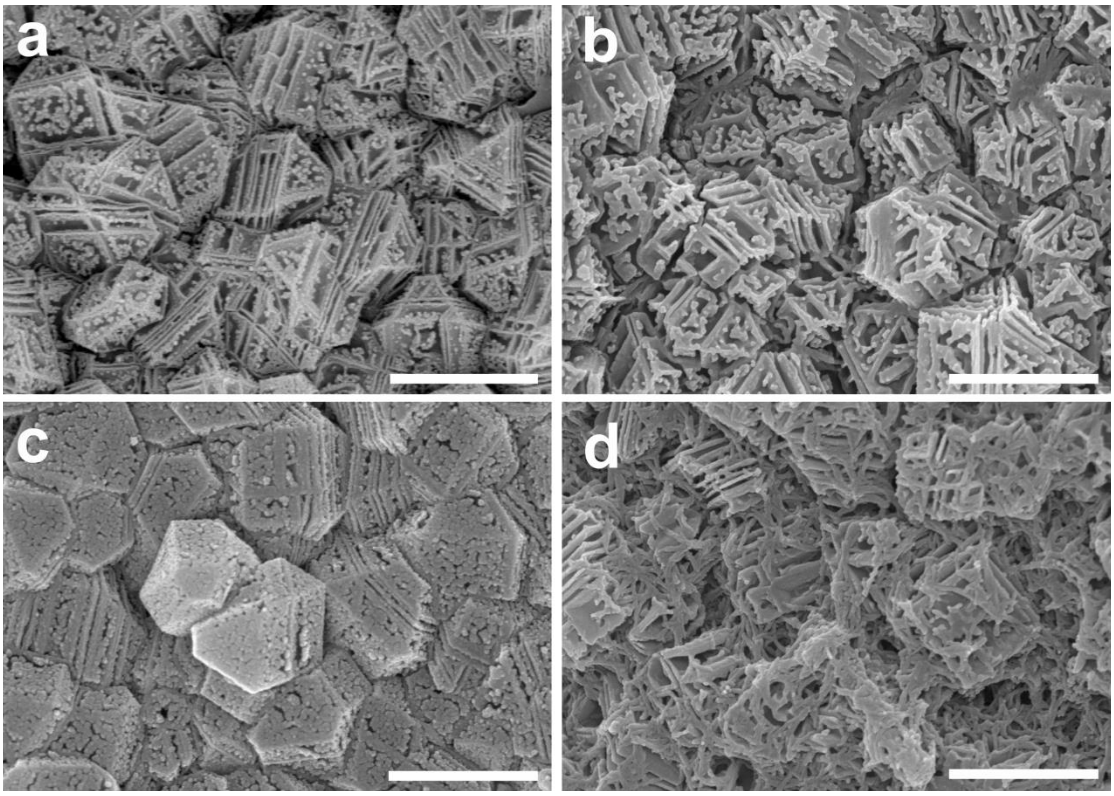

Supplementary Figure 12. SEM top view images of HKUST-1 were etching by different mixed solvent (water and alcohol, V_{water}:$V_{alcohol}$ = 1:1) at room temperature.(a) Methanol, (b) *n*-Propanol, (c) Isopropanol, (d) *n*-butanol. Scale bars, 1 μm for (a-d).

Actually, ethanol molecules in the mixed solvent are supposed to play a predominant role in stabilizing the framework with its hydrophobic alkane tails. Subsequently exploratory experiments with altered alcohols (methanol, *n*-propanol, isopropanol, *n*-butanol) were performed to test and verify the effect of alcohol. It was obviously observed that all the crystals exhibited nanosheet-like structure on the surface as the water/alcohol ratio was 1/1 at 25%RH**,** although the solvent polarity was slightly different (Supplementary table 1). Indeed, after extensive variation of synthesis conditions, we identified a synthesis window that produces CASFZU-1 via controllable etching.

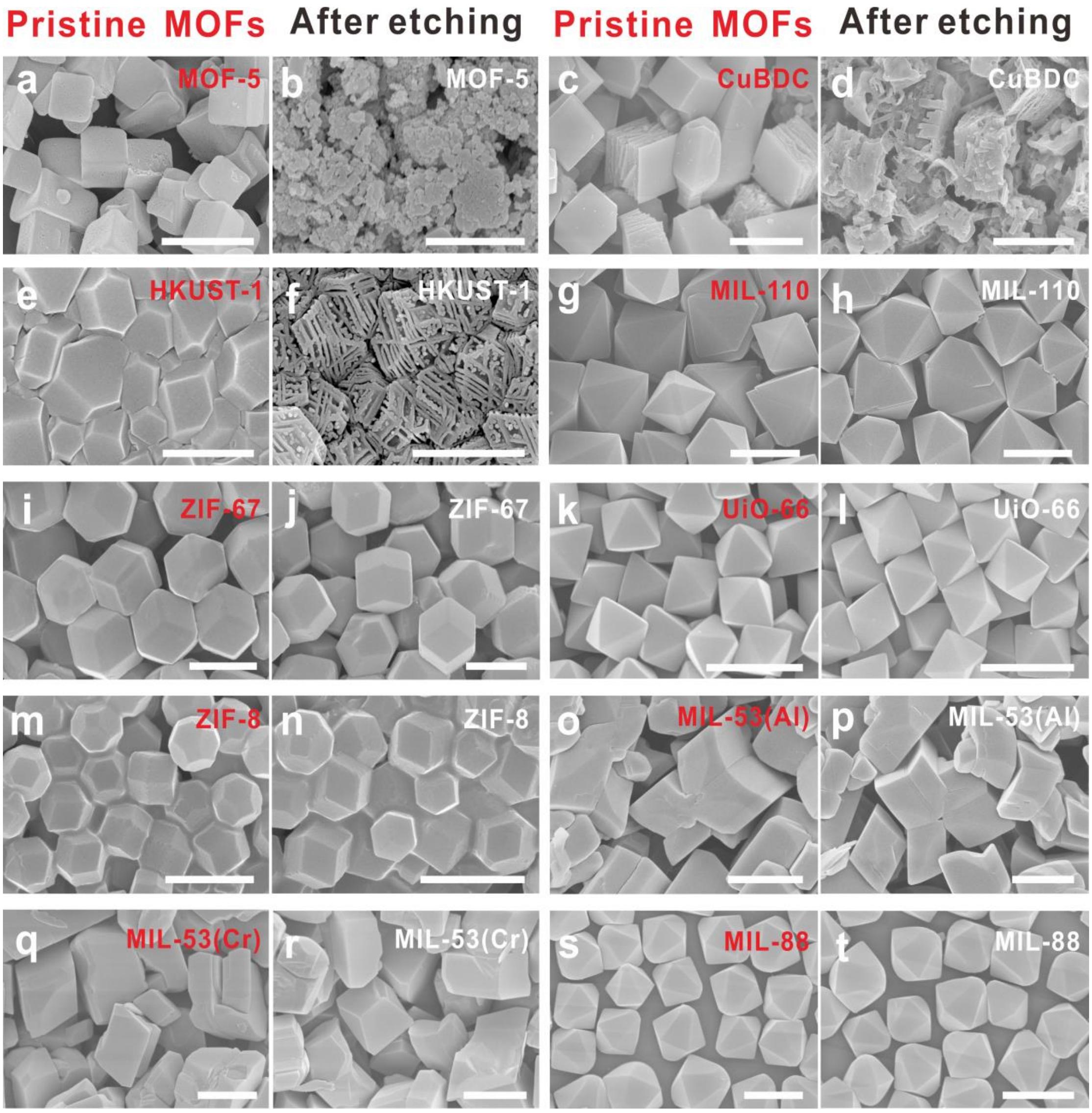

Supplementary Figure 13. SEM images of MOF-5 (Zn) (a, b), CuBDC (Cu) (c, d), HKUST-1 (Cu) (e, f), MiL-110 (Al) (g, h), ZIF-67 (Co) (i, j), UiO-66 (Zr) (k, l), ZIF-8 (Zn) (m, n), MIL-53 (Al) (o, p), MIL-53 (Cr) (q, r), MIL-88 (Fe) (s, t) before and after etched at different RH and room temperature (25 ℃) for a month. (RH 15% for MOF-5 and CuBDC, RH 25% for HKUST-1 and RH 50% for MIL-110, ZIF-67, UiO-66, UiO-66, ZIF-8, MIL-53, MIL-88). Scale bars, 2 μm for (a-b), 5 μm for (c-d), 2 μm for (e-t), respectively. In pairs of images, the pristine MOFs appear on left.

Before etching, the pure MOFs were dried in vacuum at 100 ℃ for overnight, and then soaked in the mixed solvent (H_2O/ethanol, V_{water}:$V_{ethanol}$ = 1:1) for 10 min. subsequently, the pure MOFs were dip coating on the membrane and the membrane was dried at 60 ℃ for 10 min to remove the mixed solvent on the surface of Nylon 66 membrane, and finally it was stored in relevant RH for a month.

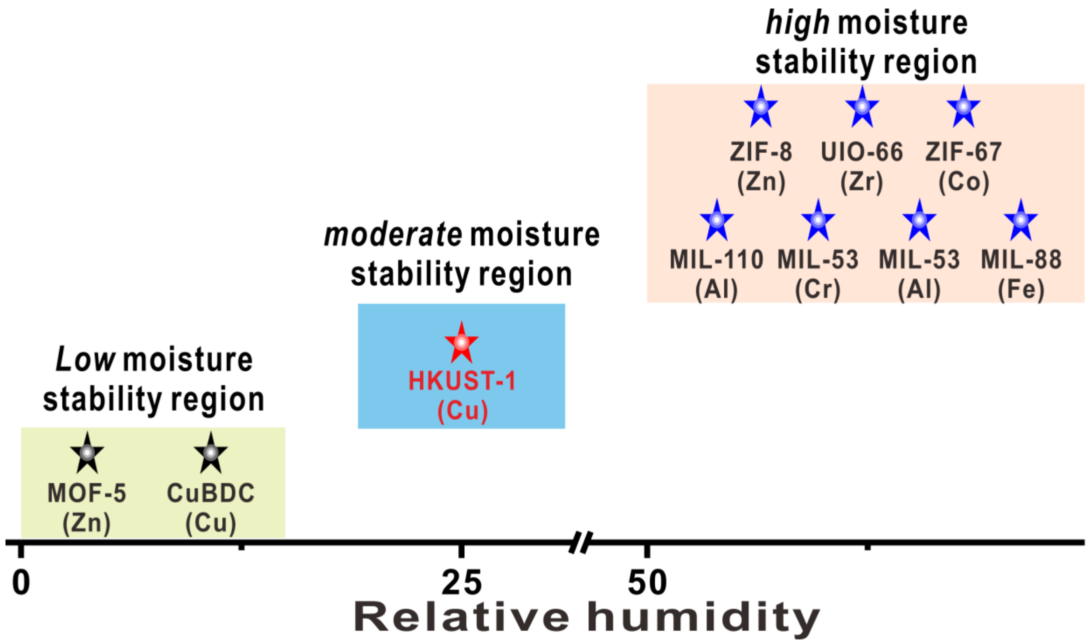

Supplementary Figure 14. Moisture stability map of the ten MOFs discussed in this paper. The position for a given MOF represents its relative structural stability by SEM images.

From the result, it can be obtained that HKUST-1 would be slightly more stable with respect to reaction with water than MOF-5 and CuBDC. ZIF-8, UIO-66, ZIF-67, ZIF-7, MIL-110, MIL-53 (Al), MIL-53 (Cr), and MIL-88 are more stable than HKUST-1. These results are consistent with the previous reports.[16] Obviously, only HKUST-1 is suitable for controllable etching experiment at moderate moisture (25% RH).

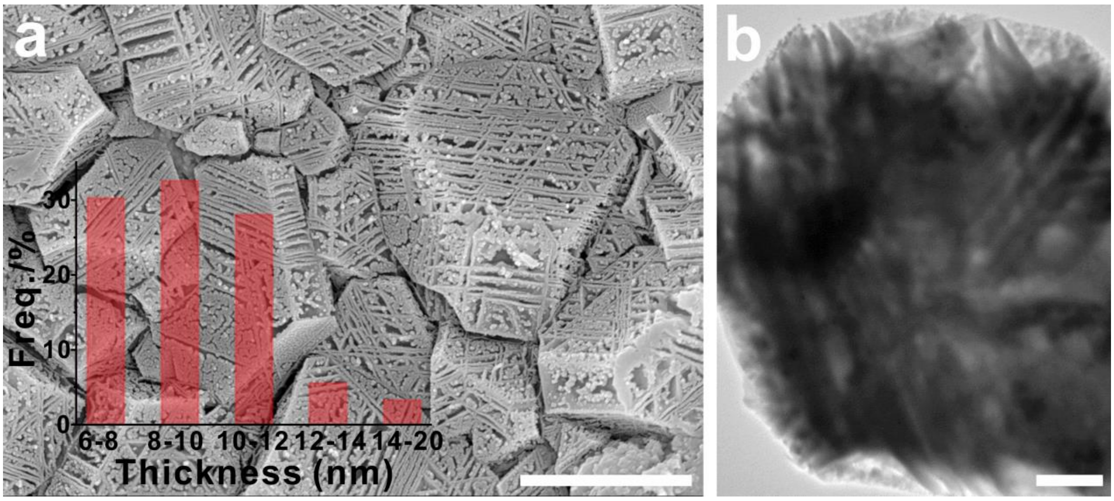

Supplementary Figure 15. (a) SEM image of CASFZU-1. The inset image is statistical analysis of the thickness of CASFZU-1 nanosheets in a. (b) TEM image of CASFZU-1. Scale bars, 1 μm for (a), 200 nm for (b).

The uniform CASFZU-1 was composed of thin MOF nanosheets with a mean diameter of ~9.25 nm, which indicates that most of the MOF nanosheets were about 2 unit cells thick along the [111] axis (HKUST-1: space group *Fm-3m*, *a* = 26.343(5) Å) .

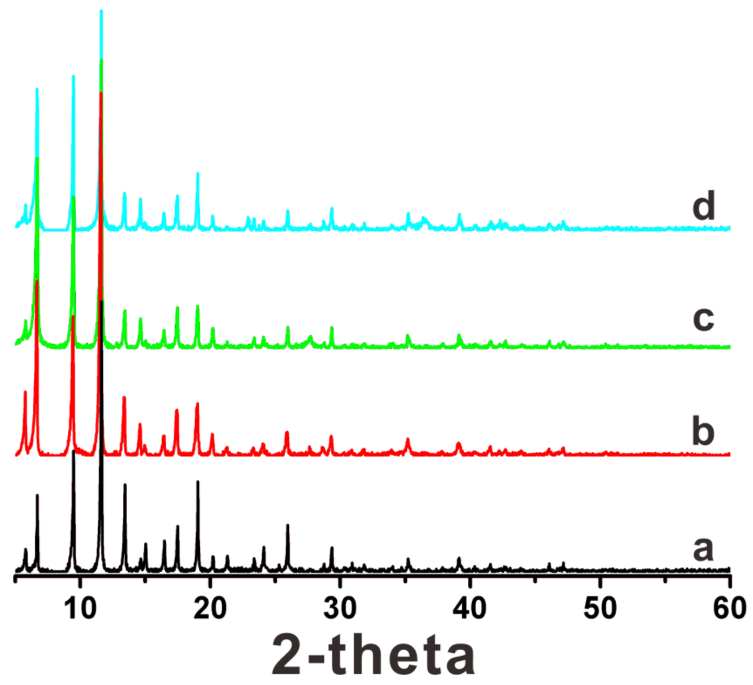

Supplementary Figure 16. XRD patterns of MOFs after storage at relevant time. Pristine MOF for (a), 1 week for (b), 3 weeks for (c) and 1 month for (d).

During the etching process, the structural integrity and crystallinity of MOF are well retained and no other crystals appeared.

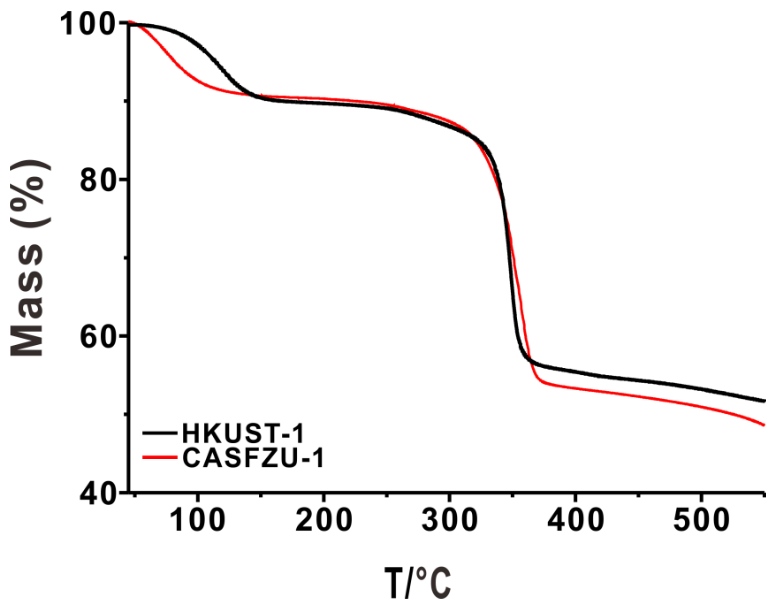

Supplementary Figure 17. TG patterns for the materials. Thermal analysis revealed that the CASFZU-1 nanosheets remain stable up to 320 ℃ which is similar to the pristine HKUST-1.

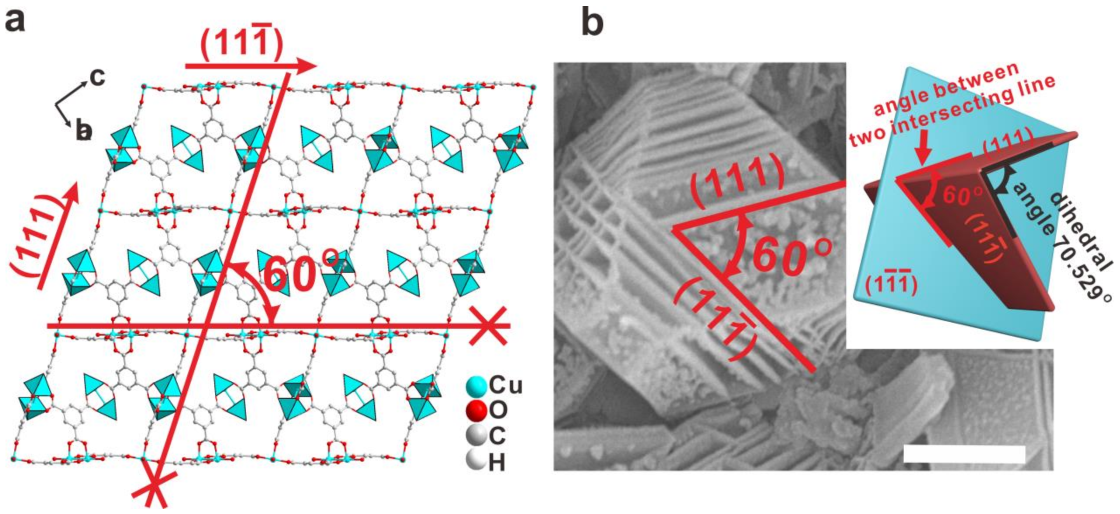

Supplementary Figure 18. (a) The HKUST-1 crystal structure along the [1$\bar{1}$0] direction. (b) SEM image of top surface [facet (1$\bar{1}\bar{1}$)] of CASFZU-1. Scale bar, 500 nm for (b).

The two intersection lines which are produced by the intersection of the facet (111) and nanosheet [The intersection of two faces (111) and (1$\bar{1}\bar{1}$) and the intersection of two faces (111) and plane (1$\bar{1}\bar{1}$)] exhibit an angle of 60°. The angle of 60° shows in the SEM image clearly demonstrates that the etching direction of MOFs is along facet (111). Colour scheme for chemical representation: cyan for Cu, red for O, grey for C and white for H.

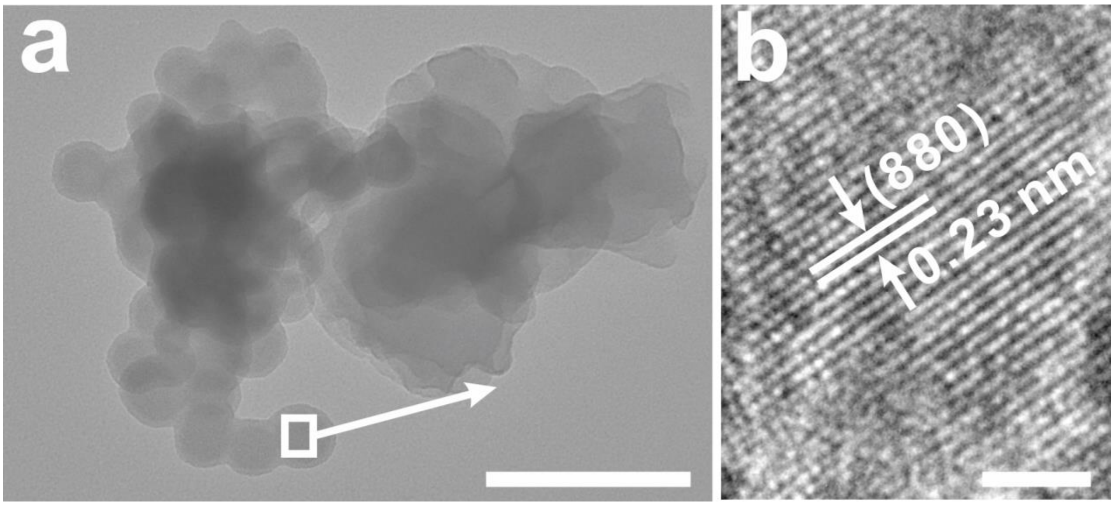

Supplementary Figure 19. (a) TEM image of small particles on CASFZU-1 surface. (b) High resolution TEM image of the rectangle region taken from the marked area in a. Scale bars, 50 nm for (a) and 2 nm for (b).

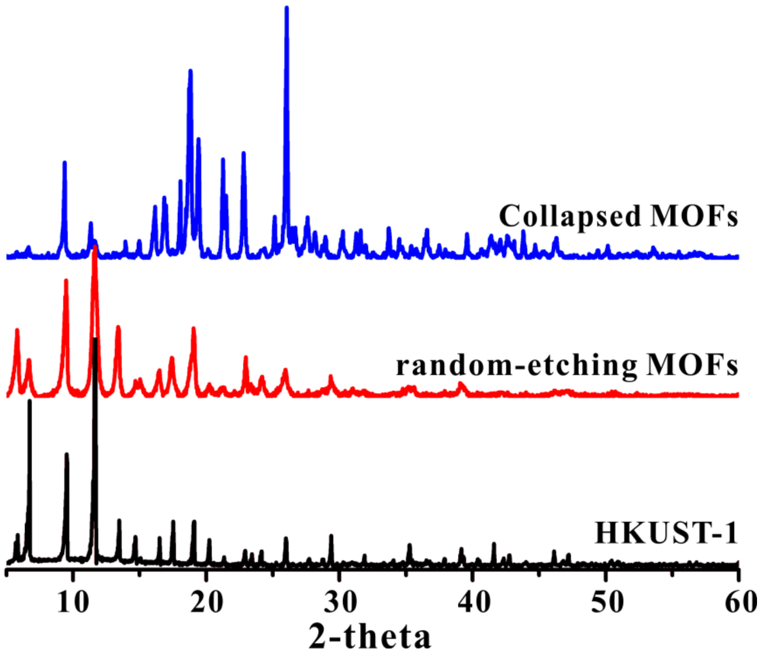

Supplementary Figure 20. XRD patterns of MOFs after degradation at different conditions.

During the random-etching process, the main structural integrity and crystallinity of MOF are well and no other visible crystals appeared. However, in the high RH condition, there is major evidence of structural changes, and the transformation is thoroughly at the end.

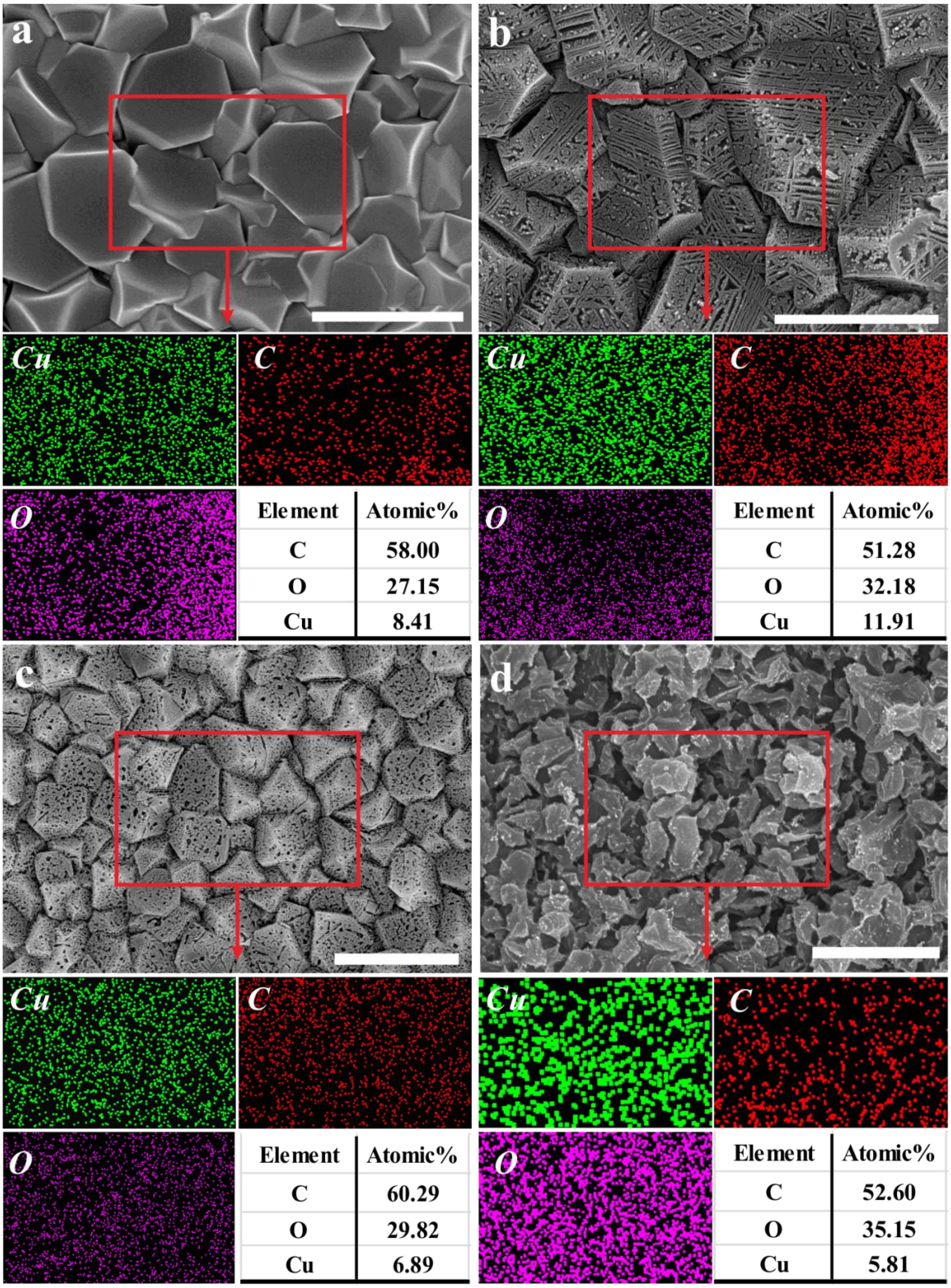

Supplementary Figure 21. SEM images of MOFs and their energy dispersive X-ray elemental mapping results, (a) for HKUST-1, (b) for CASFZU-1, (c) for random-etching MOFs and (d) for collapsed MOFs. Atom percentages of C, O and Cu in MOFs measured from the EDX spectra at red rectangle region in (a, b, c, d). Scale bars, 2 μm for (a), (b), (c) and (d).

Comparison with pristine MOF, the CASFZU-1 has lower content in carbon, higher

content in oxygen and copper. During the etching process of MOF, the reduced content percentage of carbon is about equal to the sum of increased content percentage of oxygen and copper. These results are in consistent with those in the corresponding evolution process of some pristine MOF to CASFZU-1 which has lost some ligands. While the MOF crystals structure collapsed in the process of etching, the content of oxygen is much higher than pristine MOF and the content of copper is lower than pristine MOF, which indicates that large amount of water molecules replace the organic ligands.

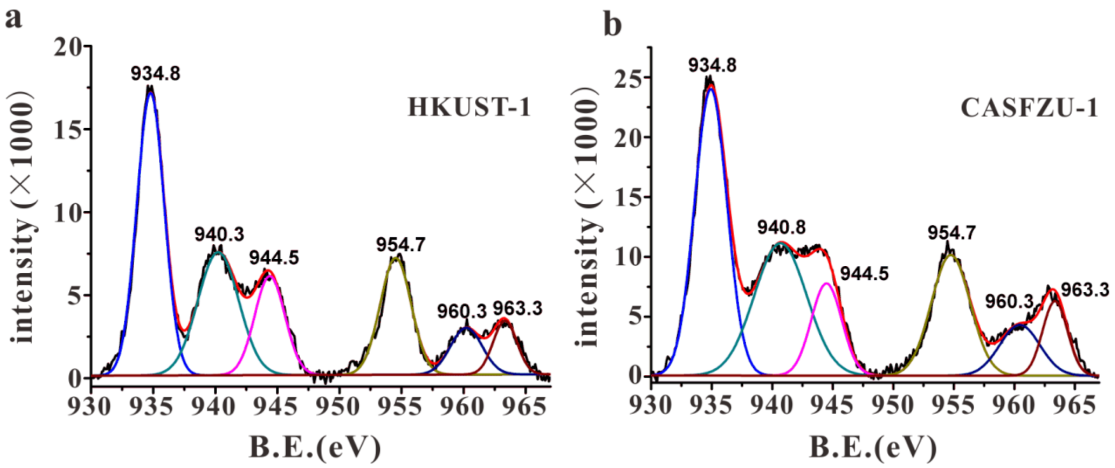

Supplementary Figure 22. (a, b) High-resolution XPS Cu 2p spectra. (a for HKUST-1, b for CASFZU-1).

Two characteristic peaks of divalent Cu^{2+} were observed at 934.8 and 954.7 eV, corresponding to Cu $2p_{3/2}$ and Cu $2p_{1/2}$, respectively. Meanwhile, the presence of the well-known ‘shake-up satellites’ (other peaks appeared in the range of 930 − 965 eV except for the two characteristic peaks) found in Cu spectra is generally considered as an indication of the presence of Cu (II) species. It is clear that etching of MOF led to a peak shift to higher energy for the Cu (II) species (shift from 940.3 eV to 940.8 eV) which indicated that copper ions in CASFZU-1 remained as divalent state after etching while the copper species become more active.

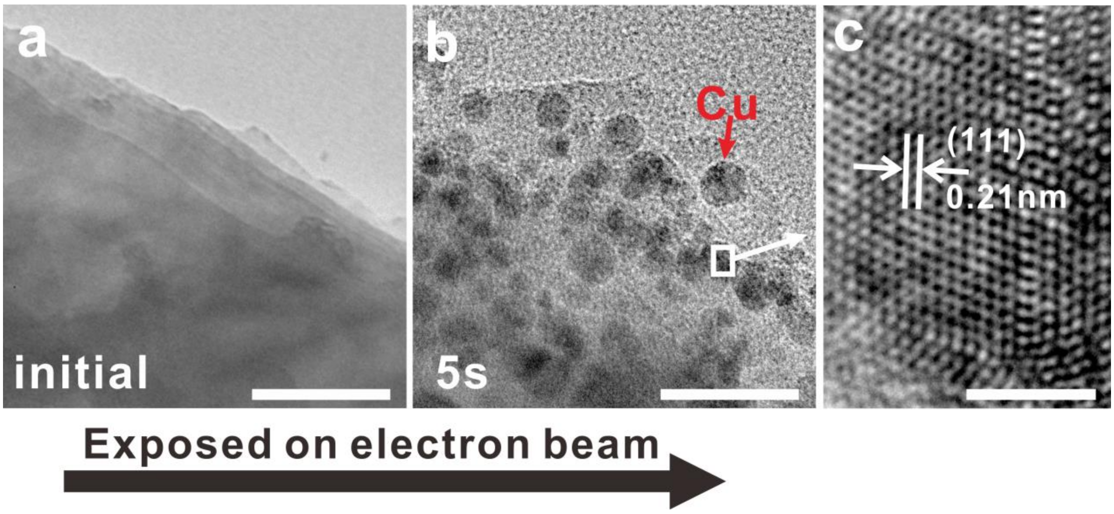

Supplementary Figure 23. (a) TEM image of CASFZU-1 nanosheets. (b) TEM image shows that the thin CASFZU-1 nanosheets rapidly degraded after electron beam irradiation in seconds. (c) High-resolution TEM image of the rectangle region taken from the marked area in b. Scale bars, 100 nm for (a), (b) and 5 nm for (c).

A high-resolution TEM (HR-TEM) image clearly confirms lattice fringes with an interplanar distance of 0.21 nm corresponds to the (111) planes of Cu crystal. These CASFZU-1 nanosheets structures were very sensitive to electron beam irradiation, which would result in an increased degradation with exposure time.

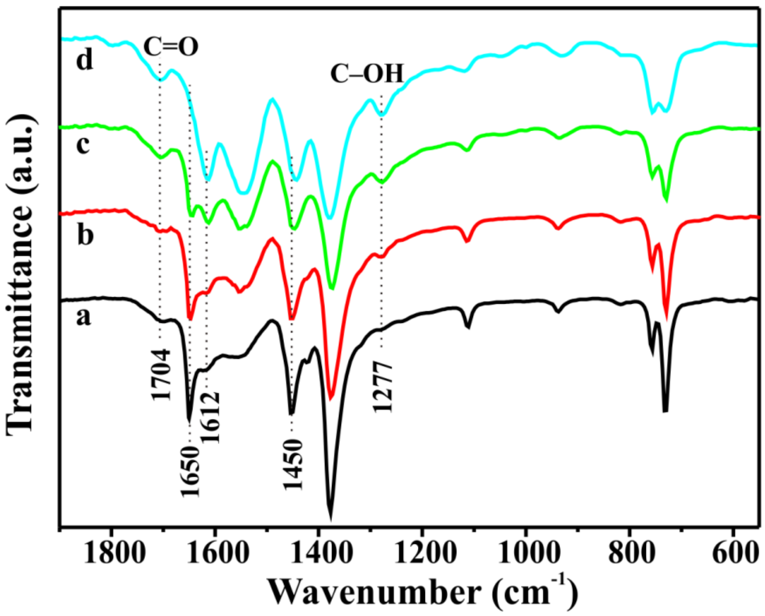

Supplementary Figure 24. In situ FT-IR spectra of the fingerprint region of MOFs dehydrated in vacuo (10^{-5} Torr) at 250 ℃. Pristine MOF (a), MOF after 1 week etching (b), MOF after 3 weeks etching (c) and MOF after 1 month etching (d).

Consistent with others in the literature, FT-IR bands are observed for the virgin HKUST-1 at 1650, 1450, 1376, 1110, 756, and 732 cm^{-1}.[17] The carboxylate symmetric stretch is present as two bands at 1450 and 1376 cm^{-1}, while the asymmetric stretch is observed at 1650 cm^{-1}. The other bands coincide with the BTC ring, as 1110 cm^{-1} is assigned to the C–H in-plane bending modes respectively, while the bands at 756 and 730 cm^{-1} coincide with the C–C ring out-of-plane bending modes.

The bands corresponding to the C–H and C–C bending modes of TMA remain relatively unchanged. The most notable changes in the spectra occur with the gradual increase in the intensity of bands at 1704 cm^{-1} and 1277 cm^{-1} corresponding to the C=O and C–OH combination band of a carboxylic acid, respectively.[18] The appearance of these new bands, along with a decrease in the intensity of the 1650 and the 1450 cm^{-1} bands, indicate the transformation of the Cu-BTC carboxylate groups to their protonated acid analogs. The above-presented observations confirmed that a certain amount of trimesic acid decoordinated or partially coordinated with copper.

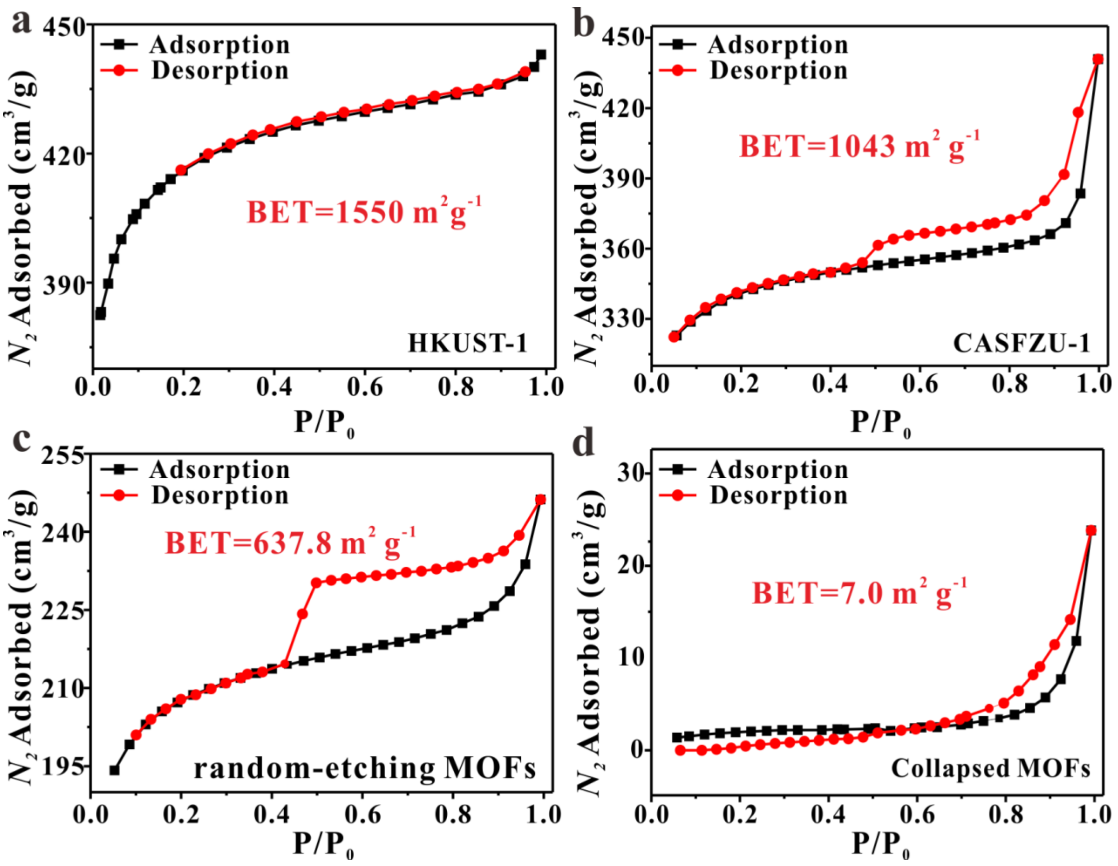

Supplementary Figure 25. (a, b, c, d) N_2 adsorption–desorption isotherms of MOFs at 77 K. (a for HKUST-1, b for CASFZU-1, c for random-etching MOFs, d for collapsed MOFs). For HKUST-1, S_{BET} =1550 m^2 g^{-1}, for CASFZU-1, S_{BET} =1043 m^2 g^{-1}, for random-etching MOFs, S_{BET} = 637.78 m^2 g^{-1}, for collapsed MOFs, S_{BET} = 7.03 m^2 g^{-1}.

The BET surface area for pristine HKUST-1 and CASFZU-1 are calculated to be 1550 and 1043 m^2 g^{-1}, respectively, suggesting that part of the inherent porous structure is blocked after some ligands fell off from the framework of MOFs. For random-etching MOFs, S_{BET} = 637.78 m^2 g^{-1}, it indicated that the **random-etching MOFs** has lost half of its porosity. For collapsed MOFs S_{BET} = 7.03 m^2 g^{-1}, it indicated that the **collapsed MOFs** has completely lost its porosity.

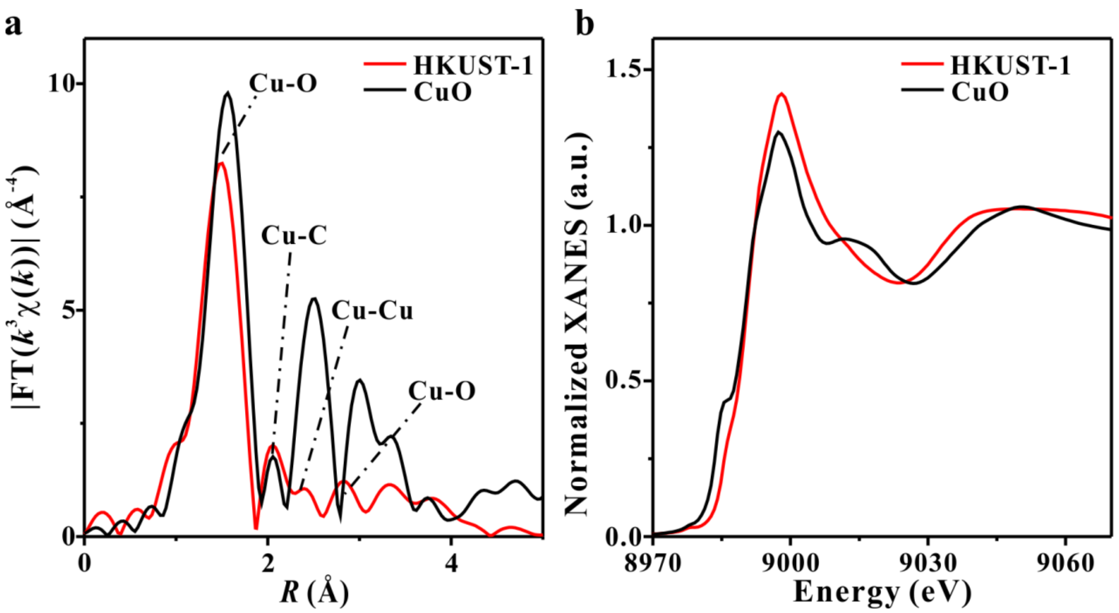

Supplementary Figure 26. (a) Fourier transform magnitudes of the Cu K-edge EXAFS experimental signal of HKUST-1 sample along with CuO standard. By comparison to CuO standard, we can obtain that the main peak at 1.50 Å can be attributed to the Cu-O bonding, while the minor peaks located at 2.05, 2.41 and 2.83 Å can arise primarily from the higher-shell Cu-C, Cu-Cu and Cu-O scattering, respectively. (b) The Cu K-edge XANES experimental spectra of HKUST-1 sample and CuO reference. As for HKUST-1, the edge position located at 8989 eV (half of the edge step) as well as the presence of the two peaks at around 8978 and 8986 eV shows that Cu species have an oxidation state of ~ +2.

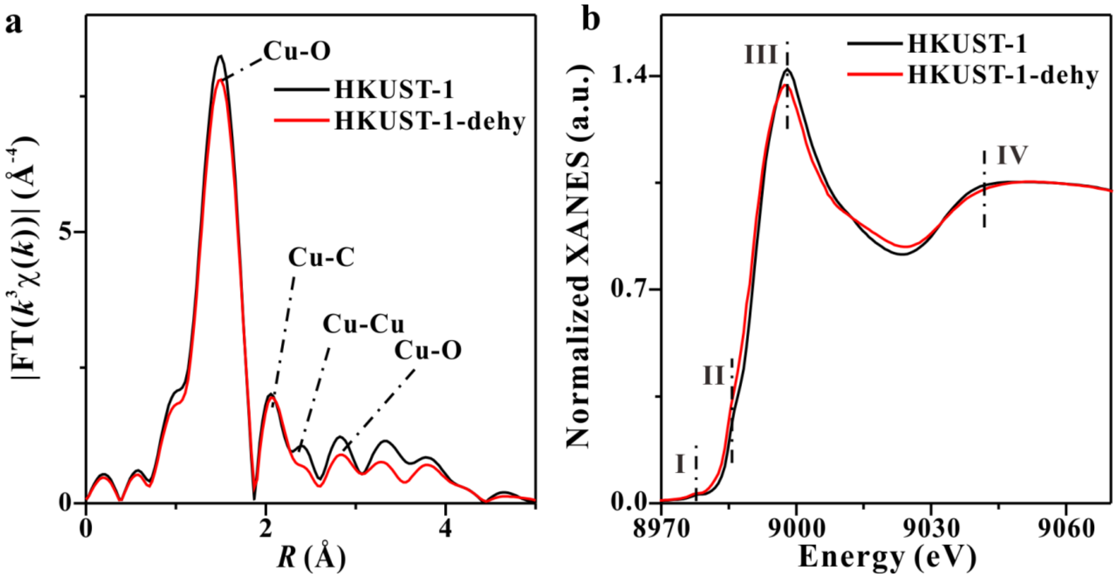

Supplementary Figure 27. (a) Fourier transform magnitudes of the Cu K-edge EXAFS experimental signal of HKUST-1 sample before and after dehydration. It can be observed that the dehydration treatment causes an obvious intensity decrease for the first Cu-O peak as well the higher-shell peaks, which indicates a partial loss of the water molecule directly linked with the Cu cations. (b) Comparison of the Cu K-edge XANES experimental spectra. From the XANES spectra, we can see that the Cu K-edge XANES is characterized by four peaks, including a pre-edge peak I at 8978 due to 1s→3d transition, a shoulder peak II at 8986 eV ascribed to 1s→4p dipolar shakedown transition, a white line peak III at 8998 eV and a resonance peak IV at 9042 eV. Upon dehydration, the Cu K-edge position does not show obvious change, which means an unaltered oxidation state for the Cu species; however, both an intensity decrease for the white line peak III and an intensity increase for the 1s→4p peak II indicate loss of axial ligands for the Cu cations, which is associated with the removal of water molecular.[19]

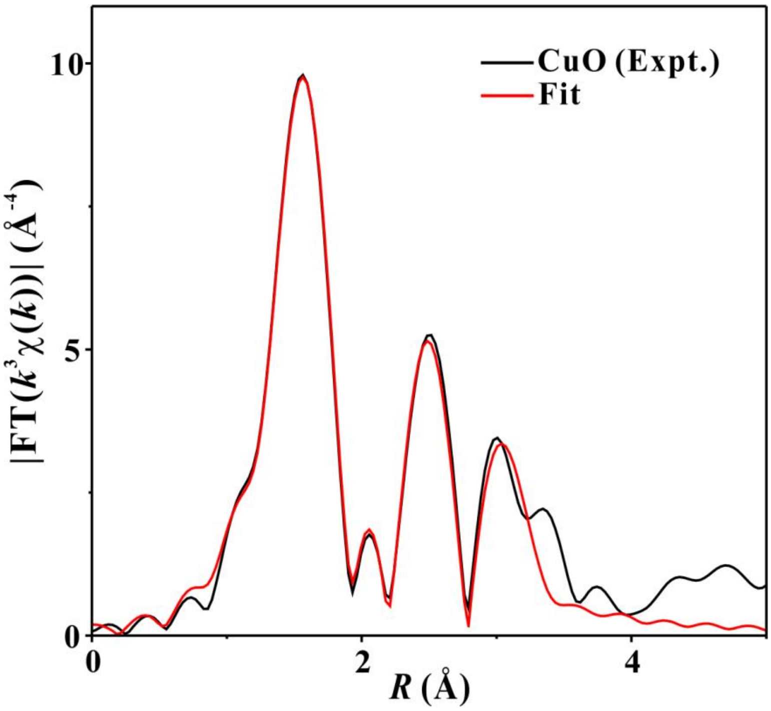

Supplementary Figure 28. Fourier-transformed magnitude of Cu K-edge EXAFS spectrum for CuO reference. Measured and calculated spectra are well matched for all samples. The best-fit parameters are shown in Supplementary Table 3.

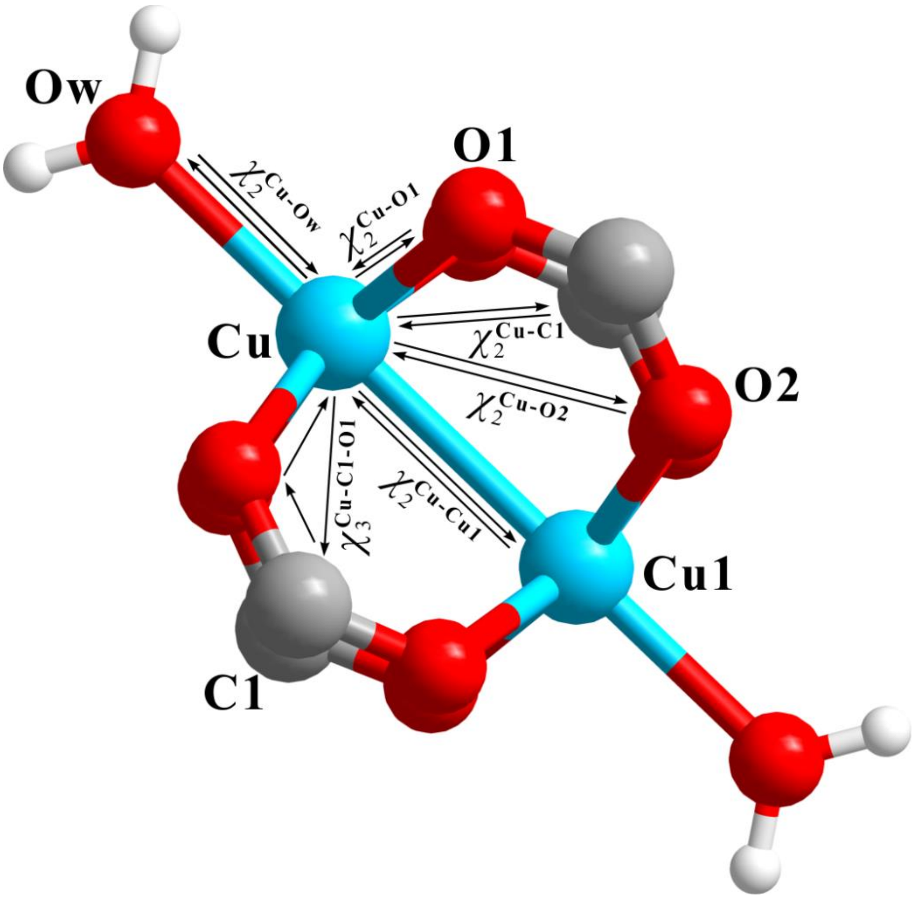

Supplementary Figure 29. Schematic model of the hydrated $[Cu_2C_4O_8](H_2O)_2$ cage used for EXAFS analysis. There are six important paths used to interpret the FT-transformed EXAFS signal in the 1.0-3.0 Å range (Figure 3a), including the Cu-O1, Cu-C1, Cu-Cu1 and Cu-O2 single-scattering (SS) paths as well as the Cu-O1-C1 double-scattering (DS) path from the carboxylate ligand and the Cu-Ow SS path from water molecule. The O1 and O2 represent the first and second nearest neighbor coordination O atoms from the carboxylate ligand. From this model structure, we can see that both the Cu-O1, Cu-C1 and Cu-O2 SS paths and the Cu-O1-C1 DS path originate from the carboxylate ligands, thus their corresponding degeneration factors are strongly correlated with each other and can be used as an indicator to detect the removal of carboxylate ligands by etching. Besides, the number of total independent data points is limited due to the deteriorative signal-to-noise when $k > 12$ Å^{-1} in the EXAFS raw data. Therefore, during the EXAFS fitting process, the degeneration factors for the Cu-O1, Cu-C1, Cu-O2 and Cu-O1-C1 DS path are constrained together to minimize the number of variables. Colour scheme for chemical representation: cyan for Cu, red for O, grey for C and white for H.

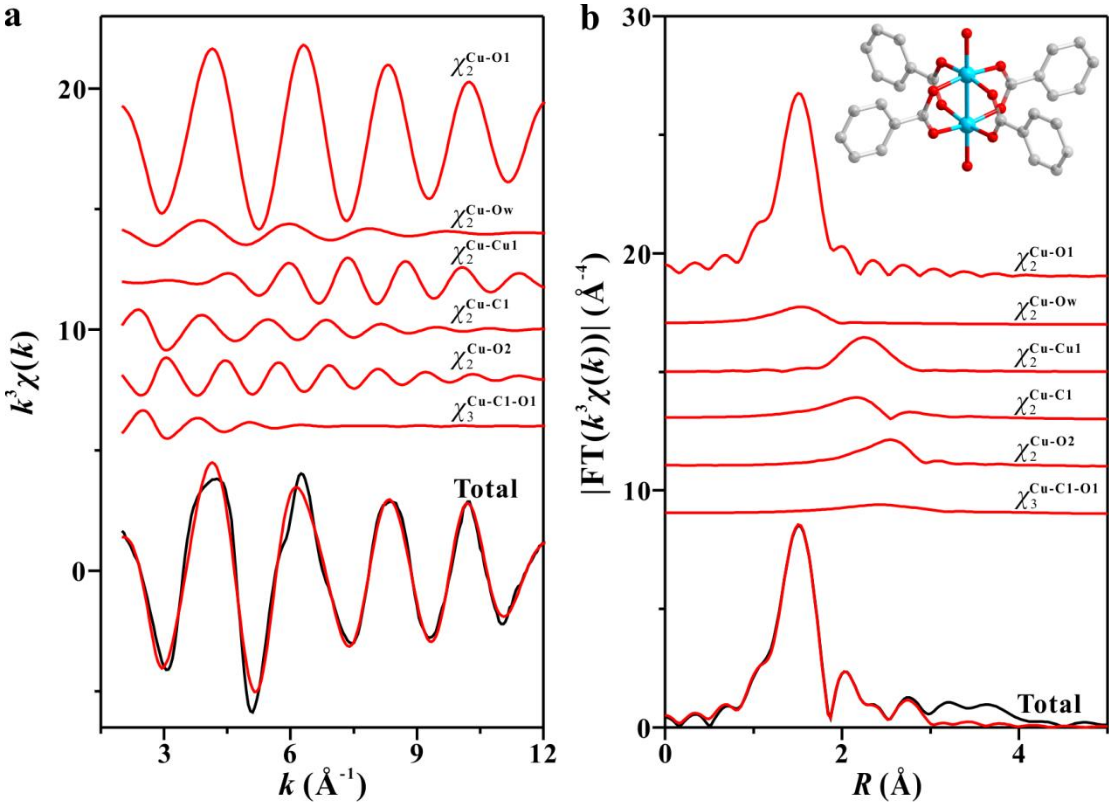

Supplementary Figure 30. (a, b) Cu K-edge EXAFS analysis of HKUST-1 sample in k and R spaces on the basis of hydrated $[Cu_2C_4O_8](H_2O)_2$ moiety. Curves from top to bottom are the single backscattering signals χ_2 and double scattering signal χ_3 included in the fit and the total signal (red line) superimposed on the experimental one (black line). The measured and calculated spectra show excellent agreement. The best-fit parameters are shown in Supplementary Table 3.

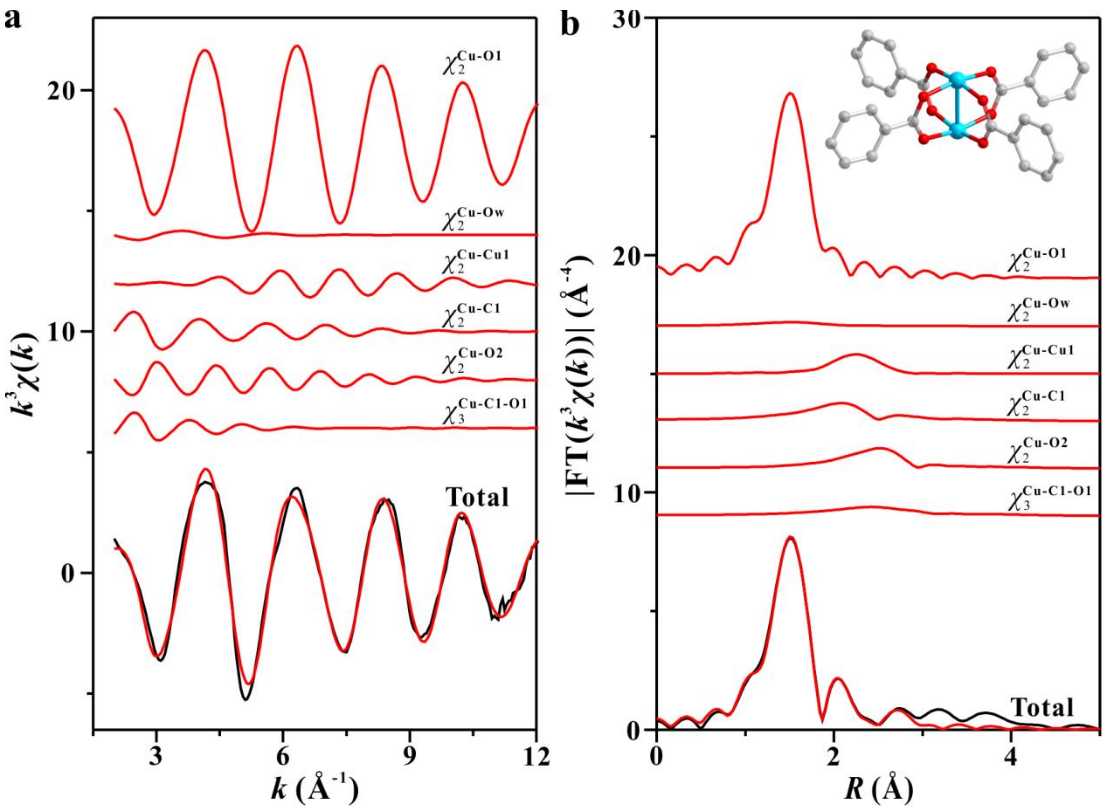

Supplementary Figure 31. (a, b) Cu K-edge EXAFS analysis of HKUST-1-dehy sample (i.e., HKUST-1 after dehydration) in *k* and *R* spaces on the basis of hydrated $[Cu_2C_4O_8](H_2O)_2$ moiety. Curves from top to bottom are the single backscattering signals χ_2 and double scattering signal χ_3 included in the fit and the total signal (red line) superimposed on the experimental one (black line). The measured and calculated spectra show excellent agreement. The best-fit parameters are shown in Supplementary Table 3.

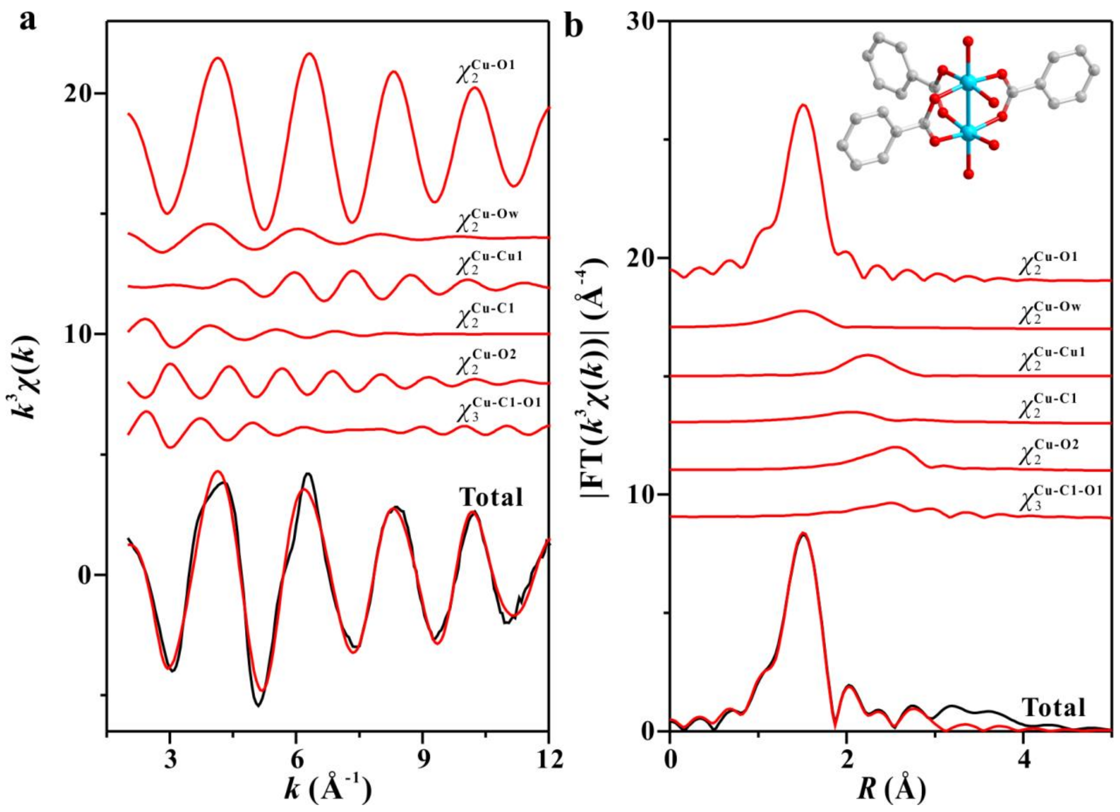

Supplementary Figure 32. (a, b) Cu K-edge EXAFS analysis of CASFZU-1 sample in k and R spaces on the basis of hydrated $[Cu_2C_4O_8](H_2O)_2$ moiety. Curves from top to bottom are the single backscattering signals χ_2 and double scattering signal χ_3 included in the fit and the total signal (red line) superimposed on the experimental one (black line). The measured and calculated spectra show excellent agreement. The best-fit parameters are shown in Supplementary Table 3.

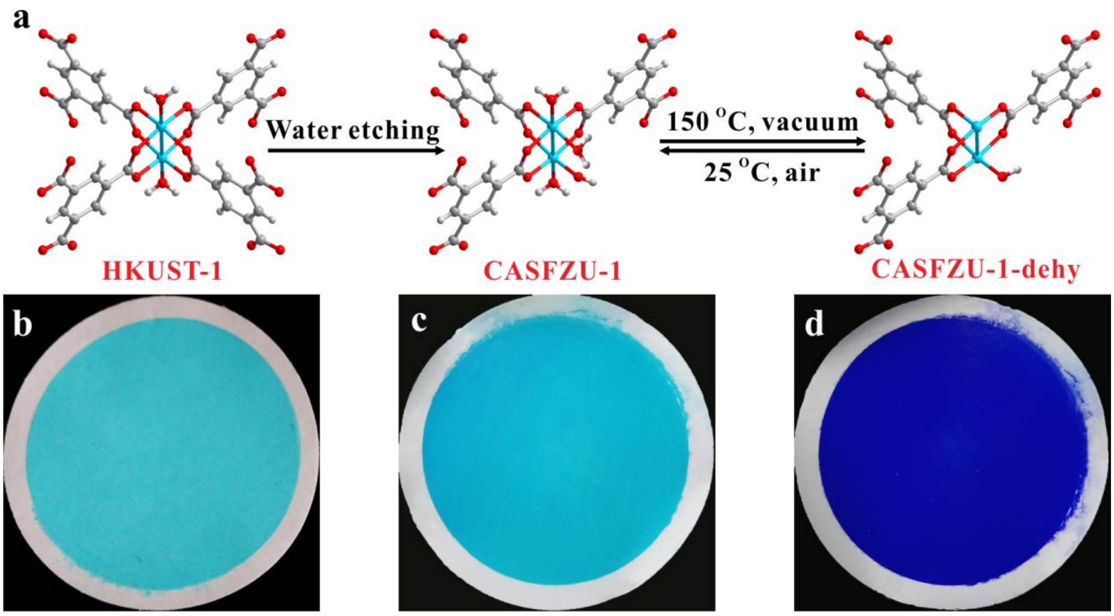

Supplementary Figure 33. (a) Schematic illustration for the coordination states of copper atoms in the MOF crystals. (b) The pristine HKUST-1 thin film. (c) The CASFZU-1 thin film at 25 ℃ in the air. (d) The CASFZU-1-dehy thin film is dried in vacuum at 150 ℃. The RH is 25%.

In fact, at room temperature and in air, the copper atoms on the surface of nanosheets are coordinated with the water molecules from the air (the colour of MOFs thin film is blue). The coordinated water molecules can be removed after dried in vacuum at 150 ℃ (the colour of MOFs thin film change from blue to bluish violet).

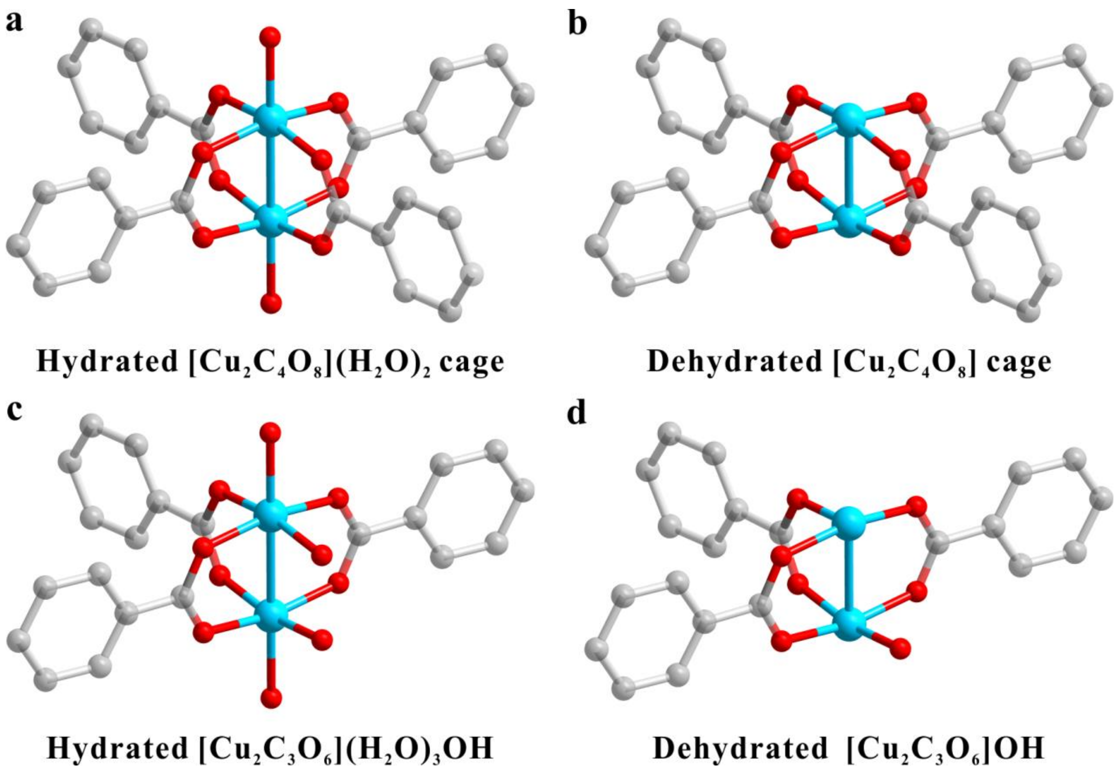

Supplementary Figure 34. Schematic of all the plausible coordination structures for Cu paddlewheel in the MOF framework. (a) The hydrated $[Cu_2C_4O_8](H_2O)_2$ cage. (b) The dehydrated $[Cu_2C_4O_8]$ cage. (c) The hydrated three-coordinated $[Cu_2C_3O_6](H_2O)_3OH$ cage. (d) The dehydrated three-coordinated $[Cu_2C_3O_6]OH$ paddlewheel model, where one of the two coordinatively unsaturated Cu atoms is directly bound by one hydroxide anion OH^-.

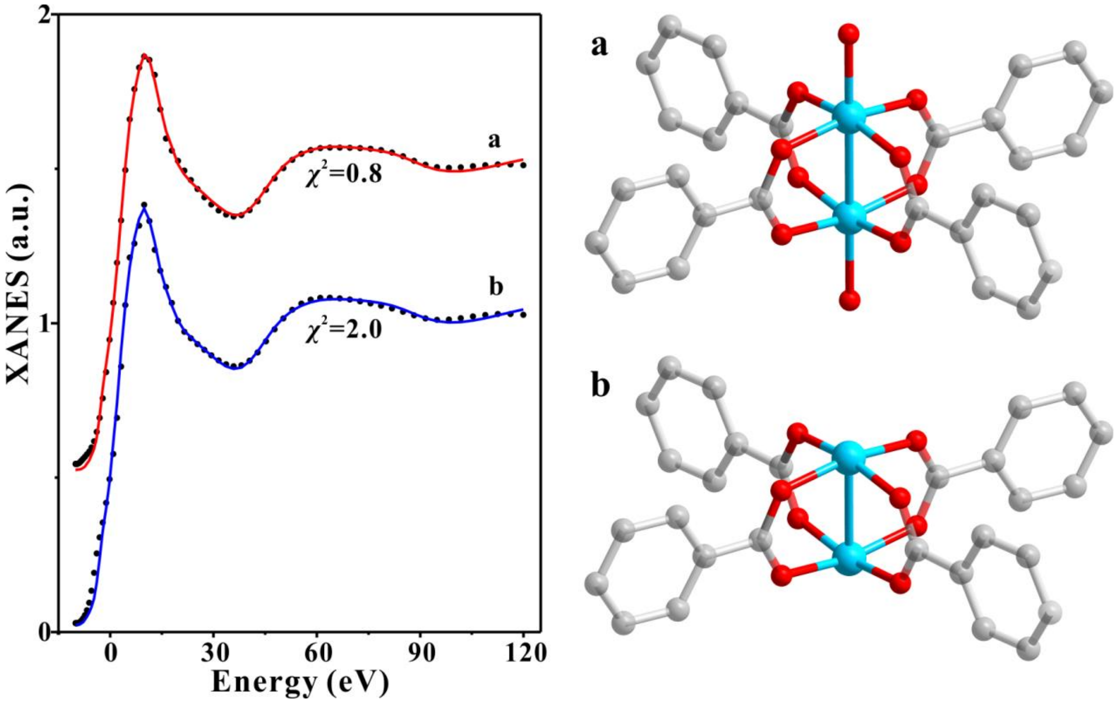

Supplementary Figure 35. Comparison between the Cu K-edge XANES experimental spectrum of HKUST-1-dehy (i.e., HKUST-1 upon dehydration, dotted lines) and the theoretical spectrum calculated with the depicted structures (solid lines). (a) The hydrated $[Cu_2C_4O_8](H_2O)_2$ moiety. (b) The dehydrated $[Cu_2C_4O_8]$ moiety. It can be seen that the experimental and theoretical spectra show better agreement for the hydrated $[Cu_2C_4O_8](H_2O)_2$ moiety than the dehydrated $[Cu_2C_4O_8]$ moiety, which suggest that the framework is dominated by the hydrated $[Cu_2C_4O_8](H_2O)_2$ moiety and thus the water molecular in HKUST-1-dehy is not thoroughly removed.

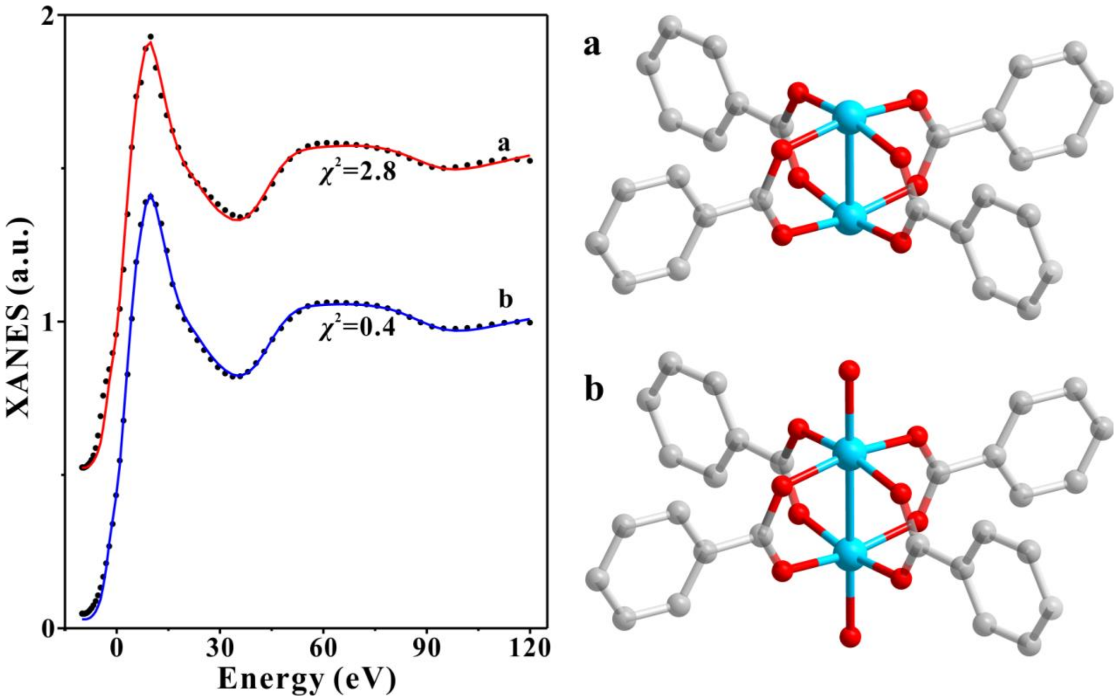

Supplementary Figure 36. Comparison between the Cu K-edge XANES experimental spectrum of CASFZU-1 (dotted lines) and the theoretical spectrum calculated with the depicted structures (solid lines). (a) The dehydrated $[Cu_2C_4O_8]$ moiety. (b) The hydrated $[Cu_2C_4O_8](H_2O)_2$ moiety. The poor agreement for the dehydrated $[Cu_2C_4O_8]$ moiety excludes their presence in the CASFZU-1 structure.

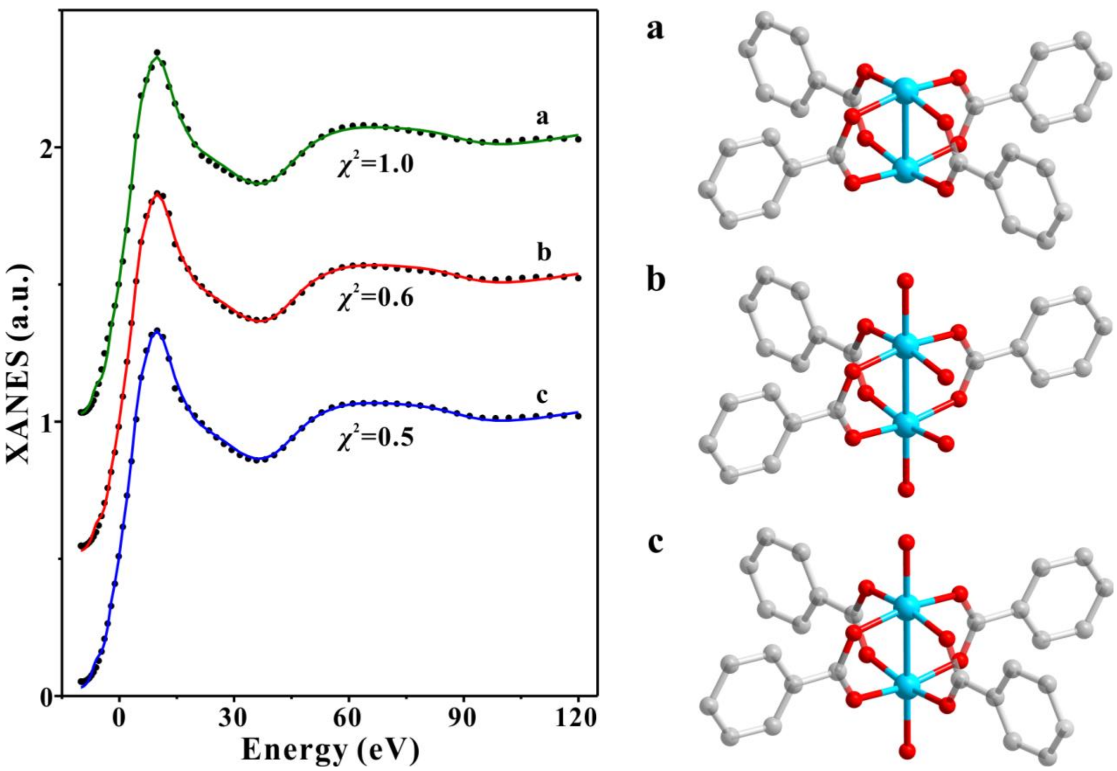

Supplementary Figure 37. Comparison between the Cu K-edge XANES experimental spectrum of CASFZU-1-dehy (i.e., CASFZU-1 upon dehydration, dotted lines) and the theoretical spectrum calculated with the depicted structures (solid lines). (a) The dehydrated [$Cu_2C_4O_8$] moiety. (b) The etched [$Cu_2C_3O_6$](H_2O)$_3$OH moiety. (c) The hydrated [$Cu_2C_4O_8$](H_2O)$_2$ moiety.

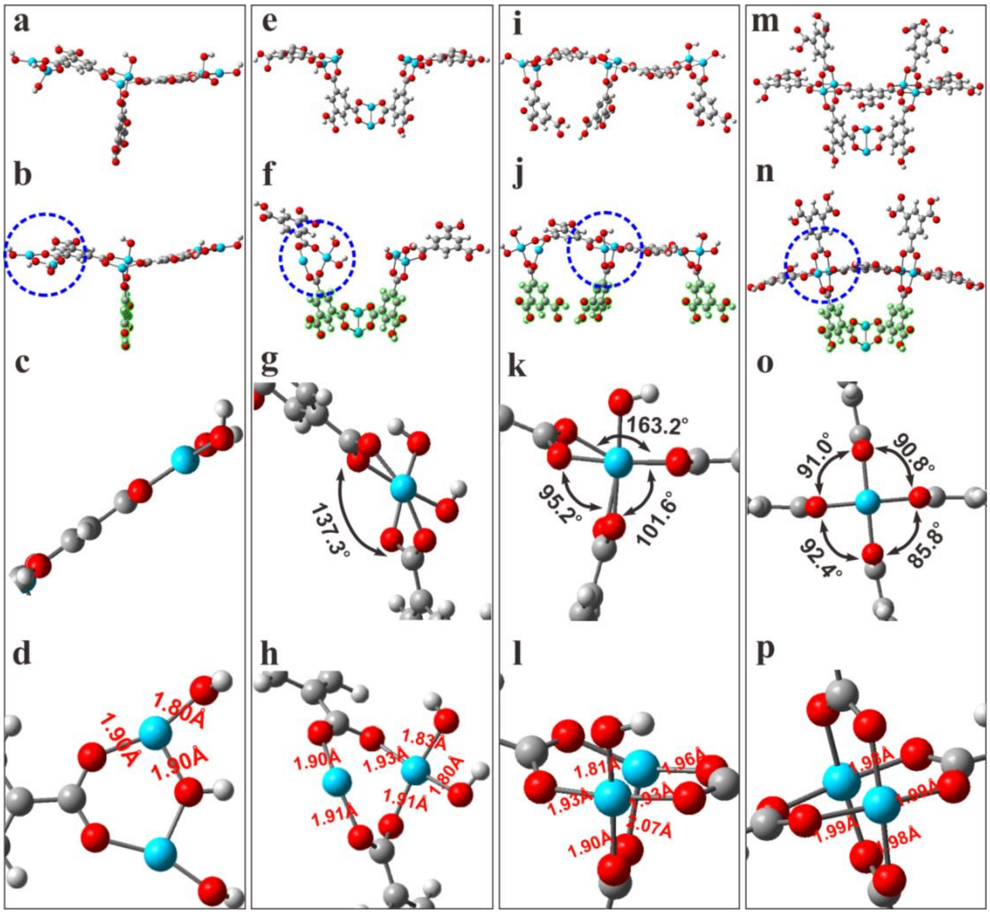

Supplementary Figure 38. **The structures from DFT calculations (without water).** (a) The input model structure of the one-coordinated paddlewheel Cu_2 cluster. (b, c, d) The optimised equilibrium structure of the one-coordinated paddlewheel Cu_2 cluster from DFT calculation. (e) The input model structure of the two-coordinated paddlewheel Cu_2 cluster. (f, g, h) The optimised equilibrium structure of the two-coordinated paddlewheel Cu_2 cluster from DFT calculation. (i) The input model structure of the three-coordinated paddlewheel Cu_2 cluster. (j, k, l) The optimised equilibrium structure of the three-coordinated paddlewheel Cu_2 cluster from DFT calculation. (m) The input model structure of the four-coordinated paddlewheel Cu_2 clusters. (n, o, p) The optimised equilibrium structure of the four-coordinated paddlewheel Cu_2 cluster from DFT calculation. The interior parts of the materials, namely these atoms marked by green colour in the models were frozen in the optimization progress. The structures in c, g, k, o, d, h, l, p are taken from the relevant blue circles in b, f, j, n in different views. All the statistical average of bond lengths is shown in Supplementary Table 4. Colour scheme for chemical representation: cyan for Cu, red for O, grey for C and white for H.

Large deformations were observed for the one- and two- coordinated paddlewheel Cu_2 cluster, while the three- and four- coordinated paddlewheel Cu_2 cluster maintain their main structures.

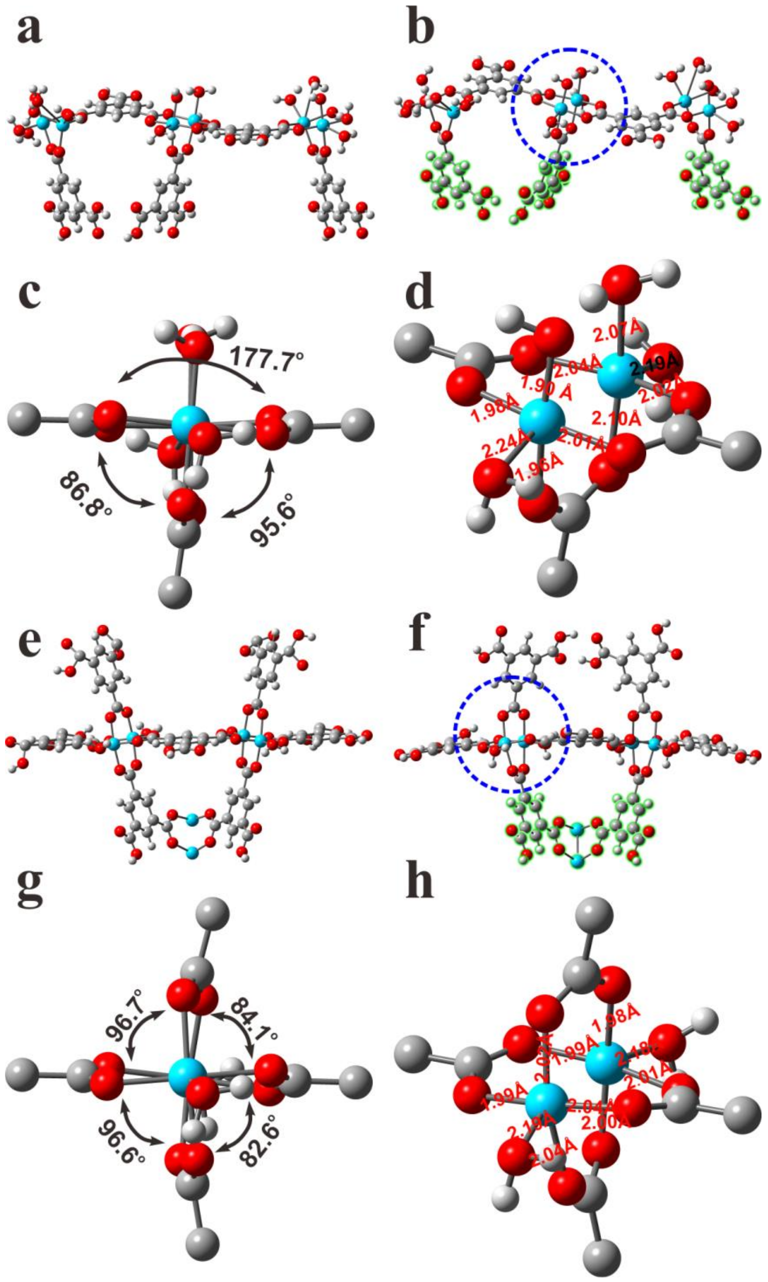

Supplementary Figure 39. **The structures from DFT calculations (with water).** (a) The input model structure of the three-coordinated paddlewheel Cu_2 clusters. (b, c, d) The optimised equilibrium structure of the three-coordinated paddlewheel Cu_2 clusters from DFT calculation. (e) The input model structure of the four-coordinated paddlewheel Cu_2 clusters. (f, g, h) The optimised equilibrium structure of the four-coordinated paddlewheel Cu_2 clusters from DFT calculation. The interior parts of the materials, namely these atoms marked by green colour in the models were frozen in the optimization progress. The structures in c, d, g, h are taken from the relevant blue circles in b, f in different views. All the statistical average of bond lengths is shown in Supplementary Table 4. Colour scheme for chemical representation: cyan for Cu, red for O, grey for C and white for H.

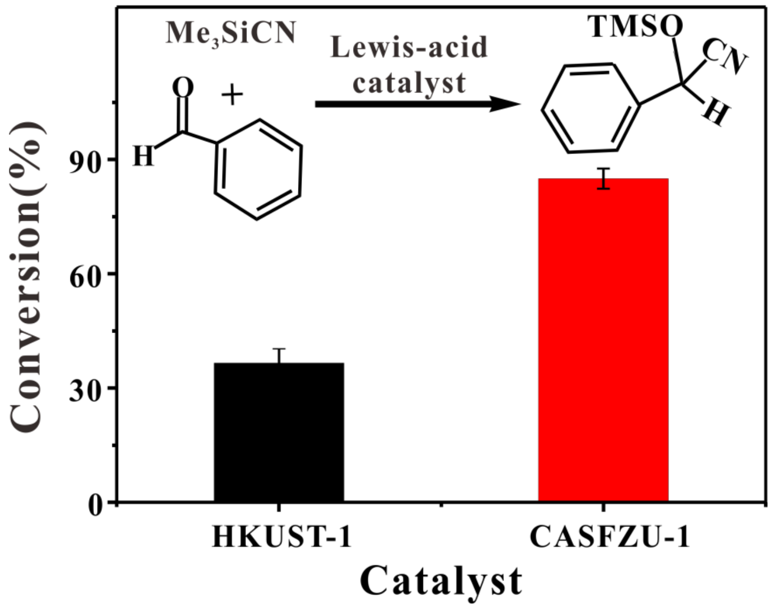

Supplementary Figure 40. Yields of the catalytic cyanosilylation of aldehydes by the two catalysts. Error bars represent the standard deviation of three replicate samples.

From the heterogeneous catalysis result that cyanosilylation of benzaldehyde and trimethylsilylcyanide over the pristine HKUST-1 and CASFZU-1, it is clearly suggested that the Lewis-acid catalytic activity of CASFZU-1 nanosheets were greatly increased after three-coordinated copper sites exposed.

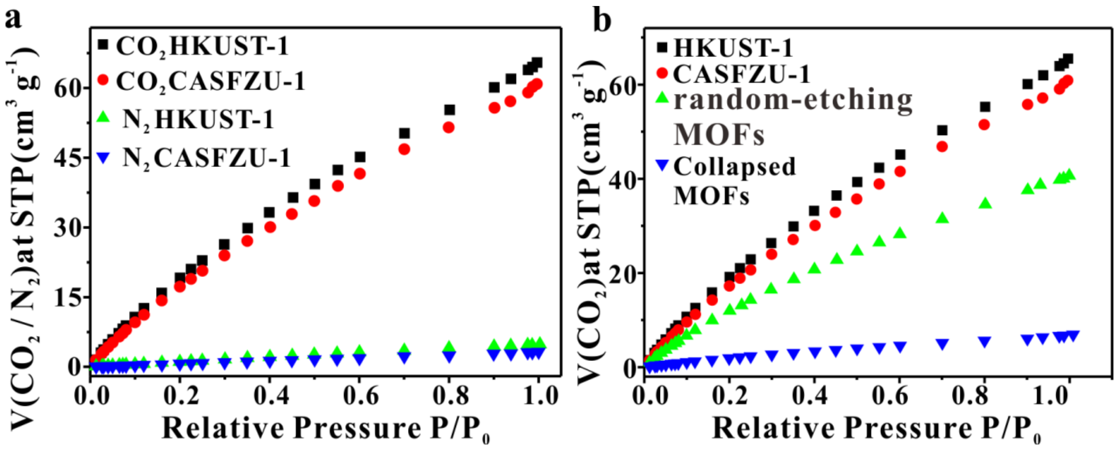

Supplementary Figure 41. (a, b) CO_2 and N_2 adsorption isotherms of the materials at 298 K.

The isotherms exhibit a type I shape with CO_2 uptakes at 1 atm of 65.51 $cm^3\ g^{-1}$, 60.89 $cm^3\ g^{-1}$, 40.65 $cm^3\ g^{-1}$ and 6.93 $cm^3\ g^{-1}$ for HKUST-1, CASFZU-1, random-etching MOFs and collapsed MOFs, respectively. N_2 uptakes at 1 atm of 4.84 $cm^3\ g^{-1}$ and 3.23 $cm^3\ g^{-1}$ for HKUST-1 and CASFZU-1, respectively.

Supplementary Note 2. The rate law for the cycloaddition of CO_2 with epoxide

The rate law for the cycloaddition of CO_2 with epoxide using the Lewis acid has been determined.[20] The rate equation is ultimately described by Eq. (10):

$$\mathbf{Rate} = k[\mathbf{epoxide}]^1[\mathbf{CO_2}]^1[\mathbf{cat.}]^1[\mathbf{co\text{-}cat}]^2 \tag{10}$$

The catalyst in this paper is MOF, and the co-catalyst is TBAB.

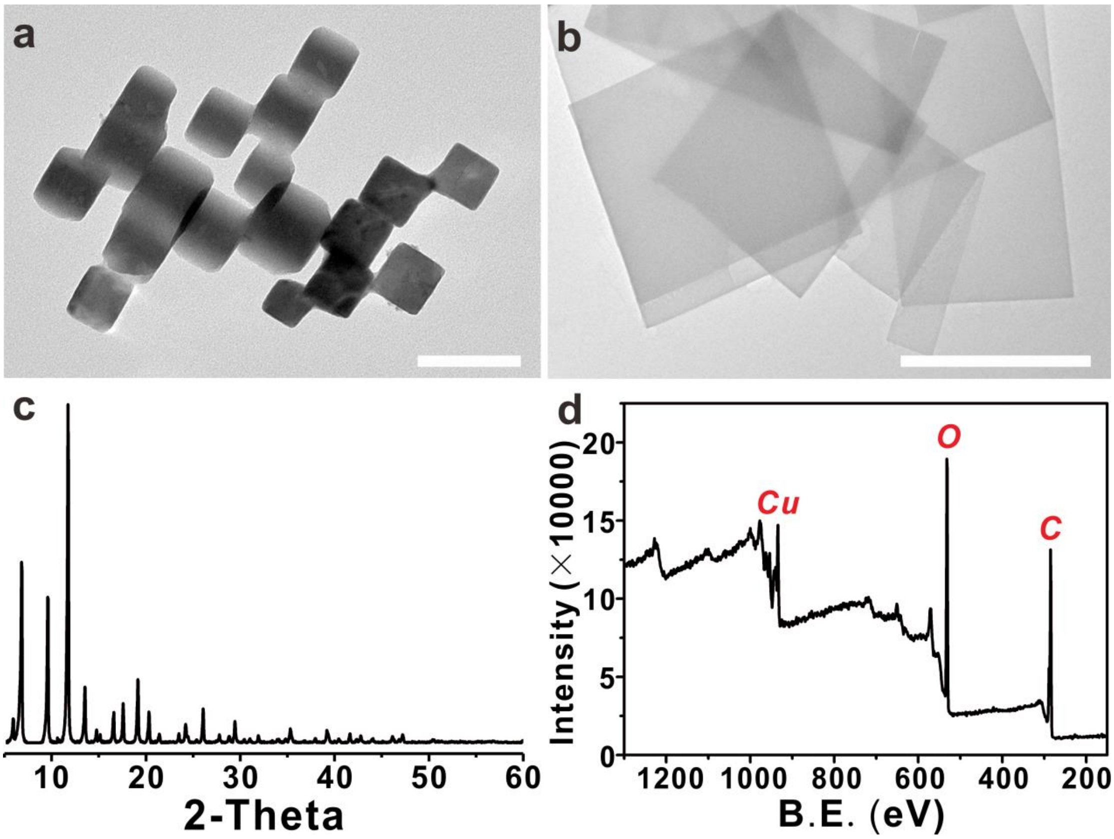

Supplementary Figure 42. TEM images of Cu_2O nanocubes. (b) TEM images of the HKUST-1 nanosheets. (c) XRD pattern of HKUST-1 nanosheets. (d) XPS pattern of HKUST-1 nanosheets. Scale bars, 100 nm for (a), 2 μm for (b).

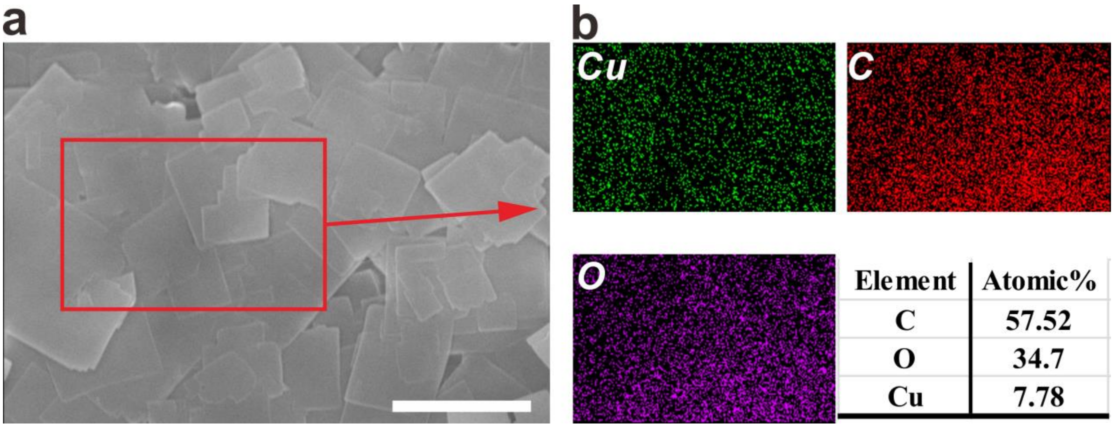

Supplementary Figure 43. (a) SEM image of the freestanding HKUST-1 nanosheets. (b) Energy dispersive X-ray elemental mapping results collected from the insert red rectangle region in a. The atomic% of Cu is 7.78% which is about 35% lower than that in CASFZU-1 (11.91%). Scale bar, 2 μm for (a).

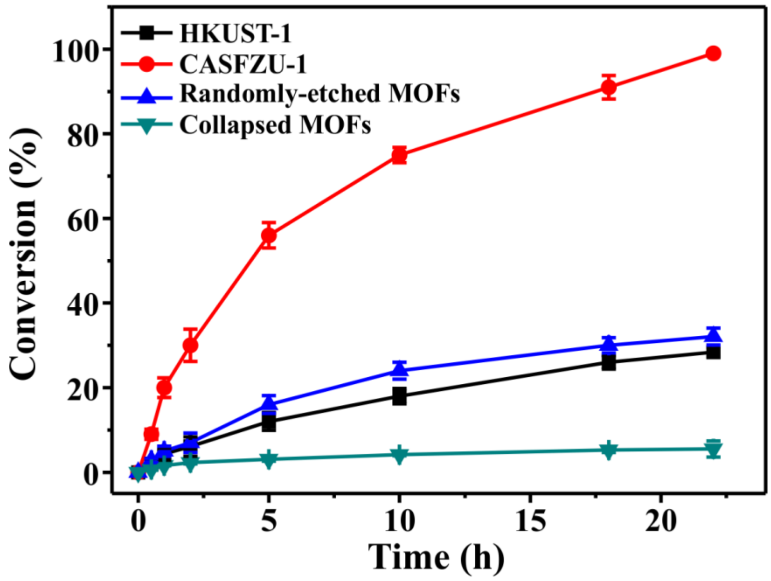

Supplementary Figure 44. Dependence of the cycloaddition conversion on the reaction time over the MOFs. (The substrate is 2-methyloxirane). Error bars represent the standard deviation of three replicate samples.

Supplementary Note 3. The amount of Carbon dioxide fixation

In present experimental conditions, 99% of 2-methyloxirane (25 mmol) has already reacted with pure CO_2 in the first 22 h; the corresponding amount of catalyst (**CASFZU-1**) is 5.5 mg:

For 5.5 mg catalyst in 24h,

$$n (CO_2) = (99 \pm 0.5)\%*25*24/22 = 27.0 \pm 0.136 \text{ mmol } (24h)^{-1},$$

So, for 1 g catalyst in 24h,

$$n (CO_2) = (27.0 \pm 0.136)*1000 / 5.5 = 4.91 \pm 0.025 \text{ mol } (24h)^{-1},$$

Carbon accumulation for the area of an **undisturbed tropical rainforest** is approximately 8.5 ± 2.0 mol m^{-2} $year^{-1}$ as previous report.[21]

Equally,

$$8.5 \pm 2.0 \text{ mol } m^{-2} \text{ year}^{-1} = (2.33 \pm 0.55)*10^{-2} \text{ mol } m^{-2}(24h)^{-1},$$

The amount of CO_2 absorbed by 211 m^2 tropical rainforest in 24 h is

$$n (CO_2) = (2.33 \pm 0.55)*10^{-2}*211 = 4.916 \pm 1.16 \text{ mol } (24h)^{-1},$$

So, in this case the amount of CO_2 absorbed by cycloaddition reaction catalyzed by 1g catalyst in 24 h is equally to the amount of CO_2 accumulation by ca. 210 m^2 tropical rainforest in 24 h.

In a similar way,

For 5.5 mg **HKUST-1** catalyst in 24h (conversion is 28.4 ± 1.1% for 22h),

$$n (CO_2) = (31 \pm 1.1)\%*25 = 8.0 \pm 0.28 \text{ mmol } (24h)^{-1},$$

So, for 1 g catalyst in 24h,

$$n (CO_2) = (8.0 \pm 0.28)*1000 / 5.5 = 1.45 \pm 0.051 \text{ mol } (24h)^{-1},$$

For 5.5mg **random-etching MOFs** catalyst in 24h (conversion is 32.0 ± 2.0% for 22h),

$$n (CO_2) = (35 \pm 2.0)\%*25 = 8.75 \pm 0.50 \text{ mmol } (24h)^{-1},$$

So, for 1 g catalyst in 24h,

$$n (CO_2) = (8.75 \pm 0.50)*1000 / 5.5 = 1.59 \pm 0.09 \text{ mol } (24h)^{-1},$$

For 5.5mg **collapsed MOFs** catalyst in 24h (conversion is 5.5 ± 1.9% for 22h),

$$n (CO_2) = (6 \pm 1.9)\%*25 = 1.5 \pm 0.475 \text{ mmol } (24h)^{-1},$$

So, for 1 g catalyst in 24h,

$$n (CO_2) = (1.5 \pm 0.475)*1000 / 5.5=0.273 \pm 0.086 \text{ mol } (24h)^{-1},$$

For 5.5 mg **HKUST-1 nanosheets** catalyst in 24h (conversion is 30.3 ± 1.6% for 22h),

$$n (CO_2) = (33 \pm 1.6)\%*25 = 8.25 \pm 0.4 \text{ mmol } (24h)^{-1},$$

So, for 1 g catalyst in 24h,

$$n (CO_2) = (8.25 \pm 0.4)*1000 / 5.5 = 1.50 \pm 0.073 \text{ mol } (24h)^{-1},$$

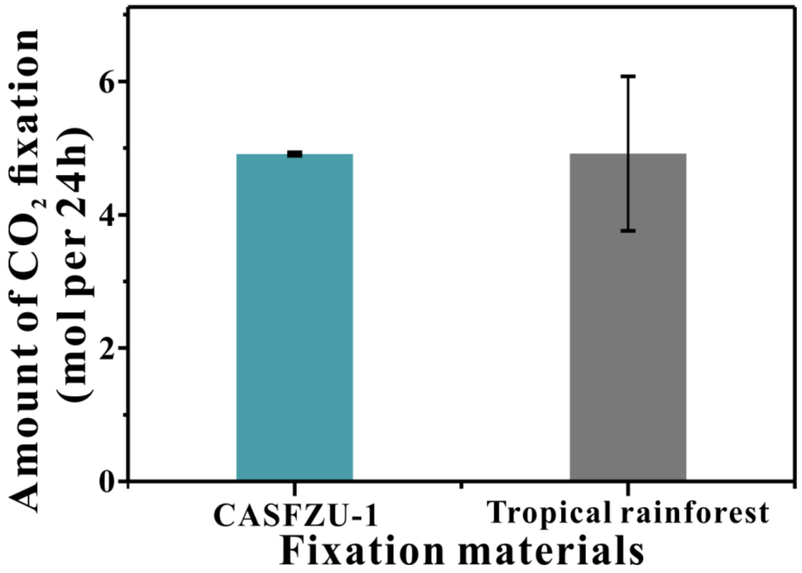

Supplementary Figure 45. A comparison of the amount of carbon fixation via chemical conversion reactions catalyzed by CASFZU-1 nanosheets and via photosynthesis process by tropical rainforest. Error bars represent the standard deviation of three replicate samples.

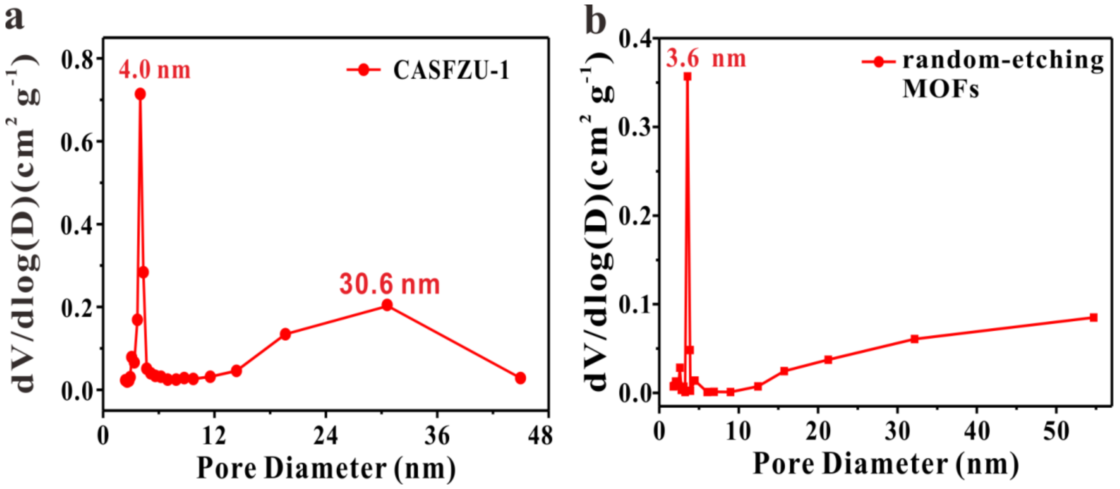

Supplementary Figure 46. Pore size distributions of CASFZU-1 and random-etching MOFs.

With the ligand lost, larger mesopore in MOFs was produced. The mesopore diameters are about 4.0 nm and 30.6 nm in CASFZU-1 which are much bigger than that in HKUST-1. We have solid reason to believe that CASFZU-1 with large mesopores will efficiently enhance its capture capacity of large molecule and will greatly improve the accessibility of target object into copper sites inside the pore. The mesopore diameters in random-etching MOFs are about 3.6 nm which is similar to the CASFZU-1.

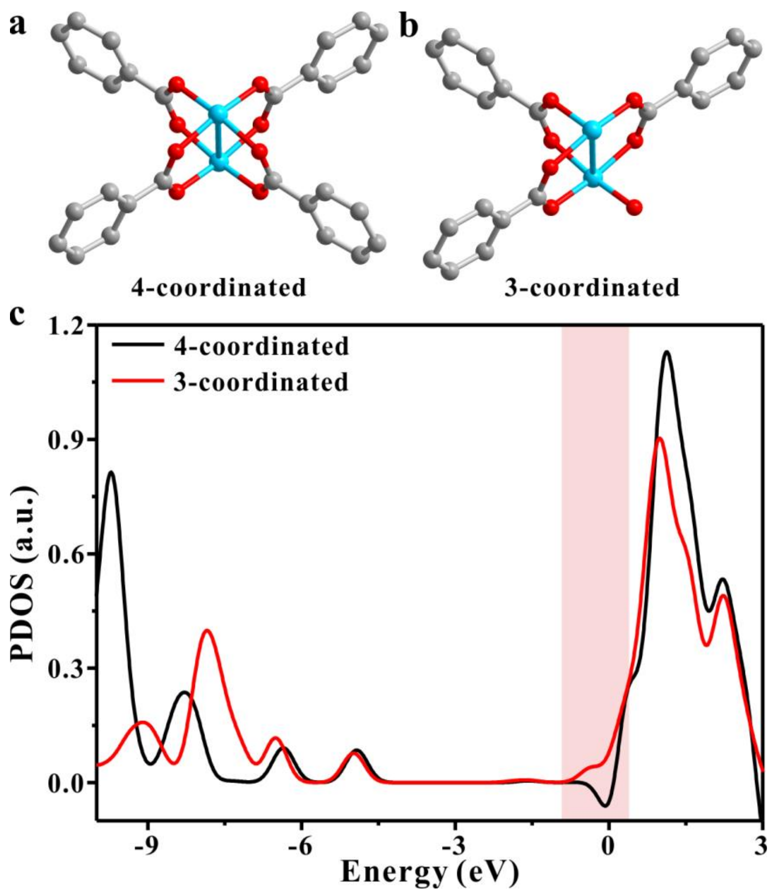

Supplementary Figure 47. (a, b) Schematic models of the paddlewheel Cu_2 clusters with two different kinds of coordination numbers. (c) Calculated relevant partial densities of states for the 4-coordinated Cu_2 cluster and the 3-coordinated Cu_2 cluster. Colour scheme for chemical representation: cyan for Cu, red for O and grey for C.

The PDOS of 4-coordinated Cu sites exhibited a very low tail at the conduction band minimum, whereas that in 3-coordinated Cu sites displayed obviously raised states density at the edge of conduction band. This greatly increased PDOS at the conduction band minimum of the 3-coordinated Cu sites could be ascribed to their exotic layered structure. Notably, the unfilled e_g states of coordinately-unsaturated Cu sites in 3-coordinated Cu sites are less than those of fully-coordinated Cu sites in HKUST-1.

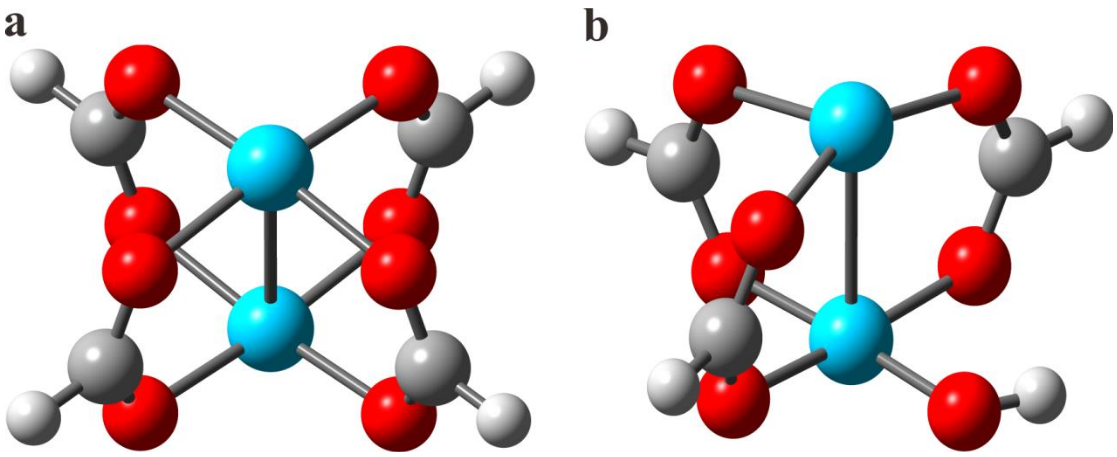

Supplementary Figure 48. The structural models of the paddlewheel Cu_2 clusters with two different kinds of coordination numbers (a) for HKUST-1, and (b) for CASFZU-1) to obtain the potential energy surface profiles of the cycloaddition reaction. Colour scheme for chemical representation: cyan for Cu, red for O, grey for C and white for H.

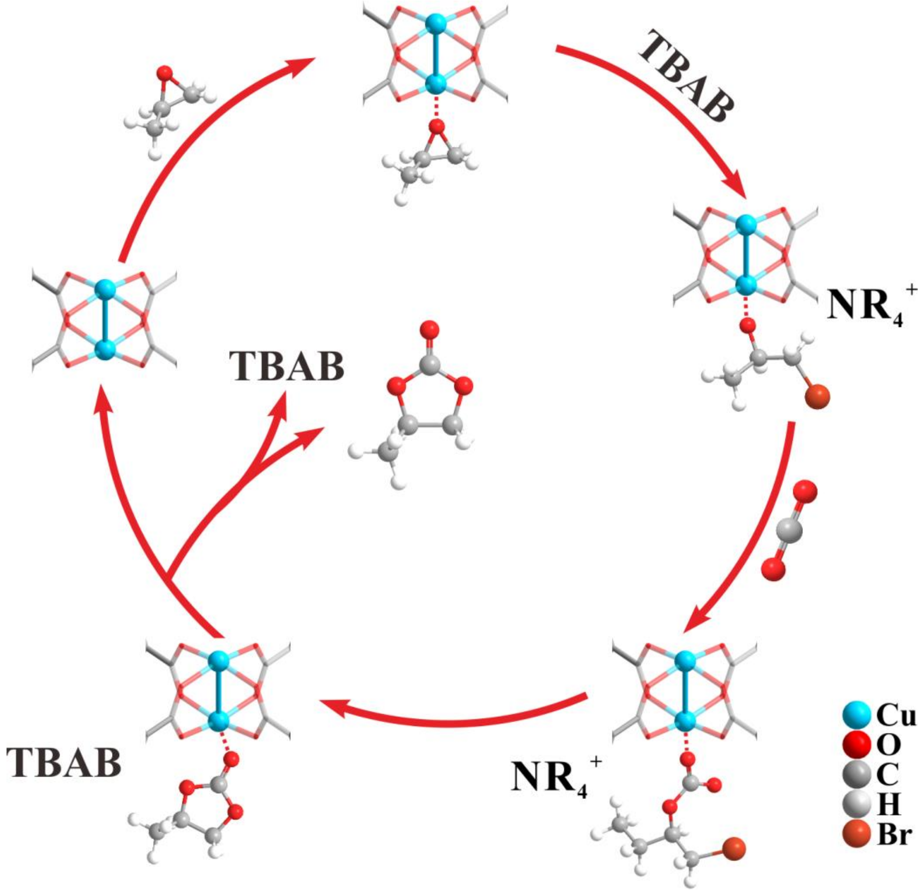

Supplementary Figure 49. Possible catalytic mechanisms for the chemical conversion of CO_2 into cyclic carbonate with 2-methyloxirane which catalyzed by paddlewheel Cu_2 cluster and TBAB. Colour scheme for chemical representation: cyan for Cu, red for O, grey for C, white for H, and tangerine for Br.

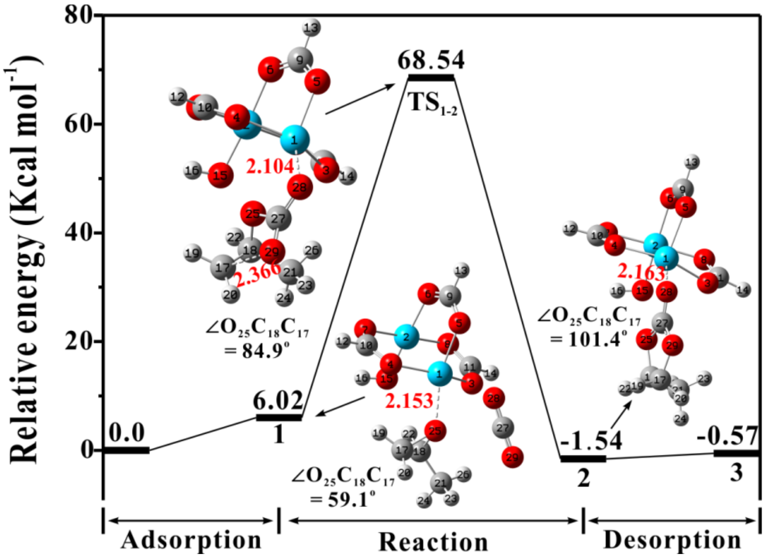

Supplementary Figure 50. Potential energy surface profile for the cycloaddition reaction without co-catalyst (TBAB). Colour scheme for chemical representation: cyan for Cu, red for O, grey for C and white for H.

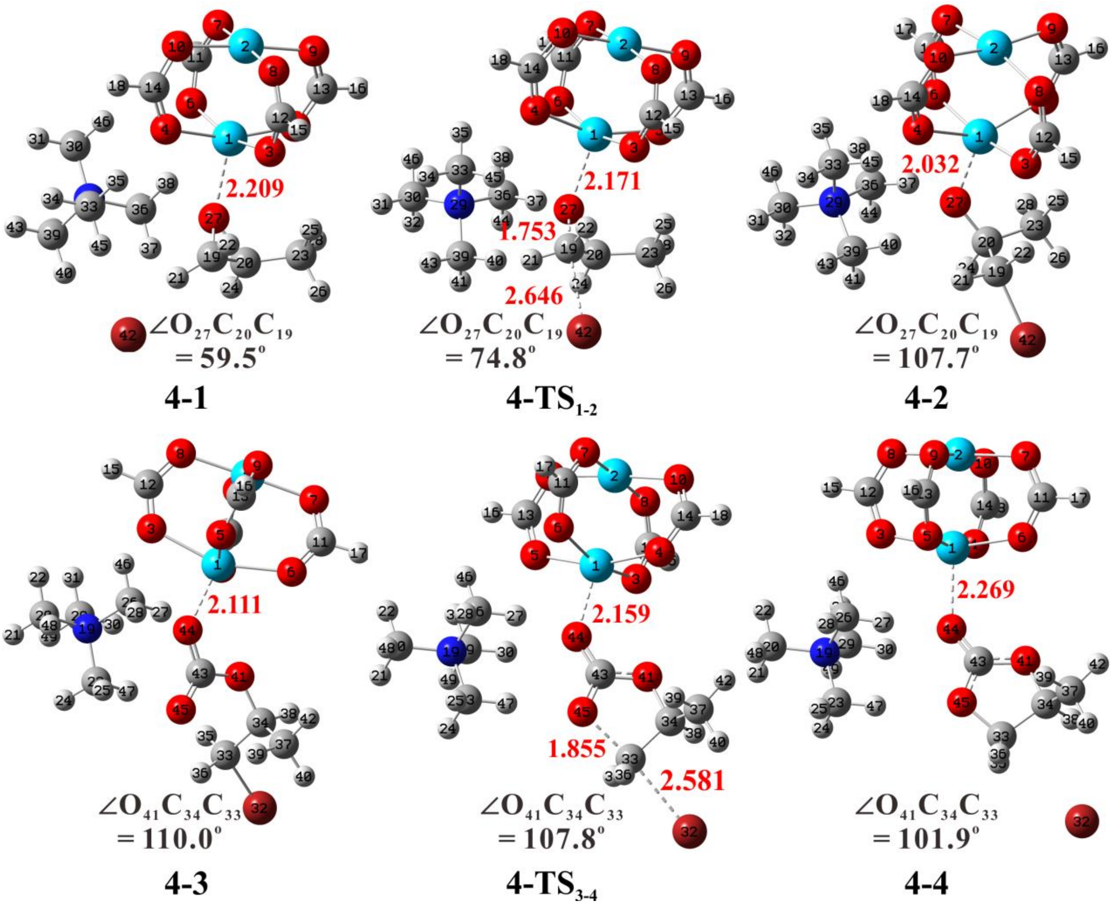

Supplementary Figure 51. Optimised geometries for the intermediates and transition states for the CO_2 fixation catalyzed by HKUST-1. Bond lengths and distances are in Å. Colour scheme for chemical representation: cyan for Cu, red for O, grey for C, white for H, blue for N, and tangerine for Br.

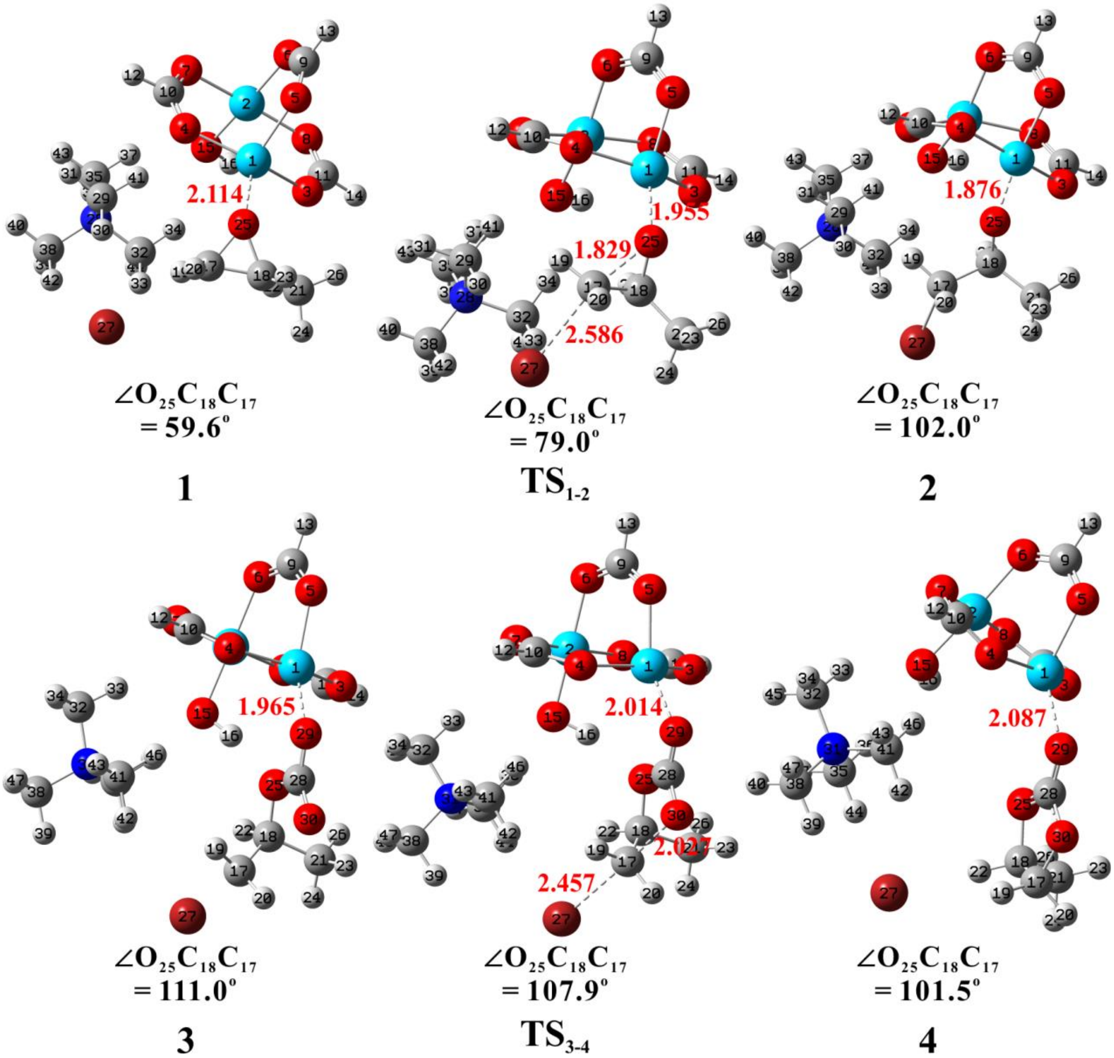

Supplementary Figure 52. Optimised geometries for the intermediates and transition states for the CO_2 fixation catalyzed by CASFZU-1. Bond lengths and distances are in Å. Colour scheme for chemical representation: cyan for Cu, red for O, grey for C, white for H, blue for N, and tangerine for Br.

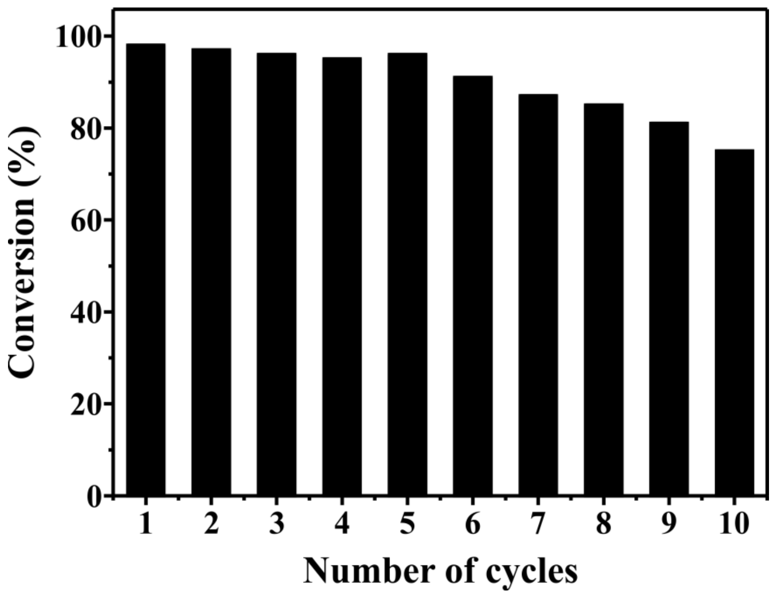

Supplementary Figure 53. Recyclability of CASFZU-1 for the cycloaddition reaction for ten runs.

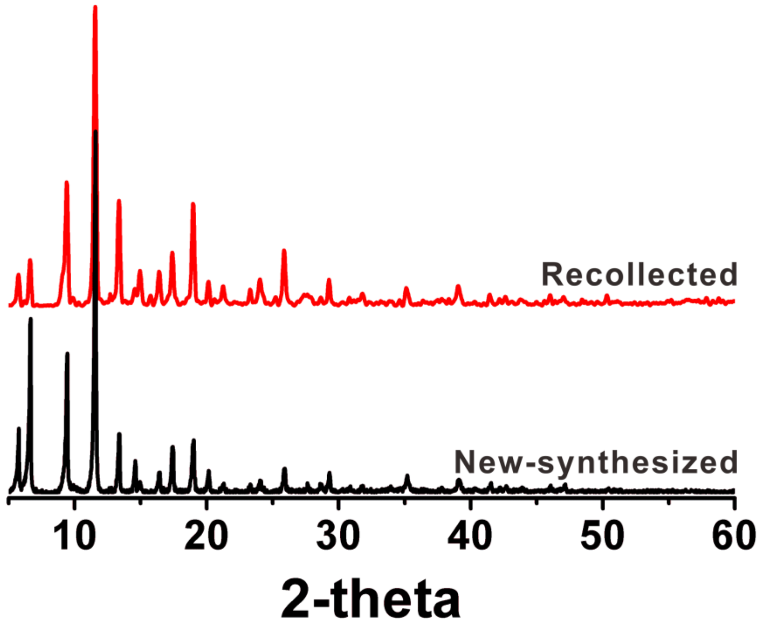

Supplementary Figure 54. PXRD patterns of CASFZU-1 recollected after catalysis reactions and calculated from the crystal data, indicating that the crystal structure was well retained after the catalysis reaction.

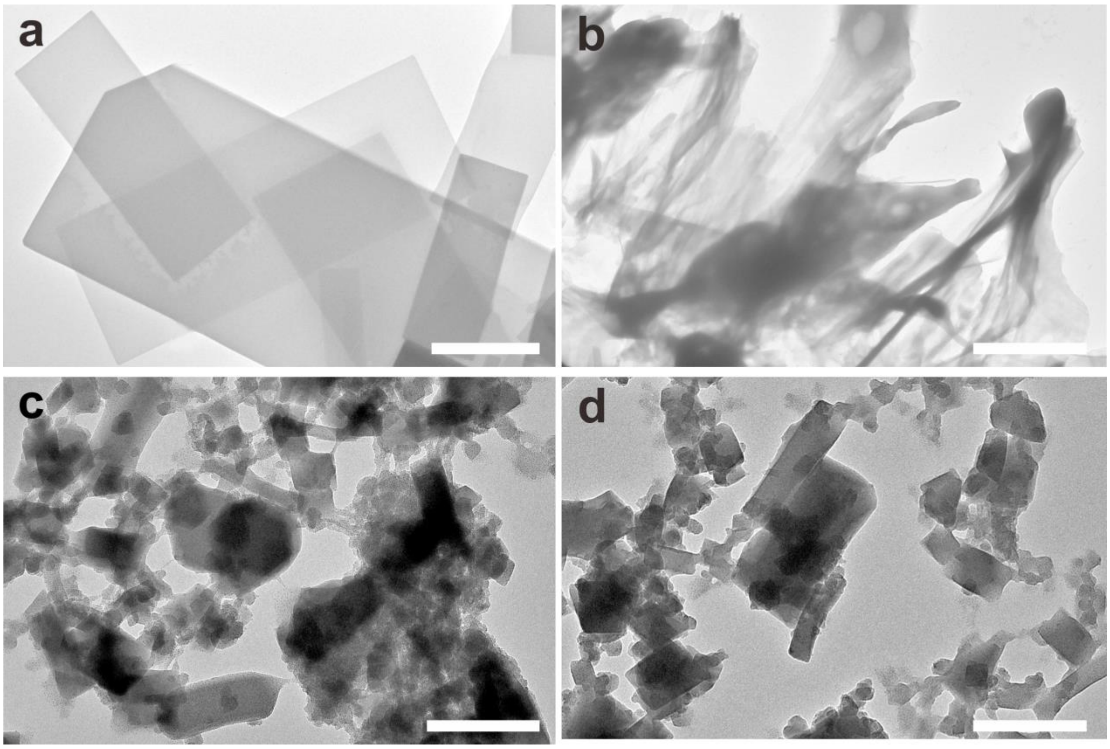

Supplementary Figure 55. (a) TEM images of the pristine HKUST-1 nanosheets. (b-d) TEM images of the HKUST-1 nanosheets recollected after catalysis reactions for one, three, five runs, respectively. Scale bars, 1 μm for (a-d).

The results indicate that the macrostructure of the HKUST-1 nanosheets was completely broken and the fragments aggregated after catalysis reactions for five runs.

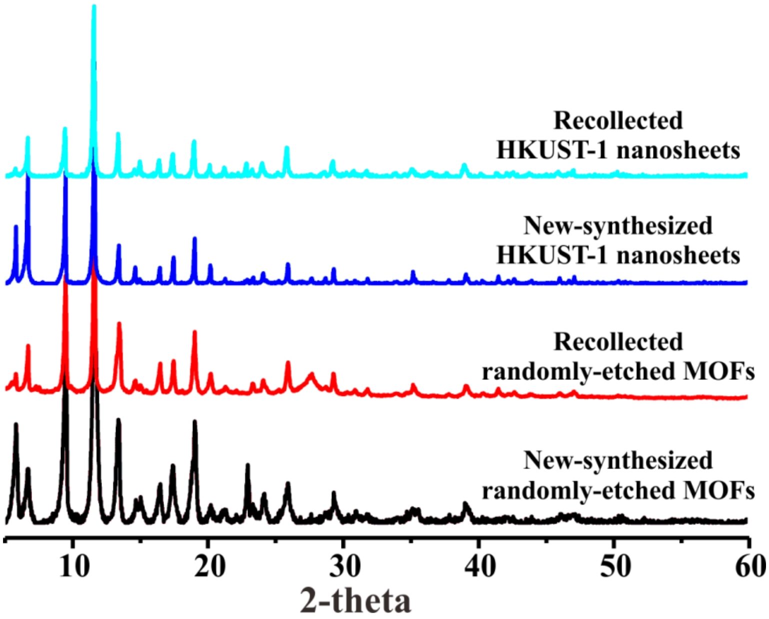

Supplementary Figure 56. PXRD patterns of randomly-etched MOFs and HKUST-1 nanosheets recollected after catalysis reaction for five runs.

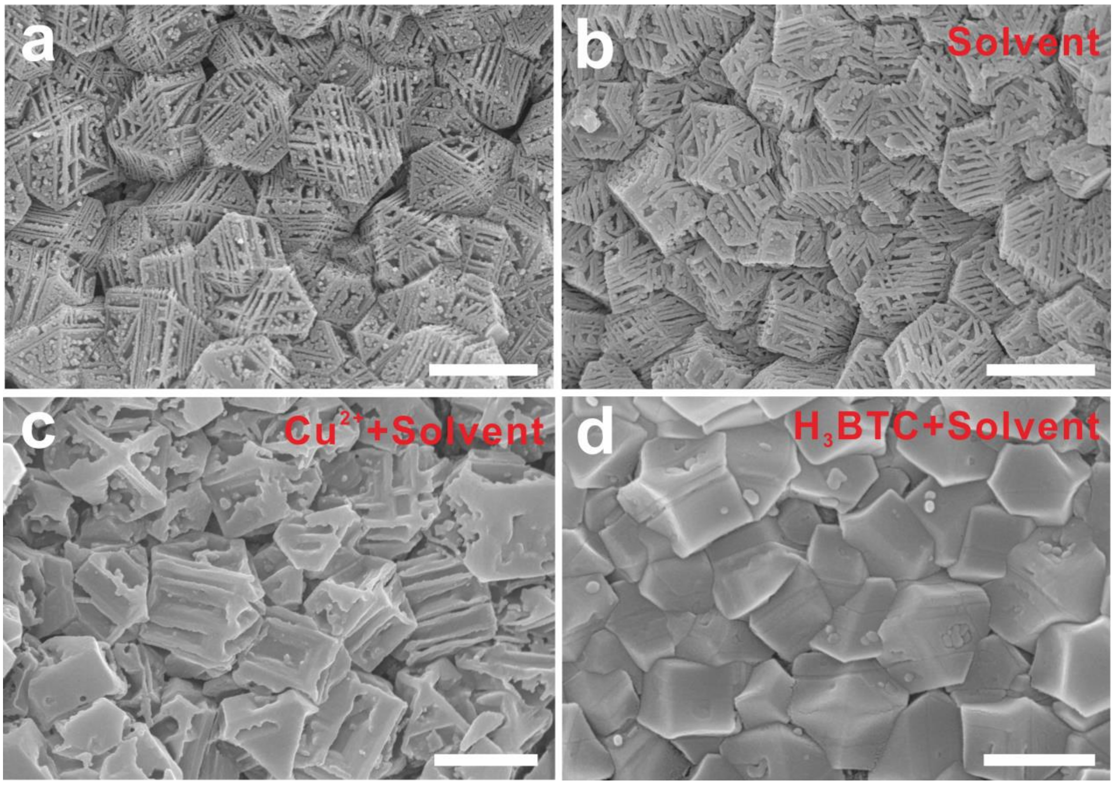

Supplementary Figure 57. SEM images of (a) CASFZU-1; (b) CASFZU-1 thin film was immersed into the mixed solvent (H_2O/ethanol, *v*:*v* = 1:1, 10 mL) for 1h; (c) CASFZU-1 thin film was reacted with 5 mM Cu^{2+} in the same mixed solvent at room temperature; (d) CASFZU-1 thin film was reacted with 5 mM H_3BTC in the same mixed solvent at room temperature. Scale bars, 1 μm for (a), (b), (c) and (d).

Traditionally, reaction between divalent copper ions and H_3BTC needs triethylamine to trigger. However, in this system the coordination reaction between trimesic acid molecules and copper species immediately takes place. These facts not only verify that the under-coordinated copper ions existed on the surface of MOF nanosheets, but also indicate that the under-coordinated copper ions have high activity.

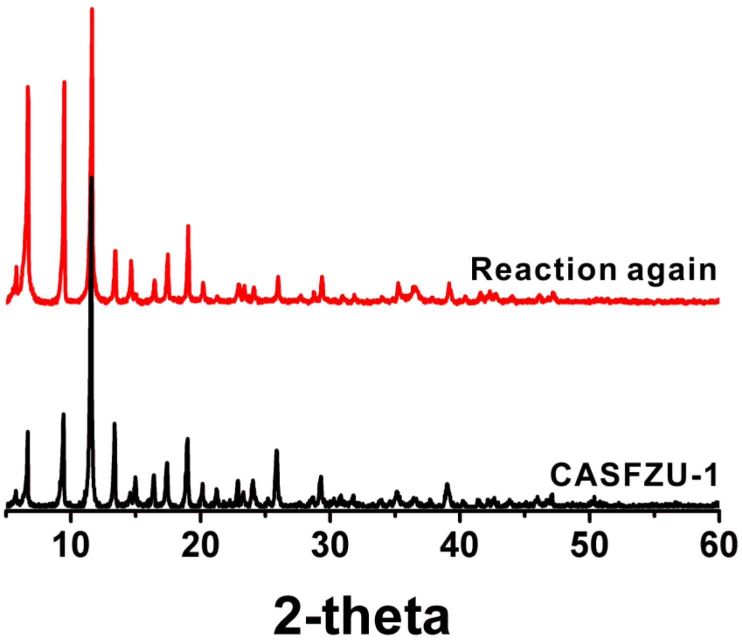

Supplementary Figure 58. PXRD patterns for the CASFZU-1 and CASFZU-1 react with 5mM H_3BTC in mixed solvent (H_2O/ethanol, V:V = 1:1) at room temperature (reaction again).

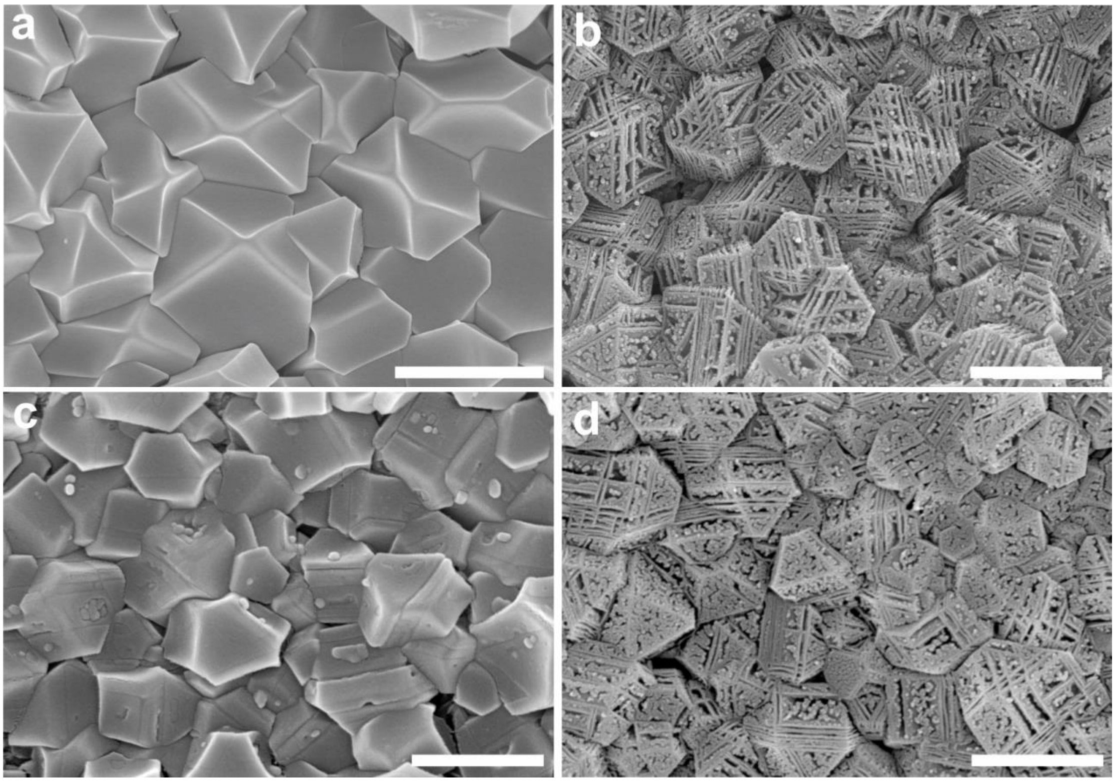

Supplementary Figure 59. SEM images of (a) pristine HKUST-1, (b) CASFZU-1, (c) HKUST-1 (H2) which was produced via CASFZU-1 react with H_3BTC, (d) CASFZU-1 (C2) which was synthesized via etching H2. Scale bars, 2 μm for (a), 1 μm for (b-d), respectively.

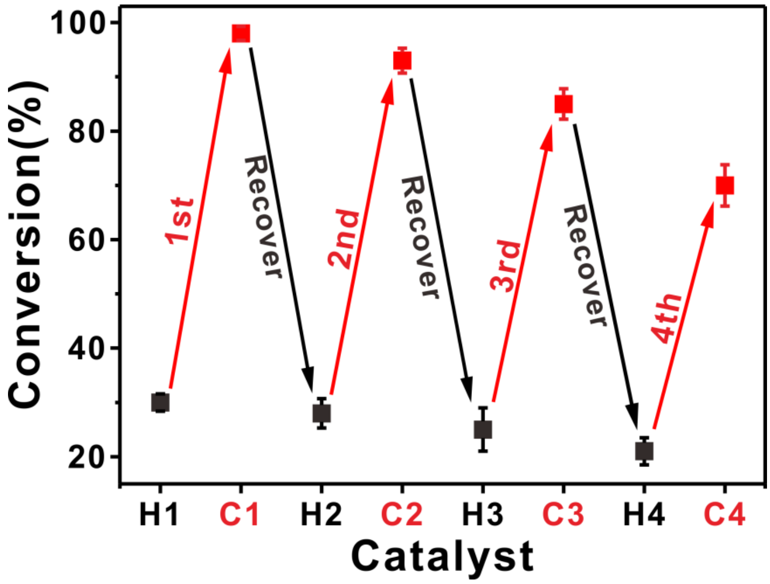

Supplementary Figure 60. Conversions of fixation of CO_2 with 2-ethyloxirane catalyzed by different states of MOFs. It shows that all of the CASFZU-1 (C1, C2, C3, C4) have higher activity than the HKUST-1 (H1, H2, H3, H4), and the CASFZU-1 still exhibits high conversion of 70 ± 3.8% after 4 round etching (C4). Error bars represent the standard deviation of three replicate samples.

HKUST-1 was etching with mixed solvent (H_2O/ethanol, V_{water}:$V_{ethanol}$ = 1:1) at RH 25% for a month. CASFZU-1 thin film was reconstruction with 5 mM H_3BTC in the same mixed solvent (H_2O/CH_3CH_2OH, v/v = 1/1, 10 mL) at room temperature. It exhibits long life-cycle for industrial application with a simple, effective, and low-cost material reconstruction. This route also opens new venues for self-healing of MOF technical forms.

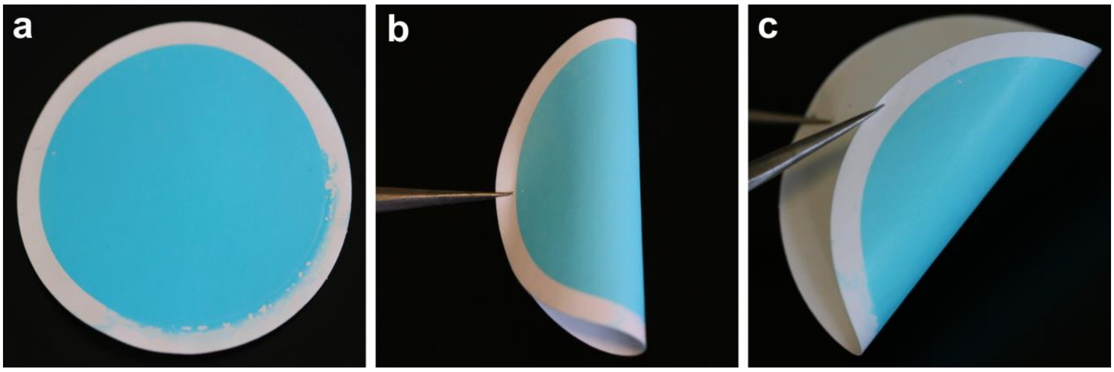

Supplementary Figure 61. Photographs of a 1.5 μm thick bendable CASFZU-1 thin film (shown in Figure 4c.). (a) Free-standing, (b) force folding, (c) partial folding.

Supplementary Table 1. Relevant polarity for solvent.

**solvent**	**polarity**
water	10.2
methanol	6.6
ethanol	4.3
i-propanol	4.3
n-propanol	4
n-butanol	3.7

Supplementary Table 2. Atom percentage measured from the XPS spectra.

Element	Atomic% in HKUST-1	Atomic% in CASFZU-1	Atomic% in random-etching MOFs	Atomic% in Collapsed MOFs
C	60.51	55.47	61.00	59.58
O	33.65	35.23	32.66	35.66
Cu	5.84	9.30	6.34	4.77

Supplementary Table 3. Cu *K*-edge EXAFS curve fitting parameters of HKUST-1 and CASFZU-1 samples before and after dehydration on the basis of hydrated $[Cu_2C_4O_8](H_2O)_2$ moiety [a]. Details on the path assignment are given in Supplementary Figure 29.

sample	Path	N	R (Å)	σ^2 (Å^2)	ΔE_0 (eV)	R_f, %
Cu foil [b]	Cu-Cu1	12	2.54	0.009	2.3	0.3
CuO [b]	Cu-O1	4	1.96	0.005	1.4	0.1
	Cu-O2	2	2.71	0.010		
	Cu-Cu1	8	2.94	0.013		
	Cu-Cu2	2	3.13	0.003		
HKUST-1 [c]	Cu-O1	4	1.97	0.005	-3.7	0.1
	Cu-Ow	1	2.06	0.015		
	Cu-Cu1	1	2.62	0.010		
	Cu-C1	4	2.81	0.015		
	Cu-O2	4	3.15	0.012		
	Cu-C1-O1	8	3.40	0.015		
HKUST-1-dehy [c]	Cu-O1	4	1.96	0.005	-4.1	0.1
	Cu-Ow	0.8	2.01	0.036		
	Cu-Cu1	1	2.59	0.015		
	Cu-C1	4	2.75	0.019		
	Cu-O2	4	3.12	0.016		
	Cu-C1-O1	8	3.35	0.015		
CASFZU-1 [c]	Cu-O1	3.8	1.96	0.005	-4.1	0.1
	Cu-Ow	1.2	2.05	0.018		
	Cu-Cu1	3.8	2.64	0.014		
	Cu-C1	3.8	2.82	0.027		
	Cu-O2	3.8	3.17	0.013		
	Cu-C1-O1	3.8	3.43	0.020		
CASFZU-1-dehy [c]	Cu-O1	3.8	1.95	0.005	-5.9	0.1
	Cu-Ow	0.8	2.03	0.006		
	Cu-Cu1	0.9	2.58	0.011		
	Cu-C1	3.8	2.74	0.010		
	Cu-O2	3.8	3.15	0.018		
	Cu-C1-O1	3.8	3.37	0.010		

[a] N, coordination number; R, distance between absorber and backscatter atoms; σ^2, Debye–Waller factor to account for both thermal and structural disorders; ΔE_0, inner potential correction; R factor (%) indicates the goodness of the fit. Error bounds (accuracies) that characterize the structural parameters obtained by EXAFS spectroscopy were estimated as N ± 20%; R ± 1%; σ^2 ± 20%; ΔE_0 ± 20%. S_0^2 was fixed to 0.95 as determined from CuO reference fitting. Bold numbers indicate fixed coordination number (N) according to the crystal structure. [b] Fitting range: $2.0 \le k$ (/Å) ≤ 12.0 and $1.0 \le R$ (Å) ≤ 3.1. [c] Fitting range: $2.0 \le k$ (/Å) ≤ 12.0 and $0.8 \le R$ (Å) ≤ 3.0.

Supplementary Table 4. Best-fit structural parameters derived from the analysis of the XANES spectra of HKUST-1 and CASFZU-1 samples before and after dehydration performed on the structures of hydrated $[Cu_2C_4O_8](H_2O)_2$ moiety. R_{sq} is the residual function, and errors are given in parentheses. The results of EXAFS and DFT calculation are appended for comparison.

Moiety	Bond	XANES	EXAFS	DFT
HKUST-1	Cu-O1 (Å)	1.98	1.97	2.01
	Cu-Ow (Å)	2.16	2.06	2.18
	Cu-Cu1 (Å)	2.71	2.62	2.72
	Cu-C1 (Å)	2.91	2.81	2.91
	Cu-O2 (Å)	3.14	3.15	3.18
	R_{sq}	1.1	-	-
HKUST-1-dehy	Cu-O1 (Å)	1.96	1.96	1.98
	Cu-Ow (Å)	2.13	2.01	-
	Cu-Cu1 (Å)	2.65	2.59	2.59
	Cu-C1 (Å)	2.84	2.75	2.87
	Cu-O2 (Å)	3.07	3.12	3.13
	R_{sq}	0.8	-	-
CASFZU-1	Cu-O1 (Å)	1.98	1.96	2.02
	Cu-Ow (Å)	2.15	2.05	2.10
	Cu-Cu1 (Å)	2.75	2.64	2.74
	Cu-C1 (Å)	2.91	2.82	2.93
	Cu-O2 (Å)	3.14	3.17	3.21
	R_{sq}	0.4	-	-
CASFZU-1-dehy	Cu-O1 (Å)	1.96	1.95	1.96
	Cu-Ow (Å)	2.13	2.03	1.81
	Cu-Cu1 (Å)	2.68	2.58	2.67
	Cu-C1 (Å)	2.84	2.74	2.86
	Cu-O2 (Å)	3.08	3.15	3.13
	R_{sq}	1.1	-	-

Supplementary Table 5. Summary of CO_2 and N_2 uptake at 1atm and 298 K.

Materials	CO_2 uptake (cm^3g^{-1})	N_2 uptake (cm^3g^{-1})	Selectivity of V_{CO2}/V_{N2}
HKUST-1	65.51	4.84	13.55
CASFZU-1	60.89	3.23	18.88

HKUST-1 exhibited CO_2/N_2 adsorption selectivity of 13.6 while CASFZU-1 exhibited much higher CO_2/N_2 adsorption selectivity of 18.9. This result demonstrates the stronger adsorption capacity of CASFZU-1 for the selective sorption of CO_2 from the low CO_2 concentration atmosphere, so it can have higher potential application for carbon fixation in nature.

Supplementary Table 6. Summary of molecules sizes of epoxides with different branched R chain from Chem.3D.

Entry	R	kinetic diameter† (nm)
1	CH_3	0.43
2	CH_3CH_2	0.53
3	CH_3Cl	0.44
4	CH_2Br	0.45
5	$CH_3(CH_2)_7$	1.17
6	$CH_3(CH_2)_9$	1.49

Supplementary Table 7. Results of the cycloaddition reaction of CO_2 with 2-methyloxirane over various catalysts.

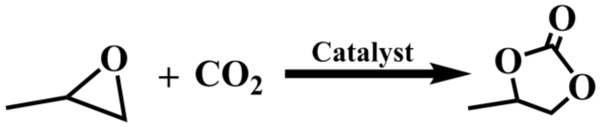

Entry	Catalyst	Condition	TOF/h^{-1}	reference
1	CASFZU-1	r.t./ 1atm	54	This work
2	bismuth compounds	25°C/1atm	33.5	22
3	Triazole-Containing MOF	25°C/1atm	20	23
4	YCl_3	25°C/1atm	18.8	24
5	MMCF-2	25°C/1atm	15.9	25
6	HKUST-1	r.t./ 1atm	15.5	This work
7	MMPF-9	25°C/1atm	14.6	25
8	Dimetallic aluminium(salen) complexes	25°C/1atm	10.3	26
9	bimetallic iron catalyst	25°C/1atm	4	27
10	Salen-M (M = Co, Al, Zn)	25°C/1atm	3.2	28
11	phosphorus ylide	25°C/1atm	3	29

The TOF was 54.0 h^{-1} per CASFZU-1 for catalytic cycloaddition reaction of CO_2 with 2-methyloxirane. To our best knowledge, The CASFZU-1 has higher catalytic efficiency than most of the catalysts reported in catalytic cycloaddition reaction of CO_2 with 2-methyloxirane.

Supplementary Table 8. Results of the cycloaddition reaction of CO_2 with 2-ethyloxirane over various catalysts.

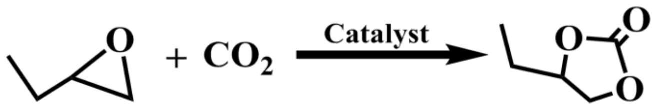

Entry	Catalyst	Condition	TOF/h^{-1}	reference
1	CASFZU-1	r.t./ 1atm	32.7	This work
2	Triazole-Containing MOF	25°C/1atm	17.3	23
3	HKUST-1	r.t./ 1atm	10	This work
4	Acylamide-Containing MOF	25°C/1atm	8.85	30
5	Co(II)-based MOF	25°C/1atm	5.58	31
6	$[Cu_2(L1)]_n$	25°C/1atm	2.5	32

Supplementary Table 9. Results of the cycloaddition reaction of CO_2 with 1,2-epoxydodecane over various catalysts.

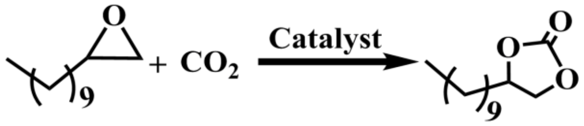

Entry	Catalyst	Condition	TOF/h^{-1}	reference
1	CASFZU-1	r.t./ 1atm	25.34	This work
2	HKUST-1	r.t./ 1atm	1.67	This work
3	Triazole-Containing MOF	25°C/1atm	1.25	23
4	Acylamide-Containing MOF	25°C/1atm	0.73	30

For cycloaddition reaction of CO_2 with 1,2-epoxydodecane (represent big molecule reaction substrates), the TOF was 25.34 h^{-1} per CASFZU-1 at r.t./1 atm which is much higher than other catalyst previous reported.

Supplementary References

1. Tranchemontagne, D. J., Hunt, J. R. & Yaghi, O. M. Room temperature synthesis of metal-organic frameworks: MOF-5, MOF-74, MOF-177, MOF-199, and IRMOF-0. *Tetrahcdron* **64**, 8553-8557, (2008).
2. Rodenas, T. *et al.* Metal–organic framework nanosheets in polymer composite materials for gas separation. *Nat. mater.* **14**, 48-55, (2015).
3. Torad, N. L. *et al.* Facile synthesis of nanoporous carbons with controlled particle sizes by direct carbonization of monodispersed ZIF-8 crystals. *Chem. Commun.* **49**, 2521-2523, (2013).
4. Volkringer, C. *et al.* A microdiffraction set-up for nanoporous metal–organic framework-type solids. *Nat. mater.* **6**, 760-764, (2007).
5. Wang, R. *et al.* Quasi–Polymeric Metal–Organic Framework UiO-66/g-C_3N_4 Heterojunctions for Enhanced Photocatalytic Hydrogen Evolution under Visible Light Irradiation. *Adv. Mater. Interfaces* **2**, 1500037, (2015).
6. Loiseau, T. *et al.* A Rationale for the Large Breathing of the Porous Aluminum Terephthalate (MIL-53) Upon Hydration. *Chem.–Eur. J.* **10**, 1373-1382, (2004).
7. Millange F, Serre C, Férey G. Synthesis, structure determination and properties of MIL-53as and MIL-53ht: the first Cr^{III} hybrid inorganic–organic microporous solids: $Cr^{III}(OH)\cdot\{O_2C–C_6H_4–CO_2\}\cdot\{HO_2C–C_6H_4–CO_2H\}_x$. *Chem. Commun*. **8**, 822-823 (2002).
8. Xia, B. Y. *et al.* A metal–organic framework-derived bifunctional oxygen electrocatalyst. *Nat. Energy* **1**, 15006, (2016).
9. Xu, X., Cao, R., Jeong, S. & Cho, J. Spindle-like mesoporous α-Fe_2O_3 anode material prepared from MOF template for high-rate lithium batteries. *Nano Lett.* **12**, 4988-4991, (2012).
10. Becke, A. D. Density–functional thermochemistry. III. The role of exact exchange. *J. Chem. Phys.* **98**, 5648-5652, (1993).
11. Perdew, J. P., Burke, K. & Wang, Y. Generalized gradient approximation for the exchange-correlation hole of a many-electron system. *Phys. Rev. B* **54**, 16533-16539, (1996).
12. Fukui, K. Formulation of the reaction coordinate. *J. Phys. Chem.* **74**, 4161-4163, (1970).
13. Frisch, M. *et al.* J. Gaussian 09, Revision B. 01, Gaussian. *Inc., Wallingford, CT*, (2010).
14. Dennington, R., Keith, T. & Millam, J. GaussView, version 5. *Semichem Inc., Shawnee Mission, KS*, (2009).
15. Lu, T. & Chen, F. Multiwfn: a multifunctional wavefunction analyzer. *J. Comput. Chem.* **33**, 580-592, (2012).
16. Low, J. J. *et al.* Virtual high throughput screening confirmed experimentally: porous coordination polymer hydration. *J. Am. Chem. Soc.* **131**, 15834-15842, (2009).
17. Drenchev, N., Ivanova, E., Mihaylov, M. & Hadjiivanov, K. CO as an IR probe molecule for characterization of copper ions in a basolite C300 MOF sample. *Phys. Chem. Chem. Phys.*, **12**, 6423-6427, (2010).

18. Majano, G. et al. Solvent–Mediated Reconstruction of the Metal–Organic Framework HKUST-1 ($Cu_3(BTC)_2$). *Adv. Funct. Mater.* **24**, 3855-3865, (2014).
19. Prestipino, C. *et al.* Local Structure of Framework Cu(II) in HKUST-1 Metallorganic Framework: Spectroscopic Characterization upon Activation and Interaction with Adsorbates. *Chem. Mater.* **18**, 1337-1346, (2006).
20. North, M. & Pasquale, R. Mechanism of cyclic carbonate synthesis from epoxides and CO_2. *Angew. Chem., Int. Ed.* **121**, 2990-2992, (2009).
21. Grace, J., Lloyd, J., McIntyre, J. & Miranda, A. C. Carbon dioxide uptake by an undisturbed tropical rain forest in southwest Amazonia, 1992 to 1993. *Science* **270**, 778-780, (1995).
22. Yin, S.-F. & Shimada, S. Synthesis and structure of bismuth compounds bearing a sulfur-bridged bis(phenolato) ligand and their catalytic application to the solvent-free synthesis of propylene carbonate from CO_2 and propylene oxide. *Chem. Commun.,* **9**,1136-1138, (2009).
23. Li, P.-Z. et al. A Triazole-Containing Metal–Organic Framework as a Highly Effective and Substrate Size-Dependent Catalyst for CO_2 Conversion. *J. Am. Chem. Soc.* **138,** 2142-2145, (2016).
24. Guillerm, V. et al. Discovery and introduction of a (3,18)-connected net as an ideal blueprint for the design of metal–organic frameworks. *Nat. Chem.* **6**, 673-680, (2014).
25. Gao, W.-Y. et al. Crystal Engineering of an nbo Topology Metal–Organic Framework for Chemical Fixation of CO_2 under Ambient Conditions. *Angew. Chem., Int. Ed.* **53**, 2615-2619, (2014).
26. Meléndez, J., North, M. & Pasquale, R. Synthesis of Cyclic Carbonates from Atmospheric Pressure Carbon Dioxide Using Exceptionally Active Aluminium(salen) Complexes as Catalysts. *Eur. J. Inorg. Chem.* **2007**, 3323-3326, (2007).
27. Buchard, A., Kember, M. R., Sandeman, K. G. & Williams, C. K. A bimetallic iron(iii) catalyst for CO_2/epoxide coupling. *Chem. Commun.* **47**, 212-214, (2011).
28. Wang, T.-T., Xie, Y. & Deng, W.-Q. Reaction Mechanism of Epoxide Cycloaddition to CO_2 Catalyzed by Salen-M (M = Co, Al, Zn). *J. Phys. Chem. A.* **118**, 9239-9243, (2014).
29. Zhou, H., Wang, G.-X., Zhang, W.-Z. & Lu, X.-B. CO_2 Adducts of Phosphorus Ylides: Highly Active Organocatalysts for Carbon Dioxide Transformation. *ACS Catal.* **5**, 6773-6779, (2015).
30. Li, P.-Z. et al. Highly Effective Carbon Fixation via Catalytic Conversion of CO_2 by an Acylamide-Containing Metal–Organic Framework. *Chem. Mater.* **29**, 9256-9261, (2017).
31. Wang, H.-H. et al. Porous MOF with Highly Efficient Selectivity and Chemical Conversion for CO_2. *ACS Appl. Mater. Interfaces.* **9**, 17969-17976, (2017).
32. Li, P.-Z. et al. Two metal–organic frameworks sharing the same basic framework show distinct interpenetration degrees and different performances in CO_2 catalytic conversion. *CrystEngComm.* **19**, 4157-4161, (2017).
